# Analysis of the impact of success on three dimensions of sustainability in 173 countries

**DOI:** 10.1038/s41598-022-19131-6

**Published:** 2022-08-30

**Authors:** A. Kaklauskas, L. Kaklauskiene

**Affiliations:** Vilnius Gediminas Technical University, Sauletekio Aveniu 11, Vilnius, Lithuania

**Keywords:** Ecological modelling, Climate-change impacts, Climate-change policy, Environmental impact, Socioeconomic scenarios, Sustainability, Statistics

## Abstract

The United Nations have announced 17 Sustainable Development Goals and 169 targets, which are indivisible and integrated, and which balance the economic, social, and environmental dimensions of sustainable development. This indicates that the performance of successful nations is generally good across many sustainability indicators. Our results, based on multi-criteria and statistical analysis across 173 countries, suggest an interconnection between a country’s sustainability 12 indicators and success. This article focuses on the Country Success and Sustainability (CSS) Maps and Models of the World, which show that improvements in environmental, social, and economic sustainability indicators lead to improvements in the country's success, and vice versa. The CSS Models explain 98.2% of national success and 80.8% of the three dimensions of average sustainability dispersions. When a nation’s success increases by 1%, the 12 indicators of the three dimensions of sustainability improve by 0.85% on average. The human development index and GDP per capita were the success variables with the most substantial impact on 12 sustainability indicators in 173 countries. Calculations made using equal and different weights of 17 criteria show a deviation of 5.34% for the priorities of these 173 countries.

## Introduction

The United Nations have set a goal to achieve sustainable economic, social, and environmental development. The actions to achieve this goal should be balanced and integrated. In sustainable development, preserving the planet, combating inequality within and among countries, eradicating poverty in all its forms and dimensions, fostering social inclusion, and creating continuous, sustainable, and inclusive economic growth are all interdependent aspects linked to each other^1^. Highlighting key results describing the effects of climate on migration, demographics, economics, agriculture, ineguality, health, disasters, and conflict, various researchers^2–5^ suggest that climate is an important aspect that has influenced the many global challenges and even the historical evolution of the global economy. Moreover, poor air quality can contribute to shorter lifespans^6^. Globally, outdoor air pollution causes 3.3 million premature deaths per year, according to the 2010 data on the global burden of disease^7^.

Ridley et al.^8^ have indicated a bidirectional causal relationship between poverty and mental illness, and have attempted to determine what mechanisms are at work when poverty triggers mental illness, as well as how mental illness leads to even deeper poverty. Research suggests that stress can contribute to faster aging. It has also been linked with the spread of cancer, diabetes, heart disease, and other chronic illnesses in individuals exposed to stress over long periods of time. Physiological responses can start at an early age^9^.

All elements tend to move together in the modern world: economic institutions, political institutions, human capital, prosperity, and numerous others^10^. Chang and Lee^11^ have examined panel data for 23 OECD countries for the period between 1970 and 2006 in order to re-appraise the way economic growth, the overall globalization index—including its three main dimensions (political, social, and economic integrations)—move together, and are linked by causal relationships. Stiglitz^12^ points out that generally, higher per capita incomes are accompanied by higher national social indicators. A long‐term relationship between democratic politics, national sovereignty, and economic integration, as proposed in the political globalization trilemma, is supported by econometric evidence^13^.

Materialistic lifestyles and values have been associated with adverse effects on human health, as well as on that of our planet. Therefore, activities and lifestyles should be identified that promote human well-being, at the same time as protecting ecological security^14^. The authors of the current study identify optimal activities (arts and crafts, reading, sports, and meditating) as producing high levels of human well-being with low environmental costs.

This research investigates interrelationships between 17 success and 12 sustainability indicators of 173 countries. Investigations of links between success and sustainability indicators from different countries have been performed, and some systemic reviews describing such studies were also available (Supplementary sections 1, 4, and 5). However, our search did not return any studies that attempted to outline the impact of country success by examining the entire system of sustainability indicators. Our research, for instance, has shown that the 17 indicators of success and the 12 indicators of the three dimensions of sustainability are very closely interrelated, making up a single space, and all have to be analyzed in an integrated manner.

Successful forecasts of success and sustainability indicators are possible, as indicated by the studies performed by these authors and other researchers because specific social, economic, political, and environmental indicators usually correlate with each other. Both CSS Maps and Models developed by this research can assist with identifying policy recommendations on ways to improve the micro, meso, and macro environment. The CSS Models can be an alternative response strategy, while providing valuable input for new or revised national health and environmental policies in order to improve country success and sustainability.

This research is a quantitative study to assess the impact of national success on three dimensions of 12 sustainability indicators in 173 countries in 2020, or for the latest data available.

Our CSS Maps and Models are important investigation methods which facilitate the study of interdependence between country success and sustainability. We have performed literature analysis, presented in Supplementary sections 1, 4, and 5, to better investigate the relationship^5,8,15–20^ between our CSS Maps and Models and their components in the worldwide research context.

This research validated the two hypotheses put forward by this study:

- Hypothesis 1—The increasing success of a country is generally accompanied by increasing values for the three dimensions of sustainability indicators, and declines in these indicators lead to decreases in the country's success. Improving some sustainability indicators tends to improve other sustainability indicators.
- Hypothesis 2—Changes in the number of countries and their traditional key indicator systems and weights do not make a very significant difference to the relative national sustainability and success values. Likewise, the boundaries of the seven country clusters discussed in this research do not excessively depend on specific traditional key systems of indicators used in their analysis.

## Results

**Figure 1:**
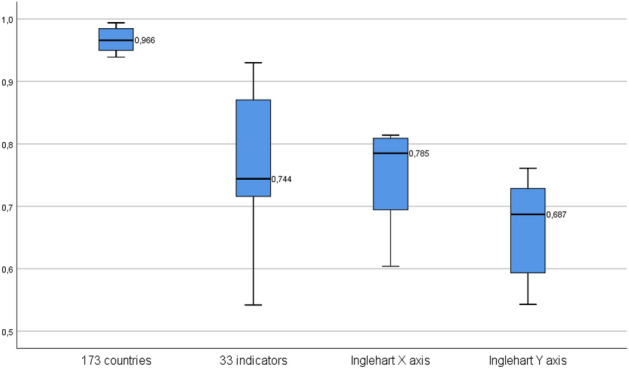
Distribution of correlation coefficients on the CSS Map.

Initially, we determined if significant correlations were present. A practical comparison was performed for this purpose between the three dimensions of sustainability and countries’ success, using the CSS Maps, and the 2020 Inglehart–Welzel Cultural Map of the World (the survival versus self-expression values and the traditional versus secular–rational values)^21^. We analyze the seven sustainability indicators’ vertical y-axes dimensions and the two success and priority horizontal x-axes dimensions (9 CSS Maps’ dimensions). The median correlation between the 9 CSS Map dimensions (the x and y axes) and the survival versus self-expression values are moderate, and the median correlation between the 9 CSS Map dimensions and the traditional versus secular–rational values is strong (Fig. 1). The Shapiro–Wilk Test revealed that the distribution of the values of all variables did not follow the normal law of distribution (p < 0.01). The correlations among the variables were assessed using the Spearman correlation coefficient, and the results are presented in Supplementary Table S3. The correlation analysis determined moderate, high, and very high correlations (p < 0.01) between most selected variables. Figure 1 and Supplementary Table S3 show the moderate overall median of all correlation coefficients between the 33 indicators (17 country success, 12 sustainability, estimated country success and its priority, the 2020 Inglehart–Welzel Cultural Map of the World survival versus self-expression values [x-axis], and the traditional versus secular-rational values [y-axis]). The same figure and table present the moderate and strong correlations median between the nine CSS Map dimensions (the x-axis and y-axis) and the survival versus self-expression values and the traditional versus secular–rational values. Figure 1 and Supplementary Table S4 show the strong median correlation between the 173 countries. This indicates a close interrelation between the variables; therefore one can be exchanged for the other. Supplementary Tables S3 and S4 confirm that the eight-country clusters used in the Inglehart–Welzel Cultural Map of the World can also be applied in the CSS Maps to indicate country success and the three dimensions of sustainability. The correlation between the dimensions characterizing the selected countries from the CSS Models (Supplementary section 3) being analyzed makes this possible.

This research analyzed the dispersions of the values of the given variables and the interdependencies between the variables. We used a multiple linear regression model to measure the way the selected 17 country success criteria influence the independent sustainability variable dispersion. The multiple linear regressions were completed with the help of IBM SPSS V.26 to develop 12 regression models dealing with national success and sustainability. These relationships, determined by the research, correspond to various research findings^22–26^ from around the world. These models can have significant consequences and implications for legislators, policymakers, communities, and businesses.

The CSS Maps offer an easy way to visualize the interrelationships between the success and 12 sustainability indicators of 173 countries for the given period, while the CSS Models enable statistical analysis of these relationships from multiple perspectives, as well as forecasts based on country success and sustainability indicators. This means that the CSS Models offer a more granular analysis of the research object, by means of statistical and multiple criteria analysis methods, whereas the maps focus more on the visual presentation of the results. Over time, the pool of appropriate available data is growing, as is the body of knowledge offered by studies performed in various countries. Therefore, the CSS Models are continuously improved, reflect the real picture in an ever better way, and can be an efficient tool for political decisions. Although the CSS Maps are very helpful at the start of analysis as a quick visualization of the interrelationships between the current status of country success and 12 sustainability indicators, they are static, and offer only limited possibilities for improvement. The CSS Maps can also be analyzed using statistical and multiple criteria analysis. Yet, this analysis will not be as comprehensive as the one offered by the CSS Models because the number of analysis criteria will be considerably smaller. The CSS Maps are integrated with the CSS Models to make sure the map and interdependencies can change over time. This enables the real-time use of the available global practice, a simplified and detailed country success and sustainability indicator interdependence system.

One directional^27–29^ and bidirectional^30–33^ causal relationships between indicators related to country success and sustainability have been examined in various studies around the world. The Country Success and Sustainability (CSS) Maps of the World that have developed as part of this study are described below.

**Figure 2:**
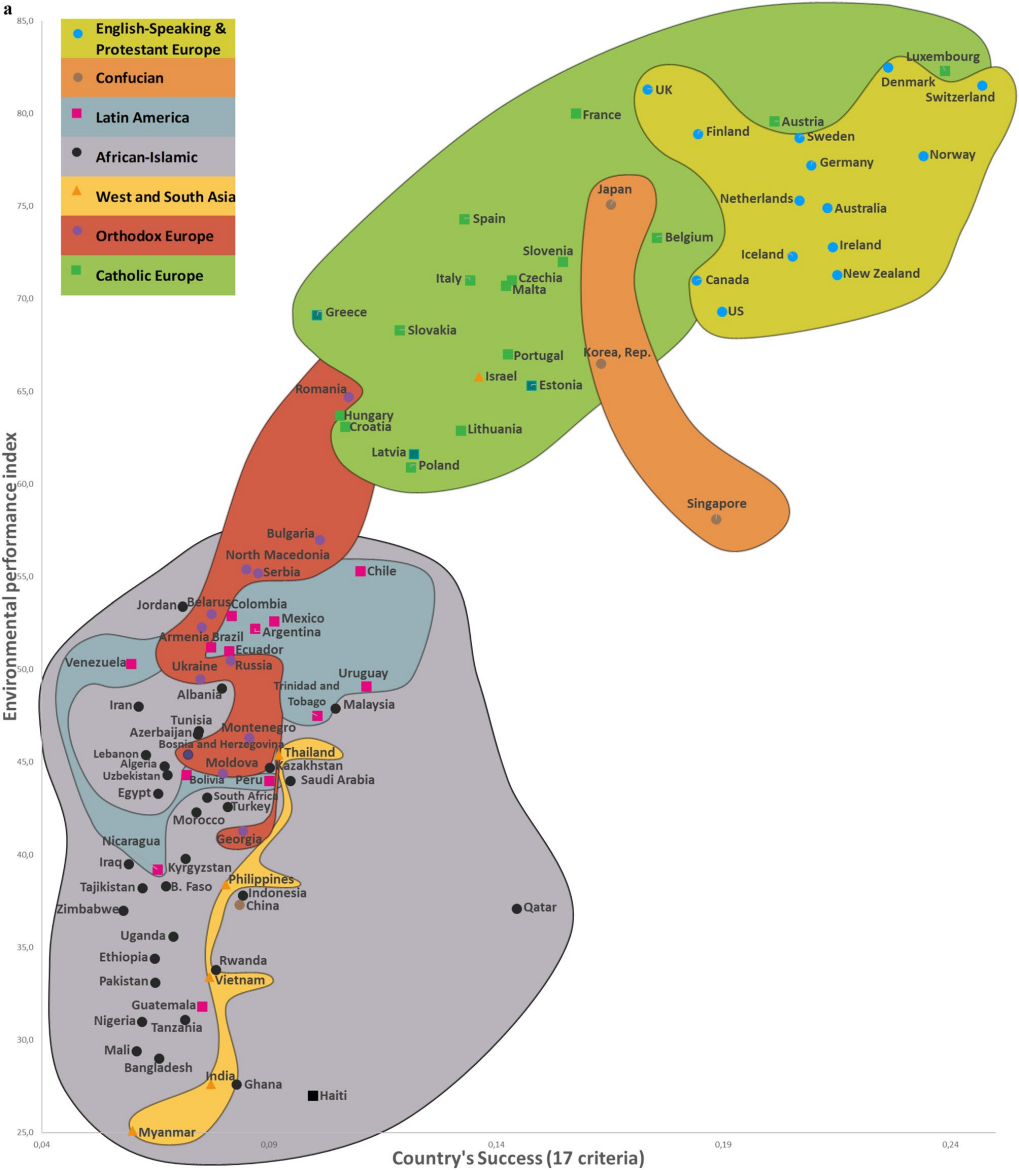

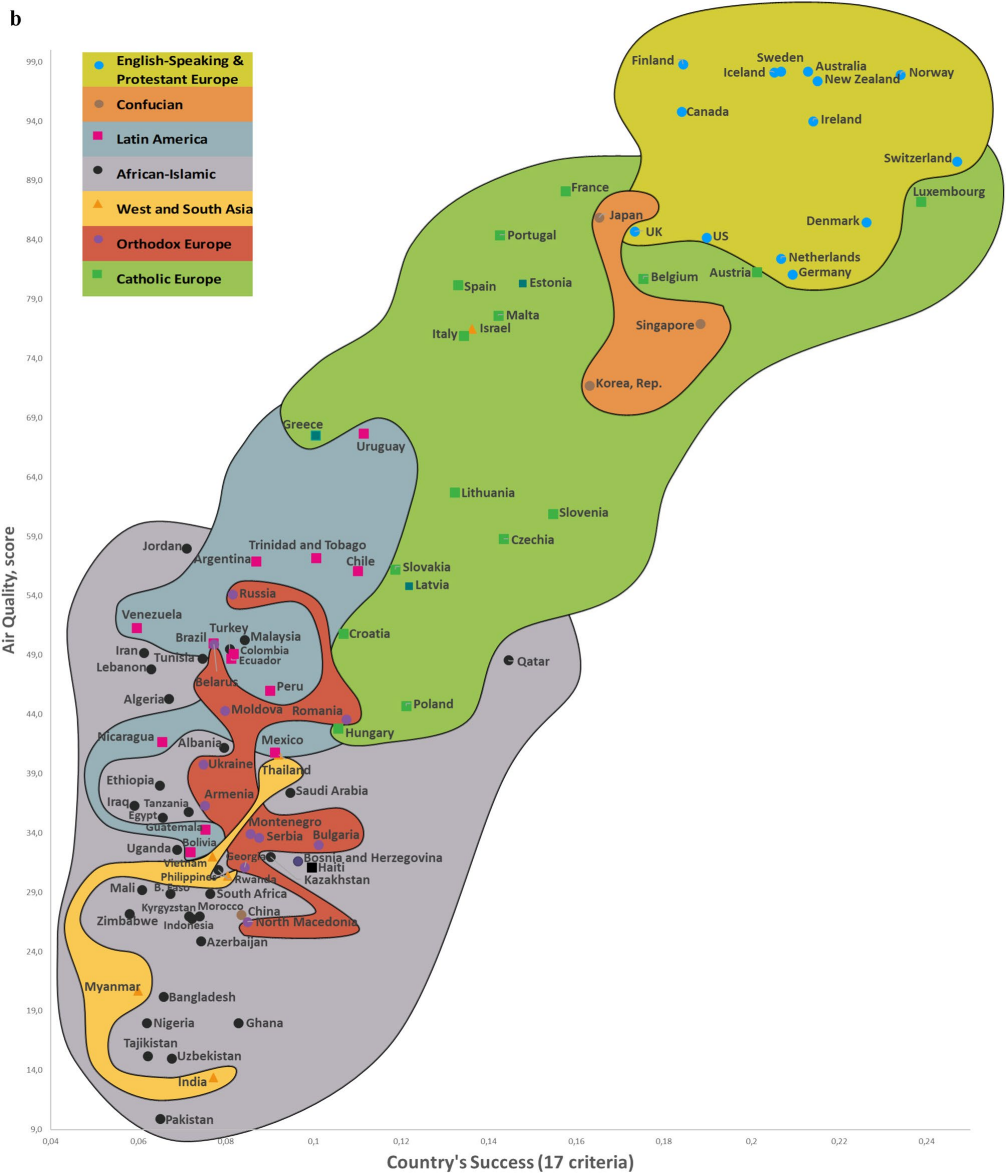

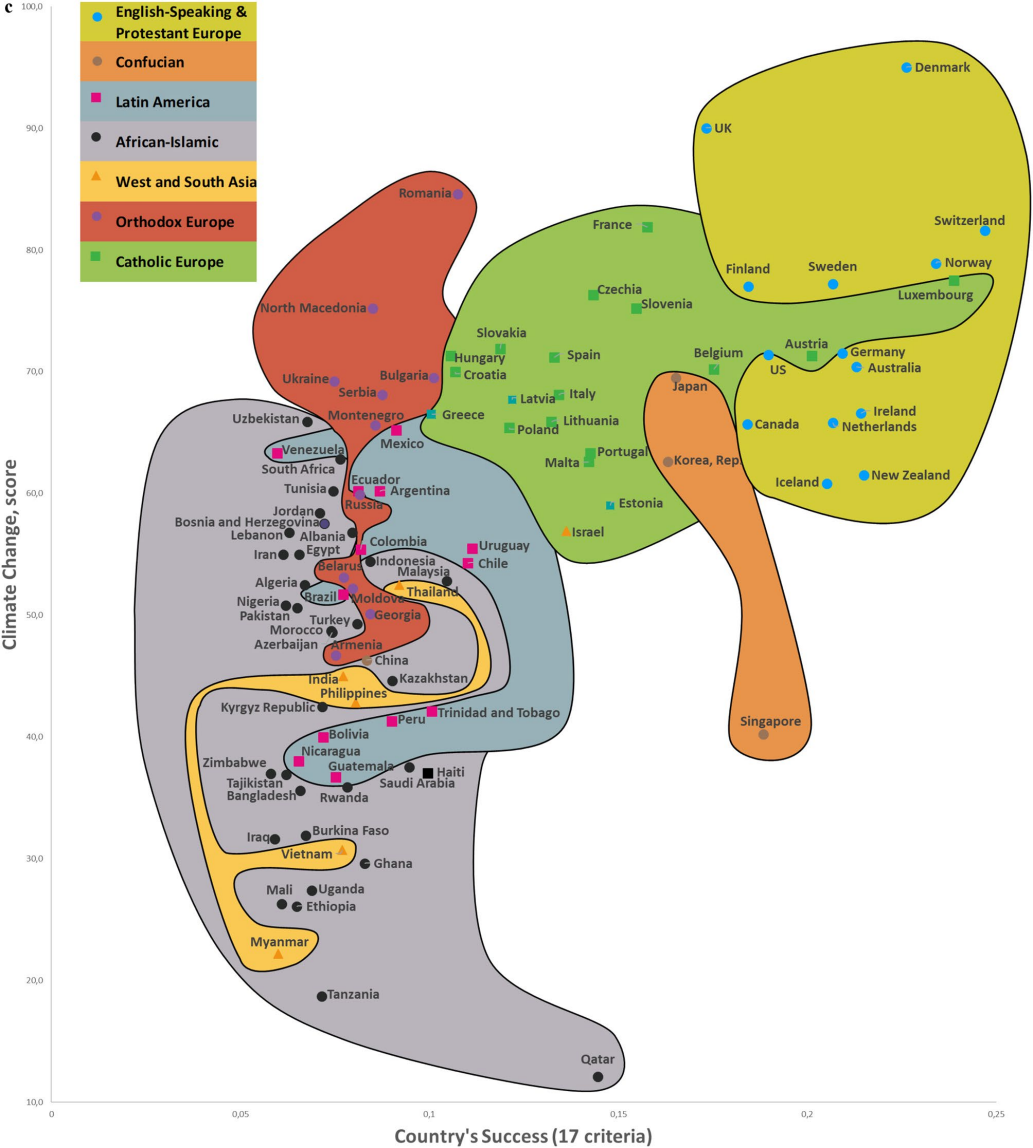
Country success and environment sustainability world maps: (**a**) CSS EPI map. (**b**) CSS air quality map. (**c**) CSS climate change map.

**Figure 3:**
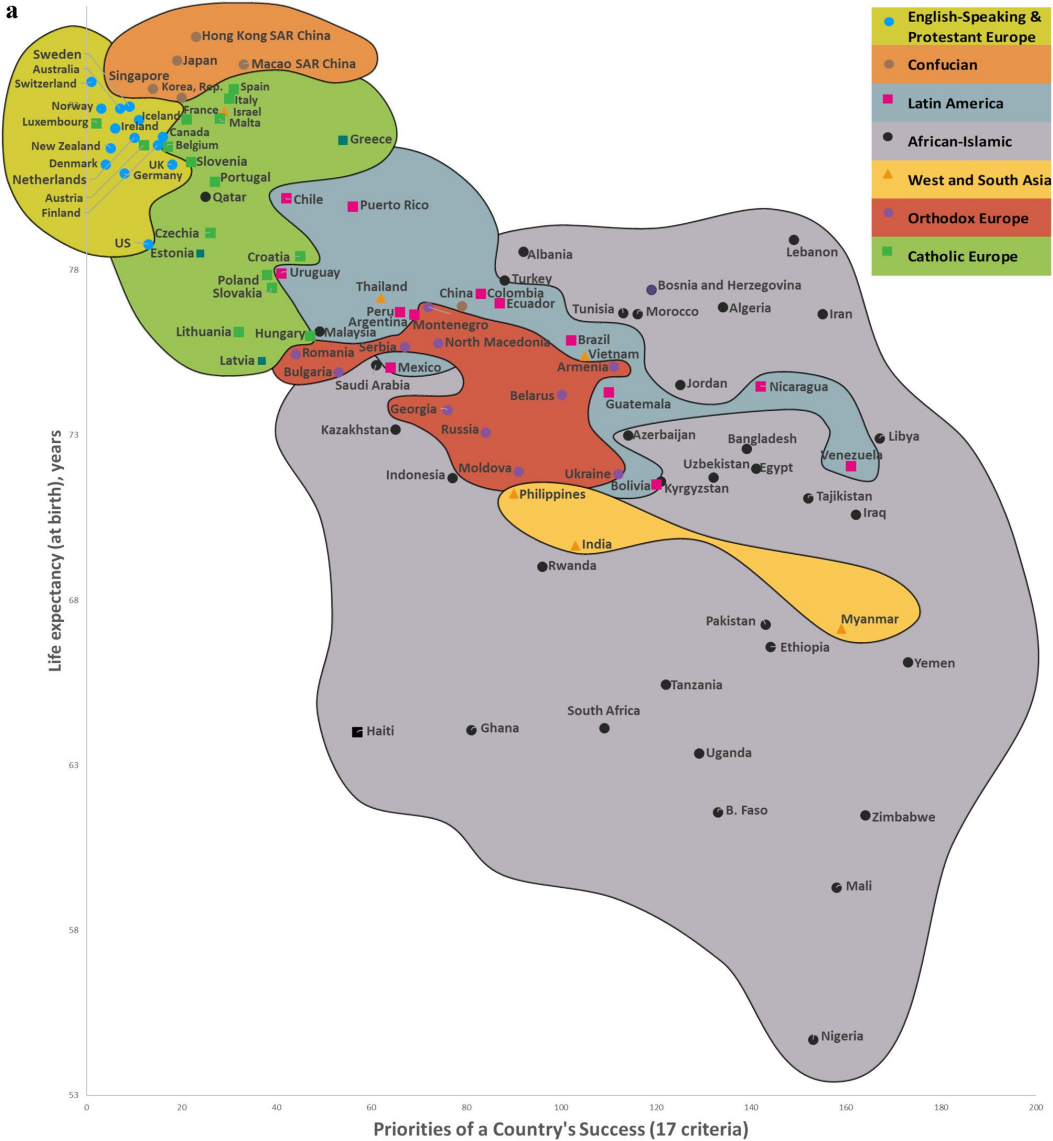

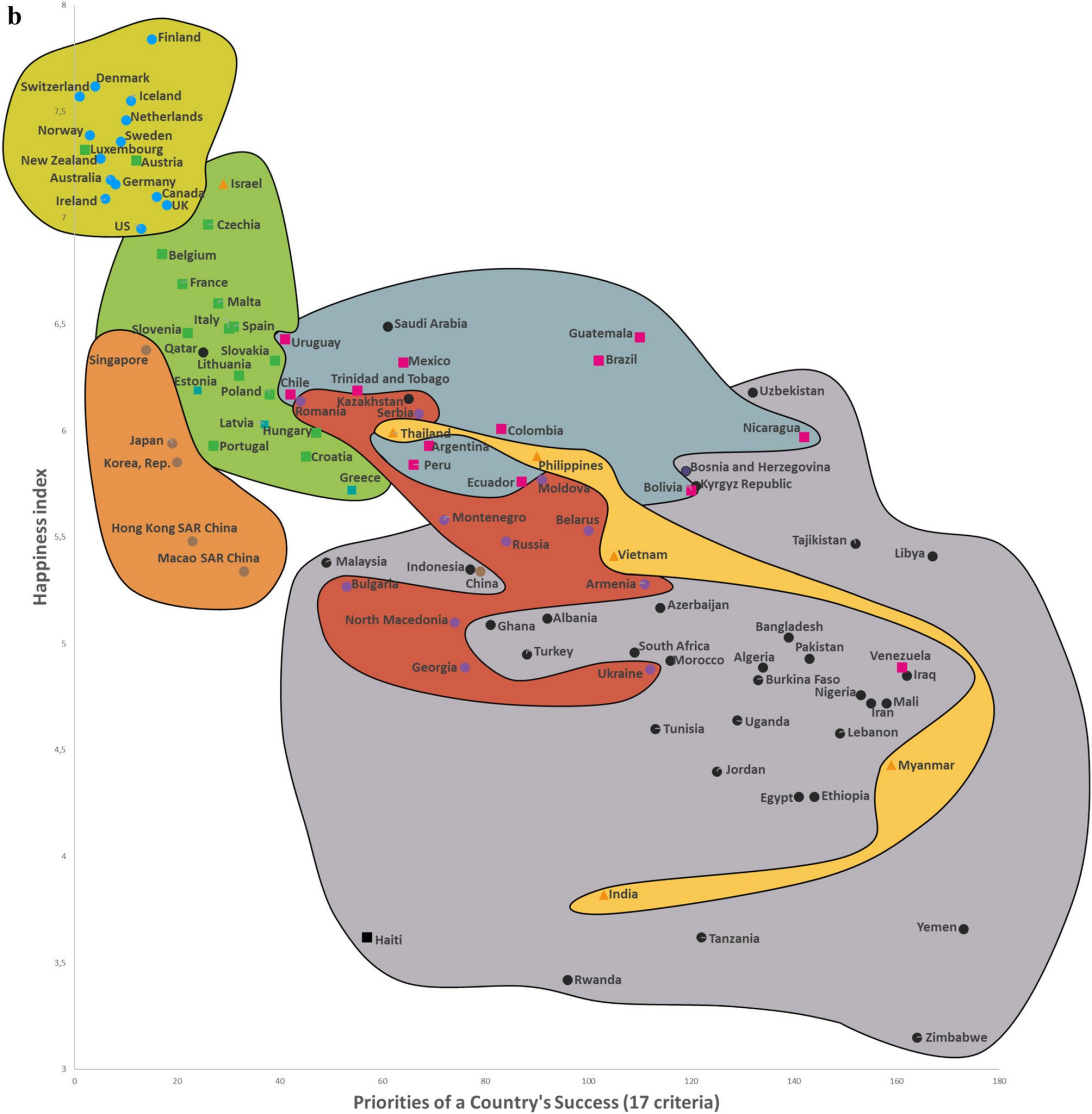

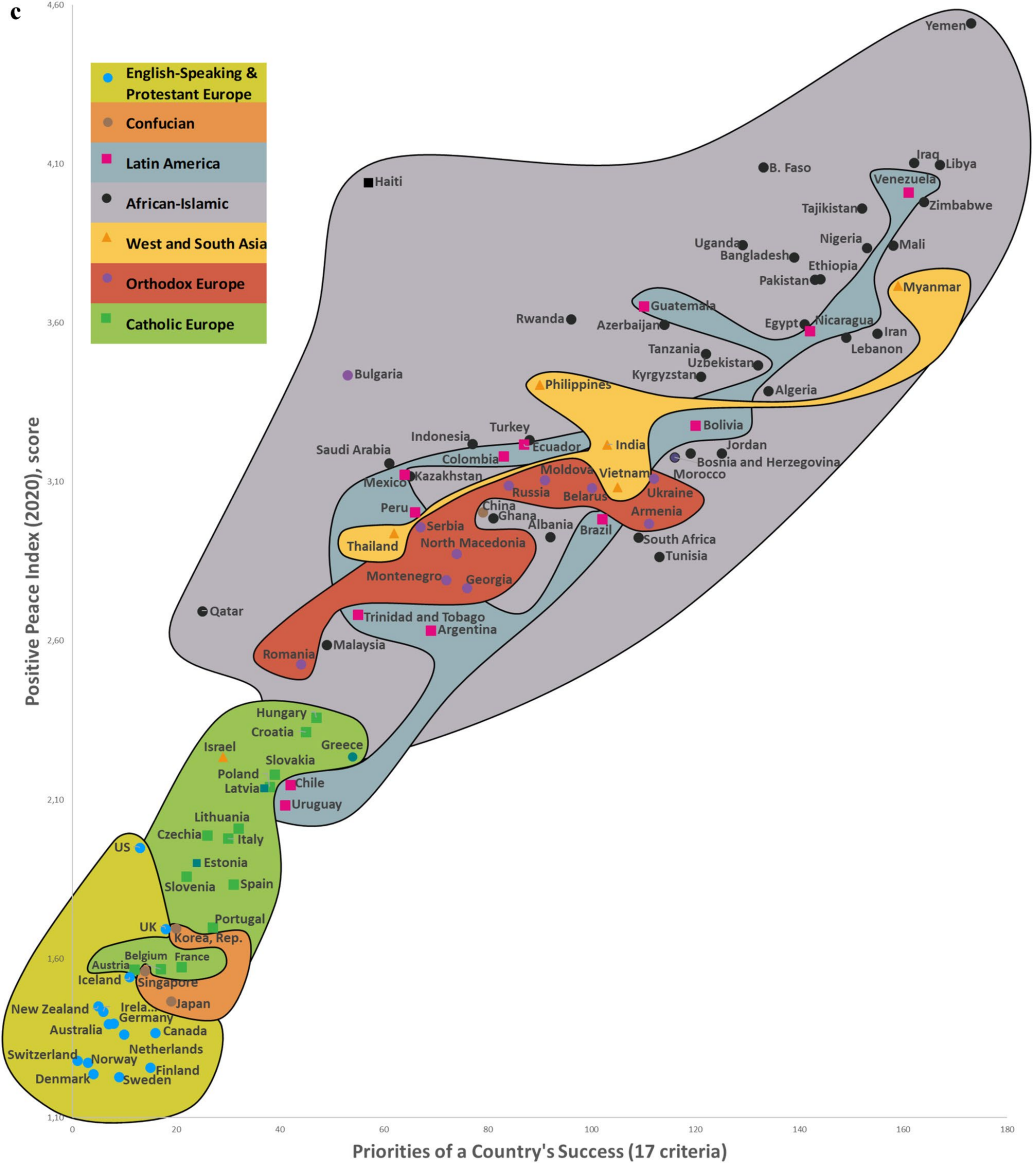
Country success and social sustainability world maps: (**a**) CSS life expectancy at birth map. (**b**) CSS happiness index map. (**c**) CSS positive peace index map.

**Figure 4:**
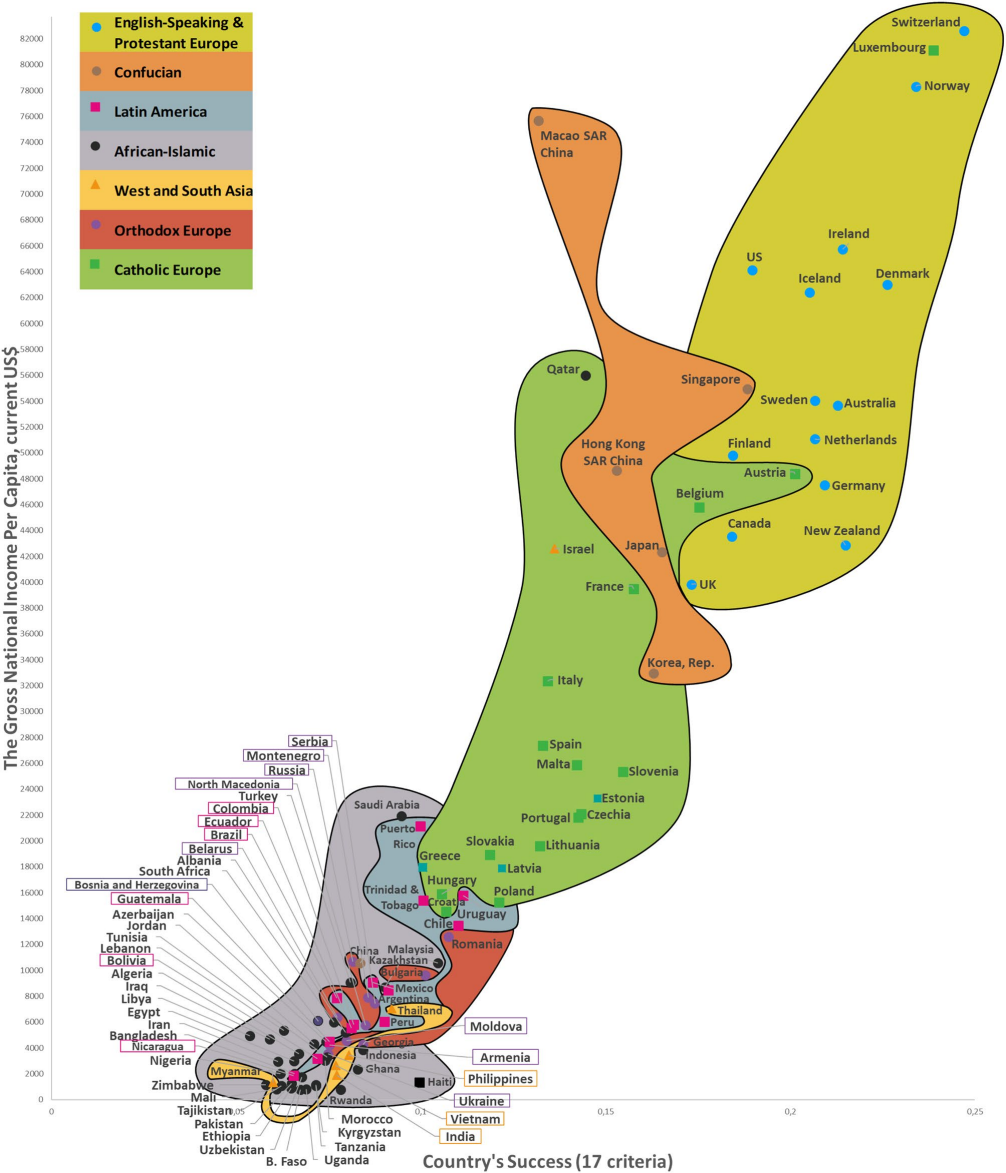
CSS gross national income per capita map.

**Figure 5:**
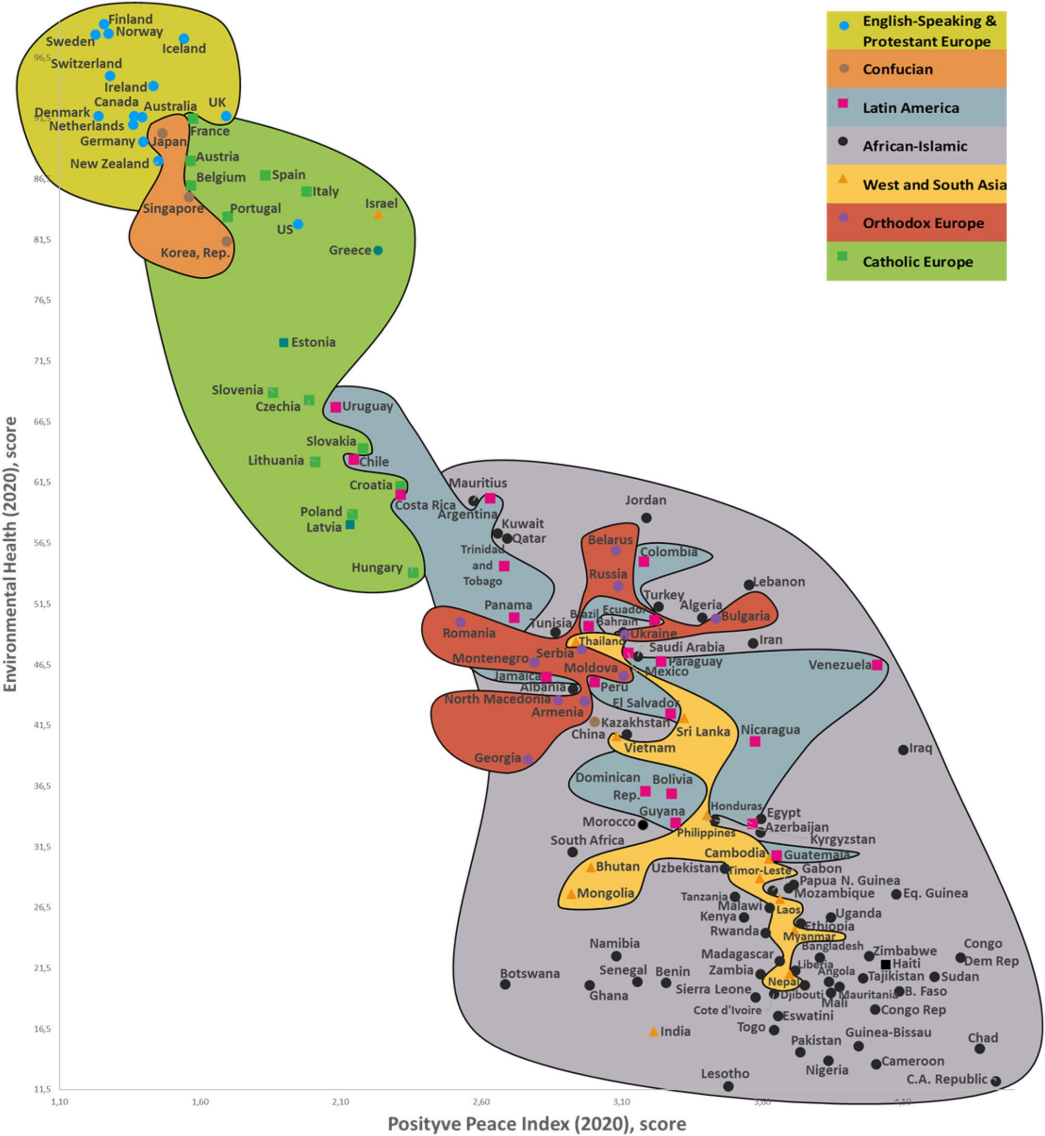
Environmental health and positive peace map.

Twelve CSS Models were developed to confirm the first hypothesis. Tables S5-S8 show the descriptive statistics of 12 CSS Models (Supplementary section 3). These Models, which are formal representations of the CSS Maps, show that when a country’s success increases by 1%, its 12 indicators related to the three dimensions of sustainability improve by 0.85% on average (Supplementary Table S8). Furthermore, Figs. 2, 3, 4 and 5 show that an increase in a country’s success is accompanied by an improvement in its indicators related to the three dimensions of sustainability, thus visually confirming the first hypothesis. These figures clearly show that the three dimensions of sustainability improve and the specific country’s success grows in parallel.

On average, 80.8% of the dispersions for the indicators of the three dimensions of sustainability are explained by our CSS Models, while for national success the same indicator equals 98.2% (Supplementary Table S8). The median correlation of all 33 traditional indicators included in the analysis and applied to 173 countries is moderate (Supplementary Table S3). Supplementary Table S4 shows the correlation coefficient matrix of the 17 success criteria for each of the 173 countries analyzed in this study. Figure 1 shows the strong median correlation between the 173 countries. Thus, the count of included countries, or changes in their systems of traditional key indicators, have little impact on the comparative values of country sustainability and success. Furthermore, the choice of traditional key indicators and their system does not greatly affect the country groupings within the seven country clusters analyzed in this research. The second hypothesis has been substantiated by the analysis results related to the CSS Models, as well as the correlations identified between the 173 countries and 33 indicators (Supplementary Tables S3 and S4, Fig. 1). Figures 2, 3, 4 and 5 that show the specific country groupings within the seven clusters discussed in this paper also offer a clear, visual validation of the first hypothesis.

The CSS Maps of the World developed as part of this study visualize two dimensions. One dimension is success (x-axis), and the other is the three pillars of sustainability (y-axis). All the above indicators, their values, and significance are listed in Supplementary Tables S1 and S2. The indicators of the three dimensions of sustainability analyzed in this research and represented along the y-axis include environmental (environmental performance index, ecological footprint, environmental health, air quality, PM_2.5_ exposure, climate change), social (healthy life expectancy, life expectancy at birth, death rates from air pollution, happiness index, positive peace index), and economic (the gross national income per capita) sustainability indicators. The countries in the CSS Maps are grouped using the same eight clusters (Catholic Europe, Orthodox Europe, Protestant Europe, Confucian, West and South Asia, African-Islamic, English-speaking, and Latin America) as those used in the Inglehart–Welzel 2020 Cultural Map of the World^21^. The English-speaking and Protestant European clusters were combined into a single cluster due to their religious affinity, their shared and related history, similar development levels, and cultural interactions. The similarities between the Protestant European and English-speaking clusters have been noted in many studies^34^. The Inglehart–Welzel 2020 Cultural Map of the World covers many economic, institutional, psychological, and technological variables with strong discernible correlations among them^35^. The CSS Maps’ indicators of national success can be defined as a large pool of variables within the system of criteria such as political, environmental, macroeconomic, values-based, human developmental and well-being, and quality of life. This study compares 173 countries included in the CSS Maps and Models with the 99–103 countries in the Inglehart–Welzel 2020 Cultural Map of the World, in order to draw statistical parallels.

Our research (Supplementary Sections 1, 4, and 5) analyzes conceptually the following three dimensions of sustainable national development (with the research findings compared with similar studies from around the world) quantitatively (CSS Models) and visually (CSS maps). Our CSS Maps are based on several distinctive traditional indicators (marked in *italics*) from each indicator group of the three dimensions of sustainability (y-axis) as they relate to country success (x-axis):

1. Environmental sustainability indicators (Fig. 2): *environmental performance index (EPI) (2020)*; ecological footprint per capita (2018); environmental health (2020); *air quality (2020)*; PM_2.5_ exposure (2020); *climate change (2020).*
2. Social sustainability indicators (Fig. 3): *life expectancy at birth, total (2019)*; healthy life expectancy (2019); death rates from air pollution (2019); *happiness index (2020); positive peace index (PPI) (2020).*
3. Economic sustainability indicator (Fig. 4): *the gross national income per capita (2020).*

The Country Success and Environment Sustainability World Maps visually demonstrate the association between country success (x-axis) and environmental sustainability (y-axis) for 99 countries (Fig. 2). Country success strongly and positively correlates with six environmental sustainability indicators (Supplementary Table S3). The CSS Map Environmental Sustainability Models explain 76.3% on average of the dispersions among the environmental sustainability indicators. An increase of 1% in a country’s success is accompanied by an increase of 0.84% on average in its environmental sustainability indicators (Supplementary Table S8).

The Country Success and Social Sustainability World Maps graphically show the relationship between the priority of country success (x-axis) and social sustainability (y-axis) for 102–103 countries (Fig. 3). Country success indicators strongly correlate with five social sustainability indicators (Supplementary Table S3). The CSS Map Social Sustainability Models explain on average 83.4% of the dispersions among the social sustainability indicators. An increase of 1% in a country’s success is accompanied by an improvement of 0.39% on absolute average in its social sustainability indicators (Supplementary Table S8).

The CSS Gross National Income Per Capita Map visually depicts the association between country success (x-axis) and economic sustainability—the gross national income per capita (y-axis) for 102 countries (Fig. 4). The CSS map plot shows that as the country’s success increases, the gross national income per capita rises (r_1 26_ = 0.930; p < 0.01) (Supplementary Table S3). The CSS Gross National Income per Capita Model explains 94.5% of the income indicator dispersions. When a country’s success increases by 1%, its income indicator increases by 3.309% (Supplementary Table S8).

Based on multiple criteria and statistical analysis across 173 countries, our results suggest an interconnection between a country’s 12 sustainability criteria. For example, the Environmental Health and Positive Peace Map visually demonstrate the association between country positive peace (x-axis) and environmental health (y-axis) for 150 countries (Fig. 5).

Multi-variant design and multiple criteria analysis of national policy alternatives are performed, and rational decisions are identified in stage 10. Rational country success and sustainability can be ensured by analyzing multiple alternatives and their detailed indicators, taking into account the existing state of the micro, meso, and macro environment. Several worldwide best practice examples^14,36–38^ of ways to identify rational activities, policy, and strategy are briefly analyzed in Supplementary Sections 1, 4, and 5.

## Discussion and conclusions

The 2030 Agenda for Sustainable Development highlights Sustainable Development Goals and the interlinked, integrated, and balanced environmental, social, and economic dimensions of sustainable development. The idea that national indicators of success and environmental, social, and economic sustainability move in tandem^39–43^ has been debated for decades. This phenomenon reflects one of the big picture trends of our modern world. The focus of our article is the Country Success and Sustainability (CSS) Maps and Models of the World, which show how the indicators of the three dimensions of sustainable development improve as a country becomes more successful, and declines in these indicators lead to decreases in the country’s success. Similar findings presented in a wide range of studies highlight the need to continuously improve the three dimensions of sustainability (Supplementary Sections 1, 4 and 5).

Figure 1 shows the moderate and strong overall median of all correlation coefficients between the 173 countries and 33 indicators. The same figure presents the moderate and strong correlations median between the nine CSS Map dimensions (the x-axis and y-axis), and the survival versus self-expression values and the traditional versus secular–rational values.

According to the CSS Models, whenever there is a 1% increase in a country’s success, this is accompanied by an improvement of on average 0.85% in its 12 indicators related to the three dimensions of sustainability. Meanwhile all 17 of the country success variables used in the CSS Models explain 98.2% of the dispersion of the country success variable and 80.8% on average of the dispersion of the three dimensions of sustainability (Supplementary Table S8).

The seven cultural clusters reflect the stable national success and sustainability similarities because similar cultures tend to move together in basically the same cultural, success, and sustainability direction. Higher success indicators are accompanied by higher sustainability indicators, which is also shown by the strong correlations obtained in our study (Supplementary Tables S1–S3). Some country’s success and sustainability indicators are cointegrated, moving together, and therefore cannot be changed in isolation. Sobel and Coyne^44^ and Coyne and Sobel^45^ confirmed these findings.

The human development index (β_av5_ = 0.506), GDP per capita (β_av1_ = 0.399), GDP per capita in PPP (β_av2_ = 0.251), and the corruption perceptions index (β_av4_ = 0.148) were the success variables with the strongest impact on 12 sustainability indicators given an increase of one unit in the independent variable. Human development index (HDI) has the highest values of standardized coefficients’ beta and effect size in eight CSS Models out of 12. For example, Model 3 indicates that HDI has a positive relationship (β_5_ = 0.719) with the dependent variable (environmental health) and is statistically significant at α = p < 0.001 (Supplementary Table S7). The HDI reaches a high correlation of 89.9%, (Supplementary Table S3) confirming that HDI and environmental health are highly and positively correlated. In second place in CSS Models, economic indicators have the most significant standardized coefficients beta and effect size values (Supplementary Table S7). Economic factors have been shown to make the biggest impact on this dispersion. This is hardly surprising, given that a number of studies indicate a sizeable continuum of influence attributable to GDP.

Model 3 has the explanatory power of 91.5% for environmental health, while Model 4 explains 83.4% of the air quality dispersion (Supplementary Table S8). Independent variables, such as the human development index (β_54_ = 0.586) and GDP per capita (β_14_ = 0.548), make a significant impact on air quality (Model 4), a dependent variable, for the 173 countries in question (Supplementary Table S7). The influence of the remaining independent variables on this variable is present but much lower. Meanwhile, the entire pool of variables used in the CSS Models explains 53.5–97.7% of the dispersion (Supplementary Table S8). The study revealed that we could use CSS Models and the INVAR method^46^ (Supplementary Section 2 and Fig. S1) in an integrated manner for sustainability policy strategies development and analysis. The CSS Models show that relationships exist between 12 sustainability and 17 success parameters for the 173 countries, and that they are statistically significant. This means that 12 sustainability parameters depend on 17 indicators of the country's success, and that they can be used for the prediction of sustainability in 12 parameters. For example, in order to improve a country’s sustainability indicators, it is expedient for it to aim for a higher human development index. As this human development index includes income, education, and life expectancy indices, these indicators must be improved, and as a result, the country's sustainability indicators will improve.

The multiple criteria analysis, statistical calculations, and CSS Maps and Models have been used to derive practical conclusions and recommendations, including the finding that the growing success of a country also means higher sustainability indicators. This research sheds more light on the way a country’s response to the sustainability challenges depends on its existing success indicators and macro level context. Specific policies, therefore, should be set based on this information. Some countries, however, fail to do this in a systematic manner. Another important point is that some areas remain underexplored, including the endorsement of human development, a stronger assurance of gender equality, the reduction or elimination of corruption, and the improvement of social progress, education, and happiness.

Most countries need immediate action and systemic policies to ensure more effective control and to improve sustainability with the best global practices in mind. Countries aiming to improve their success effectively can use these research findings as practical guidelines and a scorecard leading toward a sustainable future.

### Practical applications and implications

The decision matrix shown in Supplementary Table S9 can help determine the most effective ways to improve the sustainability indicators. This is done by analysing 12 CSS Models and their 17 dependent variables. Hence, this decision matrix comprises twelve equivalent segments, one segment for one specific CSS Model to be analysed. We need to establish the cumulative effect size (Q_j_, P_j_, N_j_) of the 17 country success indicators on attempts to improve 12 sustainability indicators at once. The first segment describes the CSS Environmental Performance Index Model, the first of the models, and the last segment (Segment 12) describes the CSS Positive Peace Index Model. All alternatives are assessed against eight criteria: (1) Pearson’s correlation coefficient (the absolute value |r| of this coefficient is considered), (2) the coefficient of determination (R^2^), (3) the standardized beta coefficient (β), (4) standard deviation, (5) p values (probability level), (6) the research context, (7) practical significance, and (8) indicators with low sustainability values. The aim is to find out which of the 17 country success indicators has the biggest cumulative effect size in order to improve 12 sustainability indicators at once. At first sight HDI seems to make the biggest impact with its β values the biggest in 8 out of 12 CSS Models. Yet Supplementary Section 3 highlights the fact that when they analyse factors with the biggest effect size, researchers, in addition to the research context and the degree of an effect size, need to look at its practical significance, as well as other factors. No sure and simple answer is available and the decision matrix presented in Supplementary Table S9 must, therefore, be analysed using the effect size metrics and INVAR method^46^.

Our analysis of the best global practice (Supplementary Sections 2 and 3) shows that the weights of measures of effect size (q_j_) and the weights of dependent variables (Q_j_) have to be determined for the decision matrix (Supplementary Table S9). A range of objective, subjective and integrated weighting methods can be used for that end. When experts determine weights (q_j_ and Q_j_), they should consider the research context, practical significance and other factors. Once the cumulative effect size (Q_j_, P_j_, N_j_) of 17 country success indicators related to the improvement of 12 sustainability indicators are established, we will further analyse the possibilities to improve sustainability indicators. For instance, the following latest global trends towards sustainability merit further in-depth analysis: hybrid ways of working; more climate change disclosures; sustainability becomes the “new normal”; the integrated value chain of CO_2_ capture, utilisation, and storage; the move from net-zero to climate positive; innovation in nature-based solutions; testing net zero pledges given by companies; the growing role of regulations globally; legal requirements for companies to disclose their climate risks; the practice to penalise lack of climate action; the input of financial institutions in the race to net-zero; and pairing mitigation with adaptation (Supplementary Section 6).

The big picture of the CSS Maps’ research and the related implications are presented in this article. Yet, extensive studies in various fields are needed to validate the results of this research. For this reason, a set of specific criteria with specific weights and certain period-reliant factors are examined in this investigation. Future research will need a focus on more variables with different weights to assess other contexts and periods. These research findings will not only be included in upcoming studies, but will also cover other research areas to validate the results of this multiple criteria analysis by means of CSS Maps and the INVAR method^46^. As part of the integrated multiple criteria analysis examining a range of objects from various areas, this additional analysis will focus on national culture indicators (i.e., secular-rational values vs. traditional values, and self-expression values vs. survival values). In this case, the INVAR method^46^ could be used, by means of multiple criteria analysis, to examine various alternatives in three dimensions of sustainability, such as environment (environmental performance index, ecological footprint, environmental health, air quality, PM_2.5_ exposure, climate change, happiness index, positive peace index), social (healthy life expectancy, life expectancy at birth, death rates from air pollution), and economic (the gross national income per capita). These then require assessment against many different criteria in relation to the local context. Established combinations of rational alternatives make it possible to ensure appropriate response to the ever-changing situation in the real world. In this context, our research findings are comparable with similar findings presented by the authors mentioned in Supplementary Sections 1, 4, and 5. Like the CSS Maps, various aspects of the three dimensions of sustainability can also be mapped, such as people, economy, poverty and inequality, global links, and others. Naturally, this study has some weaknesses and limitations, and thus needs certain improvements. We present the aspects that need further consideration in this field in Supplementary Section 7.

## Method

Our study method includes the following stages: (1) framing the investigation problem, (2) examining the literature, (3) developing and verifying two hypotheses, (4) collecting data, (5) the multiple criteria examination of 173 countries by means of the Degree of Project Utility and Investment Value Assessments (INVAR) method, (6) calculating correlations between 33 indicators and the success of 173 countries, (7) building 12 regression models, (8) compiling eight Maps (of which seven are CSS Maps) visualizing national success and sustainability, (9) spatial perspective analysis, and (10) performing integrated linear regression, multi-variant design and multiple criteria analysis of national policy alternatives, in order to identify rational decisions.

This research is a quantitative study to examine the way national success affects 12 indicators of the three dimensions of sustainability in 173 countries, and uses the data from 2020, or the latest available.

As investigation methods, our CSS Maps and Models can make it easier to study interdependencies between country success and sustainability. Supplementary Section 1, 4, and 5 presents our literature analysis which is carried out to gain deeper insights into our CSS Maps and Models, and to better understand their components in the worldwide research context.

The following two core hypotheses have been proposed and verified for this research:

- Hypothesis 1—The increasing success of a country is generally accompanied by increasing values for the three dimensions of sustainability indicators, and declines in these indicators lead to decreases in the country’s success. Improving some sustainability indicators tends to improve other sustainability indicators.
- Hypothesis 2—Changes in the number of countries and their traditional key indicators system do not make a very significant difference to the relative national sustainability and success values. Likewise, the boundaries of the seven country clusters discussed in this research do not excessively depend on specific traditional key systems of indicators used in their analysis.

Along with different sets of national 17 success (Supplementary Table S1) and 12 sustainability (Supplementary Table S2) indicators, the INVAR method^46^ (Supplementary Section 2 and Fig. S1) was used to measure and map the success of the 173 countries selected as the focus for this research. The traditional statistical indicator systems defining country success and the three dimensions of sustainability are based on studies from various countries analyzed and combined. The INVAR method calculates an integrated criterion characterizing the overall success of the countries. This integrated criterion is directly proportional to the relative effect the values and weights of the given criteria make on the country’s success. The multiple-criteria INVAR analysis method has been applied to various countries, including Asian nations^47^, ex-Soviet states^48^, and a group of 169 countries^49^.

This research used data from the framework of variables taken from various databases and websites, including Transparency International, Global Data, Eurostat-OECD, the World Bank, Knoema, the World Health Organization, Global Finance, Freedom House, Heritage, the Global Footprint Network, Socioeconomic Data and Applications Center, Our World in Data, Climate Change Knowledge Portal (World Bank Group), and The Institute for Economics and Peace, as well as global and national statistics and publications. All 173 countries analyzed in this article are listed in matrices, along with their 17 detailed success (Supplementary Table S1) and 12 sustainability (Supplementary Table S2) indicators (systems of indicators, their numbering, values, and weights). The INVAR method^46^ was applied to perform multiple criteria analysis of the 173 countries, and the results are presented in Supplementary Table S1 and Figs. 2, 3, 4 and 5. We use equal and different weights of 17 indicators to calculate the deviation of priorities for the 173 countries, which stands at 5.34% (Supplementary Section 2 and Fig. S2).

Along with different sets of 12 national sustainability and 17 success indicators, the INVAR method^46^ was used to measure and map the success of the 173 countries selected as the focus of this research. The traditional statistical indicator systems defining country success and the three dimensions of sustainability are based on studies from various countries analyzed and combined. The INVAR method calculates an integrated criterion characterizing the overall success of the countries. This integrated criterion is directly proportional to the relative effect the values and weights of the given criteria make on the country’s success.

Supplementary Table S3 shows the correlations between all measures determined by analyzing 173 countries. Supplementary Table S4 reveals the correlation coefficient matrix of the 17 success criteria for each of the 173 countries analyzed in this survey.

Along the vertical axis y we analyze seven sustainability indicators, and along the horizontal axis x we analyze the success and priority indicators (9 CSS Map dimensions). The median correlation between the survival versus self-expression values and the nine CSS Map dimensions (the x-axis and y-axis) is moderate, whereas the median correlation between the traditional versus secular–rational values and the nine CSS Map dimensions is strong (Fig. 1).

Tables S5-S8 show the descriptive statistics of 12 CSS Models (Supplementary Section 3). Supplementary Table S8 shows the extent to which a 1% increase or decrease in success of country’s features can push sustainability indicators up or down, expressed as a percentage. Supplementary Table S8 also shows the degree to which the percentage changes of success or the values of country’s features explain or fail to explain the dispersion of sustainability indicators. These CSS Models (Supplementary Section 3) show that when a country’s success increases by 1%, its 12 indicators related to the three dimensions of sustainability improve by on average 0.85% (Supplementary Table S8). Furthermore, the 17 variables of country success used in the CSS Models explain 80.8% on average of the dispersion of the three dimensions of sustainability and 98.2% of the dispersion of the country success variable (Supplementary Table S8).

An increase of 1% in a country’s success is accompanied by a 0.39% average increase in its social and environmental (0.84% on average) sustainability indicators (Supplementary Table S8). On average, the CSS Sustainability Models explain 76.3% of the dispersions among the environmental sustainability indicators, 83.4% of the dispersions among the social sustainability indicators, and 94.5% of the dispersion among economic (i.e. the gross national income per capita) sustainability indicators (Supplementary Table S8).

The study produced the eight Maps (of which seven are CSS Maps) of the World based on an analysis of 99–150 countries (the 2020 Inglehart–Welzel Cultural Map of the World focused on 103 analogical CSS Maps countries). The two dimensions of country success on the CSS Maps are represented in a system of 17 variables (Supplementary Table S1). When a country’s success grows, its performance related to the three dimensions of sustainable development increases as well, and the eight Maps (of which seven are CSS Maps) clearly illustrate this relationship (Figs. 2, 3, 4 and 5). The CSS Maps of the World developed as part of this study are described in Supplementary Section 5.

Studies from various countries and our research suggest that country success and their features (x-axis) and sustainability indicators (y-axis) are generally strongly interrelated, and move in the same direction over time. This means that successful countries also perform better on sustainability dimensions.

Stage 9 involved analysis of the spatial perspective research in place for explaining and predicting globally recognised physical, spatial, and human patterns in multiple ways. We apply 12 CSS Models, alternative design and multi-criteria analysis methods for spatial perspective analysis (Supplementary Section 4).

The following additional two research objectives were set: (1) to determine the impact of a country’s success factors on sustainability metrics, and (2) to offer stakeholders recommendations regarding the strategies for improving sustainability indicators. The ways to improve sustainability indicators are determined by analysing 17 dependent variables (the main paper section “Practical applications and implications”, Table S9). As previously mentioned, in stage 10, national policy options have been examined by means of integrated linear regression, multi-variant design and multiple criteria analysis to identify rational decisions. Analysis of multiple alternative options and their detailed indicators, with a consideration of the existing state of the micro, meso, and macro environment, can ensure rational country success and sustainability. Below, a brief analysis of several best global practice examples of ways to identify rational policy, activities, and strategy follows. The examples presented below suggest that multiple possible alternatives must be designed, assessed against a system of micro, meso and macro indicators, and the most effective options selected to make countries more sustainable. In Isham and Jackson’s^14^ opinion, materialistic lifestyles and values have been associated with adverse effects on human health as well as having detrimental effects on our planet. Therefore, activities and lifestyles should be identified that promote human well-being, yet which at the same time protect ecological security. Isham and Jackson^14^ identify optimal activities (arts and crafts, reading, sports, meditating) with high levels of human well-being and low environmental costs. It is important to estimate pollution impacts on health in order to come up with the right policies for better health outcomes. Yet, the task is challenging because economic activity can lead to worse pollution, but can also improve health outcomes in its own right^37^. Humidity, temperature, dispersal by the wind, and other environmental factors contribute to pollution levels. Certain fine particulates can stay in the atmosphere for days, and travel long distances to be inhaled in places far away from the source, even in other continents. Local conditions must be reflected in emissions-control policies, and the global flows of air pollutants must be taken into account^6^. The explanation for the phenomenon of demographic transition could be improved public health in developed countries which results in a move toward a slower life strategy^38^. Studies show that children from wealthier backgrounds undergo puberty later than those from poor socio-economic backgrounds. Early puberty can lead to a variety of health problems and a shorter life. By the early adult years, the effects of exposure to trauma, post-traumatic stress disorder, and other conditions can become apparent in the form of diseases related to aging^9^. Education is a very important factor in economic growth, and is also strongly related to health. In addition to health benefits, substantial increases in education, especially of women, and shrinking gender gaps have an important effect on the roles and status of women in society^36^.

The INVAR method, statistical analysis, and the CSS Maps and Models can help generate multiple policy recommendations for various stakeholders. The possibilities are as follows:

- To create alternatives for ways to develop country success and sustainability, by performing countries’ multiple criteria and statistical analysis and identifying decisions that would be rational;
- to perform quantitative and qualitative analysis of the existing data and to interpret it. The results obtained this way would prompt automatic recommendations designed for different stakeholders on ways to improve country sustainability.

## Supplementary Information

Supplementary Information 1.

Supplementary Tables.

## Data Availability

Summary data tables appear in the manuscript and in the Supplementary Materials. The authors can deliver the applied raw data used for obtaining the conclusions in this paper to others upon request. All data utilized for this research can be downloaded from freely accessible websites.
