# Supplementary material for: Analysis of the impact of success on three dimensions of sustainability in 173 countries
Source: Sci Rep. 2022 Aug 30;12:14719. doi: 10.1038/s41598-022-19131-6 (PMC9427782; doi:10.1038/s41598-022-19131-6)
Supplement: Supplementary file 1 — Supplementary Information 1. [file 41598_2022_19131_MOESM1_ESM.docx]

**Supplementary Materials for**

**Analysis of the impact of success on three dimensions of sustainability in 173 countries**

Arturas Kaklauskas*, Loreta Kaklauskiene

*Correspondence to: arturas.kaklauskas@vilniustech.lt

**This PDF file includes:**

Materials

Tables S1 to S9

Materials

**1. Interrelationships between 17 success and 12 sustainability indicators of 173 countries**

We have outlined below the best practices and a literature analysis to better examine the association between our CSS Maps and Models and their constituents in the global investigation context.

The cultural dimensions proposed by Hofstede reflect the stable national dissimilarities because all cultures tend to move together in basically the same cultural direction, even when they evolve^1^. Between 1970 and 2006 Western cultures, as studies suggest, usually had partial convergence of cultural dimensions, yet their paths virtually never crossed^2^. Socialization, legislation, and the education system are practices determined by a society’s value system^3^.

In Stiglitz's^4^ opinion, the higher per capita incomes go together with higher national social indicators. Yet, the correlation is not perfect: Some countries and provinces (Costa Rica, Kerala, and Sri Lanka among them) have managed to achieve far better social indicators than would be expected from countries with their per capita income, by introducing active social policies to support their poor^4^. Wlezien^5^ states that spending preferences for various categories deemed to be related to social needs, such as welfare, education, health, and even the environment, move in tandem in the long run. Stimson et al.^6^ and Wood and Andersson^7^ conclude that the public has a very general preference for overall government activity, rather than specific preferences for discrete policy areas. With this in mind, the preferences noted in various spheres are indicators reflecting a single general preference for government action^6,7^.

Gans-Morse and Nichter^8^ believe that political and economic liberty tend to move in tandem over time.

Herzer and Strulik^9^ look at retrospective data on church attendance rates in a group of countries for the period between 1930 and 1990 to examine the relationship between income and religiosity extending over long time. Herzer and Strulik^9^ show a negative long-term relationship existing between the level of income (in GDP per capita terms) and the level of religiosity (in church attendance terms). The causal relationship is bidirectional: higher incomes cause religiosity to decline and this decline, in turn, leads to higher incomes.

The research findings presented by Solt et al.^10^ support the relative power theory. This theory states that religiosity increases in societies with greater inequality, because wealthy people are then more attracted to religion and their power enables them to shape the beliefs and attitudes of less well-off people.

Habel and Grant^11^ analyse the way public support for more government services is linked to religiosity. The findings are consistent with Habel and Grant^11^ theory and suggest public opinion and religiosity respond to insecurity; the series move in tandem over time, are positively correlated, and show similar response to changes in GDP per capita.

Diamond and Morlino^12^ presented eight different dimensions of democratic quality (responsiveness, the rule of law, freedom, vertical accountability, horizontal accountability, quality, competition, and participation), emphasising that they all interact and reinforce one another thus becoming a system. Lower-quality democracy can be of different types and deficient in different qualities, yet all these dimensions are interlinked and tend to move together either on the path to better and deeper democracy or towards decay^12^.

Cointegration tests show that economic growth and government expenditure are expected to move in tandem over time^13^.

According to Chen and Zhang^14^, multiple correlations can explain the discovered tendency among markets located in the same geographic region to move together, since economic ties are a bigger driver of market co-movements than simply being close geographically.

Market practices and various literature resources suggest that interest rates related to different maturities generally move together over time^15^.

Rule-of-law dimensions generally move in tandem. It means the performance of rich countries is usually high in most dimensions^16^.

Falk and Hermle^17^ analysed factors contributing to preference differences associated with gender such as positive and negative reciprocity, the willingness to take risks, altruism, patience, and trust and studied 80,000 participants in a Global Preference Survey from 76 countries. They compared the data with national variables such as gender inequality indices and gross domestic product and observed that greater equal opportunities for women led to greater differences in preferences between women and men.

Mental illness seems to increase the probability of crippling health expenditures, because affected individuals often simultaneously suffer from chronic illnesses such as heart disease and diabetes. Mental illness also worsens economic outcomes for individuals, with their work productivity reduced due to greater fatigue and reduced concentration. The poor live in inadequate housing in run-down neighbourhoods, and because of that are more exposed to environmental stresses such as challenging sleep environments, temperature extremes, and pollution, which can cause mental illness. The study’s findings show that low-cost therapeutic interventions and cash support can benefit those suffering from mental illness in straitened circumstances of poverty. Another adverse effect of mental illness is that it may also hinder skill acquisition and education among young people and, being disproportionately prevalent among women, exacerbate gender inequalities. Children’s educational attainment and cognitive development may suffer when their parents are afflicted by mental illness, and poverty and mental illness can be transmitted across generations^18^.

Among environmental health risk factors, ambient fine particulate matter (PM_2.5_) is the leading one in the world^19^. PM_2.5_ pollution is associated with increased risk of death caused by cardiovascular and respiratory diseases^20^.

Although the likelihood for richer individuals within a specific location to be mentally ill is lower, the overall rates of mental illness in richer countries are similar to those in other countries. Aggregate economic growth alone, therefore, is not enough to reduce mental illness. Another factor harming mental health may be the spread of ICT technologies and social media, with teenagers a particularly vulnerable demographic. The ongoing pandemic with its disproportionate impact on the poor and its likely lasting impacts on their mental and economic well-being, makes the causal relationship between mental health and poverty even more relevant^18^.

Living in densely populated areas, known as urbanicity, is an increasing environmental challenge for mental health (depression symptoms, etc.)^21^.

Other things being equal, significant economic development tends to make people more trusting and tolerant. Then they turn their focus on participation in decision-making and self-expression. Yet, in addition to economic factors, this process is also influenced by nation-specific events and a given country’s leaders, thus the process is not deterministic, and only probabilistic forecasts are possible. Moreover, severe economic collapse can reverse modernisation’s changes. The events in Japan, Italy, Germany, and Spain during the Great Depression and the situation of most ex-Soviet states during the 1990s are cases in point^22^.

Most countries that will spend less on public education and social transfers in terms of a GDP share will very likely be troubled countries by 2050. The choice to keep the same or lower levels of social spending over the first half of the 21st century would translate into stagnating life expectancy and average real income and no shift towards democracy^23^.

Looking at data from 161 countries for the period between 1970 and 2013, Apergis^24^ adopts a panel causality methodological approach to investigate the hypothesis that education has a notable effect on democracy. The empirical analysis suggests education does benefit democracy and seems to be the key means of intervention in a location with unstable democracy where many still have no access to education^24^.

The peace index correlates with interpersonal forgiveness at a significant level, with more peaceful societies showing higher rates of forgiveness. The correlation between forgiveness and wellbeing at the country level was also significant, as was the correlation between interpersonal forgiveness and the democracy indicator at the society level^25^. Forgiveness scores were higher in those countries that had scored higher on democracy and human rights. The association between interpersonal forgiveness and the socioeconomic indicator was marginally significant, with individuals reporting higher forgiveness scores in more highly developed societies^25^. Karremans et al.^26^ believe some evidence shows that forgiveness practices decrease depression and anxiety, and improve wellbeing at the individual level.

Despite the fact that it is methodologically difficult to prove that population displacement and climate change are linked by a direct causal relationship, this connection is still evident in some areas. The focus of this indicator is on situations with the aim of isolating instances where the only factor contributing to migration decisions is climate change, specifically rising sea levels^27^. Results suggest a considerable heatwave exposure disproportion: even at the 1.5 °C warming level populations in low development countries are, in 2075, expected to suffer exposure greater than the exposure of populations in very high development countries at the warming level of 2 °C^28^. The findings presented by Prada^29^ show that migration and sustainable development goals are interrelated. Migration can have an effect on the achievement of sustainable development, but a country’s progress towards its sustainable development goals likewise makes an impact on migration^29^.

**2. The INVAR method and a comparison of the success and priorities of 173 countries calculated according to different weights**

In recent years, there has been substantial progress in the development and application of multiple-criteria decision-making (MCDM) methods. According to the various techniques studied (ELECTRE, the multi-attribute utility theory, PROMETHEE, data envelopment analysis, AHP, goal programming, TOPSIS, a combination of techniques), the perceived benefits of the INVAR technique are that: it has a comprehensible rationality; it is easy to apply in stages 1–5, and the results are obtained relatively quickly; it is very practical for using in stages 5–10; it analyses both quantitative and qualitative data and integrates weights; and it evaluates numerous dimensions. The main drawback of the INVAR technique is that compared to other methods, the calculation cycles in stages 6, 7, and 10 are lengthier and more complicated. The main innovations of the INVAR technique are presented in stages 5–10 (Fig. S1).

**Figure S1.**The INVAR method was used in the multiple criteria investigation of 173 nations.

The success and competitiveness of the 173 countries analyzed applying this INVAR technique depends directly and proportionately on a system of criteria that characterize them adequately, and on the weights and values of the indicators (Tables S1–S3). Experts create the system of criteria and calculate criteria values and initial weights. Politicians and other stakeholders can make adjustments to this information, so that it fits their purposes. When any available alternatives are assessed, the results, therefore, reflect in full the initial data provided jointly by experts and stakeholder groups.

After analysis of the countries being compared, the results are presented in a matrix, where the columns show *n* countries, and the rows show the comprehensive system of criteria that characterize these countries. The system of criteria includes criteria names, their measuring units, values and weights, and the labels that mark each criterion either as minimizing (a lower value is better, as in the case of the unemployment rate, the economic decline index, or the fragile state index) or maximizing (a higher value is better, as in the case of GDP per capita, the human development index, economic freedom, the democracy index, or government effectiveness). The success and competitiveness of the countries analyzed in this research can only be characterized using a system of indicators with various different dimensions. Some countries were excluded from this analysis because no official comparable data were available for them.

The INVAR method offers a fairly easy way to assess and then identify the most successful and competitive countries with the physical meaning of this calculation process clear. The same method was also used to create a generalized (reduced) indicator Q_j_ that depends directly and proportionately on the relative influence the weights q_i_ and values x_ij_ of the indicator being compared have on the end result. This study uses Stages 1–5 of the INVAR method (Fig. S1).

The success, Q_j,_ of a country a_j_ shows its performance related to sustainability and success indicators. The most successful country will always get the biggest value Q_max_. The success of all other countries a_j_ will always be lower than Q_max_. This means that their performance, related to sustainability and success, will be below that of the most successful country.

To assess the pluses and minuses of the performance achieved by these countries more effectively, and to compare them with the needs and goals of politicians and other stakeholder groups, each country’s degree of competitiveness has to be determined. This degree of competitiveness depends directly on the system of criteria that characterize the countries and the values and weights of the criteria. If one country has performed better on the corruption perceptions index, the human development index, the global gender gap, and with regard to economic freedom, another country has performed better on the fragile state index, government effectiveness, civil liberties, and wealth per adult, and if multiple criteria analysis determines identical success for both countries, the degree of the competitiveness of the two countries will also be identical. As a country’s success increases, so does its competitiveness, and vice versa. This means that all countries in question will have degrees of competitiveness between 0% (the worst result) and 100% (the best result). This will make it easier to visually assess the competitiveness of the countries.

Below we present a comparison of the priorities of the 173 countries calculated according to the equal and different weights. The priorities of the 173 countries examined in this chapter and depicted on the x-axis are directly and proportionately dependent on the system of relevant criteria describing the countries, as well as on the values and weights of the criteria. We used beta coefficients (β) for the determination of the 17 indicators' weights (Table S7). We detailed why the beta coefficients are equal to the weights of the country's success indicators (β_i_ = q_i_) in Supplementary Sections 2 and 3. The beta coefficients in CSS Models (Supplementary Section 3) and MCDA methods weights^30–32^, for example, have very similar interpretations and meanings.

In validating the second hypothesis, equal and different weights of the 17 indicators were used as the basis in the calculations for the comparison of the priorities of the 173 countries. It can be seen that the country priority values calculated according to the equal weights (Table S1) of the 17 indicators are not very different from the country priority values calculated according to the different weights (<http://iti4.vgtu.lt/SAVAS/daugkrit.aspx?sistemid=2153>). The deviation of the priorities for the 173 countries determined in our calculations using equal and different 17 criteria weights was 5.34% (Fig. S2).

**Figure S2.** The comparison of the priorities of 173 countries calculated using equal and different weights of 17 indicators.

**3. Country Success and Sustainability (CSS) Models**

Seventeen independent variables and 12 dependent variables were analyzed to build the 12 regression models dealing with the success and sustainability of countries. We used 12 dependent sustainability variables (environmental performance index, ecological footprint per capita, environmental health, air quality, PM_2.5_ exposure, climate change, the gross national income per capita, healthy life expectancy, life expectancy at birth, death rates from air pollution, happiness index, positive peace index). The 12 regression models were based on the 17 independent variables shown in Table S1. Tables S5-S8 show the descriptive statistics of 12 CSS Models (Supplementary Section 3).

Descriptive statistics of 12 CSS Models and Country Success Model are presented in Table S5. The minimum (lowest) and the maximum (highest) value of a variable in the set of values the variable in question can take shows the interval within which its values vary. The mean is the average value of the full set of values a variable can take, usually equal to the arithmetical average. The standard deviation shows the distribution of a variable’s values around the mean. Kurtosis shows whether or not most values are densely clustered around the average. Skewness shows whether or not values are symmetrically distributed around the mean. In case of kurtosis and skewness, the values between –3 and 3 for skewness and between –10 and 10 for kurtosis are deemed acceptable. If skewness is around 0 and kurtosis is around 3, the distribution of the values of the variable in question within the value set in question follows the law of normal distribution.

**Table S5.** Descriptive statistics of 12 CSS Models and Country Success Model.

**Table S6.** Results of goodness-of-fit testing of CSS models.

**Table S7.** Standardized coefficients' beta values of the dependent variables.

R**^2^** measures the accuracy of a CSS Model in predicting a country’s sustainability indicators. The model’s dependent sustainability variables represent the outcome. The closer a CSS Model’s R² is to 1, the more accurate the model. The coefficient of determination (R**^2^**) thus shows what share of the variation in the dependent sustainability variable can be predicted by examining the independent success variables. R**^2^** is used in the context of CSS Models designed to predict the success and sustainability countries will achieve in the future and to test two hypotheses.

Abelson’s paradox concerns the possibility of paradoxical relationships between the coefficient of determination (R²) effect size and its practical significance. The example for this paradox was obtained through baseball, by analysing the R² for skill level and batting average. Although batting average is considered to be one of the key indicators of success, the effect size was negligible^33^.

Effect size is a value used in statistics to measure how strong the relationship is between two variables or to calculate a sample-size estimate of that amount^34^. Effect sizes can be the regression coefficient in a regression, the correlation between two variables, the mean difference, or the risk of a specific event occurring^35^.

If the standard deviation is too high, it will render the measurement virtually meaningless^36^.

In a quantitative study, effect size is the primary finding. Though the p-value can tell you if there is an effect, it cannot tell you its size. Both substantive significance (the effect size) and statistical significance (the p-value) are key results in the reporting and interpretation of studies^37^.

Beta weights can be arranged according to rank to ascertain the ‘best’ predictor variable in multiple linear regression. Theoretically, the highest ranking variable is the one with the largest total effect, since beta is a measure of the total effect of the predictor variables. However, decisions should not be made based on beta weights alone, particularly when there are numerous predictors. Rank-ordered beta weights should only be used in this way if it is clear that no multicollinearity exists^38^ or if the model is specified flawlessly. In any other case, the beta weight should only be used as the first step for further research, as it does not necessarily predict the criterion variable^39^.

For instance, a predictor (such as Xp) may have a high absolute correlation with Y but a beta weight of zero if its shared explanatory ability is attributed to one or more other correlated predictors. There are cases where a predictor is very good, even though it has a near-zero beta weight^40^. Basing the interpretation strategy on the beta weight creates dependence on the context, as including or excluding even one predictor can alter the weights drastically – and the resulting interpretations as well^41^. Dunlap and Landis^42^ argue that in different situations where different combinations of predictors are used, regression weight is apt to change, since it is dependent on the other predictor variables included in the equation.

Just as interpreting beta weights alone is inaccurate, so is interpreting structure coefficients (r) alone. A variable may have zero correlation with Y, but a sizeable non-zero beta weight. This acts on R² by permitting the beta weights for other predictors to deviate further from zero than the boundary of their respective zero-order correlations with Y. This results in some of the other product terms within the equation becoming larger, and R² increasing^39^.

Alternatively, the predictor may be a suppressor that improves model R² not by directly predicting Y, but by indirectly predicting Y by eliminating extraneous variance from the other predictors. Beta weights and structure coefficients r are the two sets of coefficients that provide a more perceptive stereoscopic view of the dynamics of the data^39^. Interpretation may be improved by other results as well^43^.

This research includes five measures of effect size – the coefficient of determination (R²), Pearson’s correlation coefficient (r), a standardised beta coefficient (β), standard deviation, and p-values – as they can be calculated from almost any study designs, and their calculations are required for meta-analysis. These five measures were extracted from all studies where available. Additionally, users and experts should consider the research context, practical significance and indicators with low sustainability values as measures of effect size.

Durlak’s study^44^ was meant to provide guidelines for the selection, calculation and interpretation of effect size (ES). To this end, the ESs are first defined, and their indispensability to research is highlighted. This is followed by a discussion of the ESs typically used in group designs and correlational studies. The study concludes with a few effective resources that can be used to discern different types of effects and modify their calculation to suit the purpose and methods of the study^44^.

There is no direct relationship between the p-value and the effect size. A small p-value can be associated with a small, medium or large effect. There is also no direct relationship between the effect size and its practical or clinical significance. A lower effect size on one outcome can be more important than a higher effect size on another outcome, depending on the circumstances. What gets complicated is the almost infinite number of ways researchers can design their research and analyse their data, which may involve slightly modifying how the ESs are determined^44^.

The guidelines that Durlak^44^ developed provide advice on what effect sizes to use in research, and how to calculate and interpret them.

There are many different types of effect sizes known, and that can be converted to other types, so they are mathematically related. As a rule, researchers have more confidence in more rigorous studies, though the definition of rigour varies from field to field. Findings based on more objective measurement strategies are generally considered to be more reliable than those based on self-report alone. Researchers analyse various research situations where one research method is preferable to another. Some ESs that are based on more robust research methodology carry more weight than others in terms of scientific opinion^44^.

As concerns power analysis, many authors adhere to Cohen’s^45^ suggestion that a standardised mean difference (SMD) of 0.20 be considered a ‘small’ effect size, 0.5 – a ‘medium’ effect size, and 0.8 – a ‘large’ effect size. Regarding r, Cohen proposed 0.10, 0.30 and 0.50 to be considered small, medium and large in magnitude, respectively. Cohen proposed these values prudently, to be used as a general rule when there is no information of the field under analysis or former outcomes^46^. Furthermore, it is wrong to assume that ‘large’ effects are always more significant than ‘small’ or ‘medium’ effects. The magnitude of the effect is important, but so is its practical or clinical significance, which must be taken into account. Three guidelines are proposed for evaluating ESs in context. The first guideline is to examine the source, i.e., the quality of the research that produced the effect. This goes for both new findings and previous pertinent research. The second is to compare apples to apples, i.e., to compare similar research conditions, especially in terms of the type of outcome measure. The third guideline is to take the clinical or practical significance of the findings into account^44^.

We used the following guidelines for calculating, reporting and interpreting ESs^44^:

- Selecting the most appropriate type of effect for the study objective, design and outcome.
- Providing the basic data for the key variables.
- Reporting the effects of all outcomes, regardless of whether the findings that were obtained were statistically significant or not.
- Stating precisely how the effects were calculated by providing a specific reference or the algebraic equation that was used.
- Interpreting effects in the context of other studies: (a) estimating the effect size according to the context of the study and its practical or clinical value; (b) only using Cohen’s^45^ benchmarks where comparison with other relevant studies is not possible.

Compared to GDP per capita, the human development index affects the three dimensions of sustainability even more systematically, with the most significant effect in eight of the twelve CSS regression models. We see a similar situation when evaluating the average effect of the standardized beta coefficients (β) on 12 CSS Models of the three dimensions of sustainability. For these 12 CSS Models, the following **average** β values of independent variables (criteria weights) in absolute values were calculated: the human development index (β_av5_ = 0.506), GDP per capita (β_av1_ = 0.399), GDP per capita in PPP (β_av2_ = 0.251), the economic decline index (β_av11_ = 0.157), the corruption perceptions index (β_av4_ = 0.148), the fragile states index (β_av10_ = 0.146), the government effectiveness index (β_av12_ = 0.138), the democracy index (β_av8_ = 0.118), the ease of doing business rank (β_av3_ = 0.11), and so on have the greatest cumulative influence (Table S7). By means of the seemingly unrelated regressions and the feasible generalized least squares, Adrangi and Kerr^47^ analyze the period between 2000 and 2017 looking at panel data from BRICS countries to produce a regression model that shows the way SDGs are linked with GDP. This research by Adrangi and Kerr^47^ shows that targeting GDP may not lead to achieving overall SDGs even though, historically, GDP has been prioritized above many other indicators as a measure of economic health.

To compare the strength with which each individual independent success variable affects the dependent sustainability variables, a standardized beta coefficient is used. Its absolute value shows the intensity of the effect, with higher values corresponding to stronger effect.

In statistics, standardized (regression) coefficients, also called beta weights or beta coefficients, are the estimates produced after regression analysis; the underlying data is standardized for this analysis to make the variances of independent and dependent variables all equal to 1^48^. This author shows the number of standard deviations a dependent variable will change with each standard deviation increase related to the predictor variable. The standardized coefficients used in CSS Models are unitless. This study’s CSS Models revealed noteworthy results regarding the success variables that predict whether or not the sustainability indicators (policy alternatives) examined in the research would substantially impact the sustainability values. R**^2^** of these CSS Models is between 53.5% to 97.7% indicating they are quite strong (Table S8).

CSS Models reveal the independent success variables that are expressed in a variety of units make a bigger impact on the dependent sustainability variable. Standardized coefficients (or beta weights) also quantify the intensity of the effect one variable has on another, and thus they act as a general measure of effect size. In our research, this effect size has been highlighted with green shades (the effect above average) and pink shades (the effect below average) and its darkness in the table cells with independent success variables (Table S7). When determining colours, beta weights are measured in absolute values. The higher the beta weight of an independent variable (the bigger its effect on the dependent sustainability variable), the darker is the shade of green in the specific cell. In case of the pink cells, however, the lower the beta weight of an independent variable (when its effect on the dependent sustainability variable is minimal), the darker is the shade of pink in the specific cell. Average beta coefficients (beta weights) can also be used as criteria weights (Table S7).

Twelve regression models were developed in analyzing the dependency on country success of the 12 sustainability criteria of the three dimensions of sustainability. A linear regression model was used to do this. It was found that by using linear regression, changes in the country success values explain each dispersion of the values of the sustainability indicators used in the study by an average of 80.8% (Table S8). Linear regression was therefore chosen for the study, as this allows the sustainability indicators of the countries to be predicted with sufficient accuracy according to the values of the country's success indicators.

The CSS Models, a formal representation of the CSS Map, evaluate the links between 17 independent variables (Table S1) and 12 dependent variables (Table S2). In order to determine the way our particular indicators impact the dispersion of the independent variables, we used the linear multivariable regression method. Table S8 shows how countries' success and its factors influence sustainability indicators.

**Table S8**. How countries’ success and its factors influence sustainability indicators.

**4. Spatial perspective analysis**

According to Coenen et al.^49^, one should devote attention to two particular problems which approach sustainability from a spatial perspective (and which are interrelated): institutional commitment regarding processes of socio-technical development within given territorial areas, and a clear multi-level concept of the relevant socio-technical implications. Coenen et al.^49^ also address the topic of a diverse range of transition process options, which is related to a “natural” range of institutional conditions, networks, and strategies for involved actors and situations concerning spatially allocated resources.

Davies^50^ has analyzed globalization and interconnections from a spatial perspective. Globalization refers to the existing increasing global physical movement, interconnections, and interdependence of people, information, goods, organizations, and situations which connect people and locations at a global scale, in this way bringing about changes in the structures and organizations of individual locations and society as a whole. Whether it is information flow systems or physical mobility and goods transfer activity, there are indeed a number of communication channels, with differing speeds, capacities, and constraints, the results of which cause considerably differing effects on the overall interaction system^50^.

One common agenda for governments in low-income countries is to put economic development first and protection of the environment second; this can give rise to a substantial array of significant environmental problems. In such countries, economic development—which inevitably depends on the flourishing of heavy industry—calls for mass consumption of energy resources, and one result of this is ever-exacerbating air pollution problems^51^.

In countries with incomes in the lower-middle or upper-middle ranges, the primary task involves the transformation of extensive economic growth paradigms into intensive ones, with a solid priority attributed to production infrastructure which pollutes less. Under these circumstances, governments are expected to optimize their industrial structures, along with achieving greater energy efficiency, by promoting technological progress. In this way, they can accomplish the objective of more environmentally friendly production^52^. Also recommended is further urban green space planning, which should improve the intensity and the functionality factors of ecological environments. Along with the development of a robust system for monitoring the environment, these measures are acknowledged as practical policy suggestions for reducing air pollution^53^.

In the case of high-income countries, governments should make a point of vigorously promoting and advocating cleaner, environmentally friendly lifestyles and production patterns. However, residents of countries with higher incomes have greater demands as far as living standards are concerned—satisfaction of their needs often requires greater energy consumption^54^. This means that policymakers are required to encourage these residents to develop living habits that are better for the environment, and that are marked by lower consumption levels, if air quality is to be enhanced^54^.

Deng et al.^55^ highlight the concept of developing eco-cities at multiple spatial levels— including from a perspective of urban governance—as well as collective effort on the part of stakeholder groups within various sectors.

The landscape elements, such as social movements and relevant discourse, and the individual values and norms that continue to be espoused, are by no means equally influential in terms of spatial context. In the light of such a spatial focus, the space and time factors of urban transitions vary greatly on account of their societal and political roots^56^. Fastenrath and Braun^57^ have defined sustainability transitions as altered practices in a wider socio-spatial context, and they highlight the interaction between politico-institutional, socio-cultural, and economic structures.

Together, the different social, economic, environmental, and cultural–historical elements have stimulated a debate regarding spatial sustainability that has attracted considerable attention^58^.

The concept of spatial sustainability needs to be tackled in a dynamic and non-biased kind of way, with insights obtained from fields as varied as neoclassical economics, ecology, and social–political sciences all requiring the right kind of attention. Spatial or regional sustainability should be looked at in a context of “bioregions” as opposed to political regions. The indicators gained from such studies should provide information that could prove useful in the matter of making trade-offs which demand a balancing of economic efficiency, spatial equity, and environmental sustainability^59^.

We simply cannot, on a global scale, continue to use more resources than those that nature provides us with. For example, what are the chances that a country like Colombia would willingly cede its unused biocapacity to the Netherlands, so that its lifestyle can remain intact? What if we were preventing there being enough space for different views of the world? Or is the truth that we are on the verge of labeling anyone who consumes less than the global average as “poor and oppressed”; all regions with ecological credit as “underdeveloped”; and all countries with an ecological surplus as “colonized”? It is typical for countries to accuse other countries of allowing unsustainable conditions to develop. Wealthy countries point the finger at poorer ones, saying that their governments should do something about their high populations; meanwhile, the poor respond by highlighting the true extent of how much wealthy countries consume per capita. Escaping this vicious circle of blame requires a more complex approach when it comes to resolving issues relating to population, consumption, scale, and sustainability. Applying the multiple criteria analysis—with integrated preferences and settlement type attributions—could prove a good way of helping to promote a more cooperative approach which should, ultimately, prove far more promising than indulging in the “blame game”^60^.

Method Stage 9 included a detailed analysis of the spatial perspective research in place for explaining and predicting globally recognised physical, spatial, and human patterns in multiple ways. We apply 12 CSS Models, alternative design and multi-criteria analysis methods for spatial perspective analysis.

**5. Country Success and Sustainability (CSS) Maps of the World**

The CSS Maps shows 173 countries distributed based on the values found along the y-axis (sustainability pillars) and the x-axis (country success).

The 17 success variables of 173 countries taken from the pool modeled in the 12 CSS Models explained 89.5% of the dispersions related to the environmental performance index, 84.1% of life expectancy at birth, and 98.2% of country success variables. In addition, the CSS Models explain 83.4% of the dispersions related to air quality, 79.2% of the happiness index, 66.6% of the climate change, and 97.7% of the positive peace index variables (Table S8).

Each country mapped along the x-axis has its success and priority influenced by the system of indicators, as well as by their values and significance. We applied the first five stages of the INVAR technique^61^ to determine the priority and success of each country (Table S1).

Where national data is deficient, the countries selected for the current study are not displayed. Eight clusters used in the Inglehart–Welzel 2020 Cultural Map of the World, namely Catholic Europe; English-speaking and Protestant Europe; Orthodox Europe; Confucian; West, and South Asia; African-Islamic, and Latin America^62^ are also used in the CSS Maps. The criteria interaction, shown in the main paper Figs. 1–5 and Tables S1–S9, is statistically significant, implying that improvements in country success lead to better sustainability criteria. Each country cluster has a tight outline, and represents clear clustering. Country groupings in the clusters of West and South Asia (where Israel, Thailand, and Vietnam are in a cluster other than their own), African-Islamic (where Qatar, Malaysia, Turkey, Kazakhstan, and Saudi Arabia are in a cluster other than their own), and Latin America (where Guatemala and Venezuela are in a cluster other than their own) stand out to a greater extent. China from the Confucian cluster, and similarly, Luxembourg, Austria, and Bulgaria also sometimes do not fall in their cluster (Catholic Europe and Orthodox Europe). On average, among the 99–150 countries analyzed across eight maps (of which seven are CSS Maps). However, there are reasons (geographical proximity, tight economic and cultural ties, religious affinity, shared and related history, and similar development levels) why these countries are outside their clusters. Some examples are given below. In the World Values Survey wave 6 map (2010–2014)^63^, Lithuania, Estonia, and Latvia belonged to their own Baltic cluster. This Baltic cluster is no longer included in the latest Inglehart–Welzel World Cultural Map**^62^**. Estonia and Latvia are nearer to the Catholic Europe cluster and, therefore, fall into it, and are marked by a close to Catholic European countries symbol and the color (
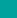
). The predominating religion in Greece is Greek Orthodoxy (followed by approximately 90% of the inhabitants), however, EU laws, politics, and culture make a large impact on it, and its geographical location is near Catholic countries. Thus, in maps it is marked by a close to Catholic European countries symbol and the color (
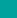
), and falls into the cluster of these countries. Traditionally, in Bosnia and Herzegovina, which is allocated to Orthodox Europe, more than half of the habitants are Muslims, thus, in CSS Maps, this country is marked by a transitional symbol and the color (
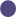
), and usually falls into Orthodox European and African-Islamic country clusters. In the Latin American country of Haiti, most of the inhabitants belong to the Afro-Haitian ethnic group. Thus, in CSS Maps it is marked with a transitional symbol and the color (
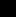
), as it is between a Latin American and African-Islamic country cluster, and usually falls into an African-Islamic country cluster. To illustrate the links between country success and the indicators in question of the three dimensions of sustainability, the main paper Figs. 2–4 show all countries (not only Inglehart [2020]) from the English-Speaking and Protestant Europe, Confucian, Orthodox Europe, and Catholic Europe clusters). In many cases, the countries in the African-Islamic, West and South Asia, and Latin American clusters are very close to each other in the main paper Figs. 2–4, and are difficult to represent graphically. Therefore, to separate these overlapping countries, and for visual clarity, data points for these maps are shown for Inglehart countries only. The main paper Fig. 5 represents the interrelationships between sustainability indicators for all countries under analysis.

The x-axis in CSS EPI, Air Quality, Climate Change and the Gross National Income per Capita Maps represents country success (as scores), and the same axis in CSS Life Expectancy at Birth, Happiness Index and Positive Peace Index Maps represents the priority of country success (on a 100-point scale, with 1 as the highest rank and 100 the lowest; the main paper Figs. 2–5).

**5.1. Environmental issues**

Since the turn of the millennium, loss of biodiversity, climate change, and pollution, as well as land degradation (such as the general degradation of ecosystems and deforestation) have been the most prominent of environmental issues^64^. Various means have been recommended to measure a country’s impact on the environment: the environmental performance index, the ecological footprint, environmental health, sustainable yield, ecological debt, air quality, PM_2.5_ exposure, and carrying capacity, to name a few. Countries’ performance impacts on worldwide ecosystems can be assessed by analyzing climate change, and other above indicators.

The group of environmental indicators under analysis includes environmental performance index (EPI), ecological footprint per capita, environmental health, air quality, PM_2.5_ exposure and climate change. The CSS EPI Map (the main paper Fig. 2a) visually depicts the relationship between country success (x-axis) and environmental performance index (EPI) (y-axis) for 99 countries. Country success shows a strong positive correlation with EPI (the relationship between these criteria is positive and statistically significant (r_1 20_ = .843; p < .01).

The CSS Air Quality Map (the main paper Fig. 2b) visually demonstrates the association between country success (x-axis) and air quality score (y-axis) for 99 countries. Country success shows strong positive correlation with air quality (r_1 23_ = .871; p < .01). Across the world, about seven million people die annually due to causes related to air pollution. WHO data shows that the air which 99% of the global population, or almost everyone on the planet, breathes is [polluted at levels](https://www.sciencedirect.com/topics/earth-and-planetary-sciences/pollutant-level) above the limits established by WHO guidelines^65^. Building on the result of the Dumitrescu-Hurlin panel causality analysis, Kongbuamai et al.^66^ suggest that economic growth and ecological footprint are linked by a bidirectional causal relationship. This is similar to the causality finding for Qatar in both the short and long term^67^, for the USA in both the short and long term^68^, for 11 newly industrialized countries^69^, for 14 Sub-Saharan countries in Africa^70^, as well as for MENA countries in both the short and long term^71^. Ecological footprint, energy consumption, and economic growth are linked by a bidirectional causality in ASEAN countries. The Dumitrescu-Hurlin panel causality analysis shows a bidirectional causal relationship between the ecological footprint, energy consumption, and economic growth^66^. Countries with a higher share of services in their economy have lower CO_2_ emissions^72^ and higher EPI scores^73^. Our research has also shown that environment indicators (environmental performance index, ecological footprint per capita, environmental health, air quality, PM_2.5_ exposure, climate change) moderately and strongly correlate with country success and their economic indicators, such as GDP per capita, GDP per capita in PPP, and the gross national income per capita.

The CSS Climate Change Map (the main paper Fig. 2c) graphically represents the relationship between country success (x-axis) and climate change score (y-axis) for 99 countries. Country success shows more than moderate positive correlation with climate change (r_1 25_ = .590; p < .01). Using a panel data methodological approach and an aggregate country sample, Apergis^74^ investigates the way personal well-being is negatively linked to per capita greenhouse gas emissions, and finds that these emissions make a significant impact on personal well-being. In Apergis’s^74^ opinion, reduced emissions imply lower economic growth, though the goal could be achieved by implementing more sustainable approaches to economic growth, for instance by reducing these greenhouse gases and solving social problems. Mikayilov et al.’s^75^ research has shown that in most of the countries scholars studied emissions increasing more slowly than GDP, therefore countries can take relevant measures to mitigate emissions level without harming economic development. The research results of Diffenbaugh and Burke^76^ show that many poor countries have been significantly harmed by the warming arising from wealthy countries’ energy consumption, thus increasing economic inequality between countries. Feedback, conservation, neutrality, and growth are four testable hypotheses that can be derived from the empirical literature discussing the causal relationship between economic growth and energy consumption^77–80^. The feedback hypothesis implies that energy consumption and economic growth are interrelated and complement each other in a way, thus suggesting a bidirectional causal relationship between them^77^. The conservation hypothesis implies that economic growth does not suffer adverse effects because of energy conservation policies, and suggests a causality running in a single direction from economic growth to energy consumption. The neutrality hypothesis posits that energy consumption constitutes a small fraction of overall output, and thus has a very limited impact on real GDP, or no impact at all. It implies that economic growth is not affected either by conservative or expansive energy consumption policies and, therefore, suggests no causal relationship between economic growth and energy consumption. Finally, the growth hypothesis implies both the direct and indirect impact of energy consumption (as a complement to capital and labor) on economic growth in the production process, and suggests a causal relationship from energy consumption to economic growth running in a single direction^81^. Climate and humans are linked, and one quantitative multidisciplinary empirical study has highlighted some important aspects of these links^82^. These authors have computed that conflict risk in Africa increased by ~11% due to global warming since 1980, current U.S. maize yields decreased by ~48% due to rising temperatures, and future warming is likely to slow annual global economic growth levels by ~0.28 percentage points. Our study results have indirectly confirmed the findings of the abovementioned studies, and have shown that climate change is related to the positive peace index (r_25_ _31_ = -.668; p < .01) and economic indicators (GDP per capita (r_25_ _3_ = .554; p < .01), GDP per capita in PPP (r_25_ _4_ = .551; p < .01, the gross national income per capita r_25_ _26_ = .563; p < .01), the economic decline index (r_25_ _13_ = -.473; p < .01), and wealth per adult (r_25_ _19_ = .266; p < .01) of 173 countries. The CSS Climate Change Map shows that country success is interdependent with progress in combating global climate change—with the higher success countries showing greater progress in combating global climate change.

Based on numerous studies, climate change is likely to push numbers of excess deaths up. Projected mortality impacts show significant spatial heterogeneity with colder and richer places affected less than hotter and poorer places^83^. Over recent decades, the trend of a warming climate has already contributed to mortality and morbidity increases in many regions around the globe^84^. The climate hazards, as has been explained by Watts et al.^27^, to which vulnerable populations are exposed, are more extreme. Indicators show that such groups suffered 157m heatwave exposure events in 2017, and, due to rising temperatures, lost more than 153bn hours of labor. Another negative effect is the eventuation of climatic conditions most conducive to the spread of dengue fever since 1950^27^. Our research has indirectly validated these findings, and has shown that climate change indicator are related to social indicators (healthy life expectancy (r_25 27_ = .603; p < .01) and life expectancy at birth (r_25 28_ = .604; p < .01).

The CSS Map of Environmental Sustainability Models explains on average 76.3% of the dispersions among the environmental sustainability indicators. An increase of 1% in a country’s success is accompanied by an improvement of 0.84% on average in its environmental sustainability indicators (Table S8).

# **5.2. Social sustainability**

Social sustainability focuses on the achievement of healthy living that is also satisfying for individuals and communities. To accomplish this way of life sustainably, social, material, and emotional needs should be provided for, and behaviors that result in conflict, emotional distress, and poor health should be avoided^85^. In their analysis of different countries, researchers^86,87^ found moderate and strong correlations between human emotional and physiological states and the diurnal cycle. Cheshmehzangi^88^ discussed social sustainability by analyzing health, peace through quality of public life, and other indicators. The group of health indicators includes healthy life expectancy, life expectancy at birth, total death rates from air pollution, and happiness index. The CSS Life Expectancy at Birth Map (the main paper Fig. 3a) visually shows the association between the priority of country success (x-axis) and life expectancy at birth (y-axis) for 103 countries. The main paper Fig. 3a shows that increasing country success priority is accompanied by increasing life expectancy at birth (r_2_ _28_ = -.730; p < .01).

The socioeconomic concept of the longevity dividend discussed by Scott^89^ means achieving productive and healthy aging through a positive correlation between the economy, health, and life expectancy. The fast economic growth of many middle-income countries has, therefore, very likely contributed to populations becoming healthier^90^. Acemoglu and Johnson^91^ show that health improvements contribute to income growth. Our investigation has confirmed the findings presented by these and other researchers. Our research has shown that increases in country success and improvements in their indicators, such as economic (GDP per capita, GDP per capita in PPP, the gross national income per capita), and the human development index, leads to increases in healthy life expectancy (r_1 27_ = .645; p < .01, r_3 27_ = .619; p < .01, r_4 27_ = .682; p < .01, r_26 27_ = .638; p < .01, r_7 27_ = .895; p < .01) and life expectancy at birth (r_1 28_ = .663; p < .01, r_3 28_ = .651; p < .01, r_4 28_ = .714; p < .01, r_26 28_ = .669; p < .01, r_7 28_ = .907; p < .01). A 1% increase in GDP per capita, GDP per capita in PPP, and the human development index is accompanied by a 0.127%, 0.157%, and 0.357% increase in life expectancy at birth, respectively. Changes in the values of GDP per capita, GDP per capita in PPP, and the human development index explain 42.4%, 51.0%, and 82.3%, respectively, of the dispersion of life expectancy at birth, in total. Our CSS Life Expectancy at Birth Map (the main paper Fig. 3a) also visually confirms that increases in country success correspond to increases in human life expectancy.

The data on the global burden of disease for 2010 shows that, around the world, 3.3 million premature deaths per year are caused by outdoor air pollution, predominantly related to PM_2.5_, and Asia suffers most. If nothing changes in terms of emissions, model projections indicate that the negative effect of outdoor air pollution on premature mortality could be double the current rate by 2050^92^. Among risk factors for human health, air pollution ranks fourth. In Europe, levels of air pollution are declining, yet it still represents a major economic and health burden^93^. Estimates show that air pollution, recognized as a serious risk factor for chronic non-infectious diseases, contributes to global mortality and morbidity more than all other known environmental risk factors put together^94^. Our research has revealed similar worldwide social sustainability trends. As the indicators of environmental health—air quality and PM_2.5_ exposure—improve, so do the health indicators analyzed in this research: Healthy life expectancy (r_27 22_ = .867; p < .01, r_27 23_ = .766; p < .01, r_27 24_ = .496; p < .01) and life expectancy at birth (r_28 22_ = .867; p < .01, r_28 23_ =.767; p < .01, r_28 24_ =.488; p < .01) are increasing, and death rates from air pollution (r_29 22_ = -.809; p < .01, r_29 23_ = -.807; p < .01, r_29 24_ = -.656; p < .01) are decreasing.

The CSS Happiness Index Map (the main paper Fig. 3b) visually demonstrates the relationship between the priority of country success (x-axis) and happiness index (y-axis) for 103 countries. Increasing country success (its priority) is accompanied by an increasing happiness index (r_2 30_ = -.731; p < .01). Both environment and genes influence most, or perhaps even all, human traits, including happiness. A set of genetic variants enables some fortunate people to feel happiness more easily. Environment and genes, however, are generally correlated suggesting there is “gene-environment interaction”: People may choose a certain environment influenced by their genes. Genes can also influence the way people interact with the world around them, and are affected by it. Findings from genetically informative designs should be used to create happiness-enhancing activities, social policies, interventions, and environments. Such actions would enable the full use of genetic potential and, at the same time, mitigate vulnerability and risk^95^. The correlation between national GDP and the share of population saying they feel at peace is 0.48 (p < .001)^95^. Having identified that some nations, despite lower income levels, scored fairly high on life satisfaction and health, Chapman et al.^96^ suggest further research is needed to uncover other, non-income-based factors that influence health and life satisfaction, such as familial factors or community connectedness. The findings by Li et al.^97^ suggest that the atmospheric conditions in Beijing affect emotions, and an increase of about 150 in the AQI (air quality index) in fine particulate matter PM_2.5_ triggers negative emotions. Compared with Chinese observers, those in the UK have a stronger negative response to severe air pollution. Social groups are also an important factor, with sensitivities of people related to subjective well-being when the AQI exceeds about 200, seemingly dependent on the group the people belong to^97^. Our analysis has confirmed that the happiness index has integrated relationships with the given factors, as noted by above researchers, and suggests that improvements in the economic sustainability (GDP per capita (r_3 30_ = .710; p < .01), GDP per capita in PPP (r_4 30_ = .724; p < .01), the gross national income per capita (r_26 30_ = .710; p < .01), environmental sustainability (environmental performance index r_20 30_ = .790; p < .01), ecological footprint per capita (r_21 30_ = .618; p < .01), environmental health (r_22 30_ = .812; p < .01), air quality (r_23 30_ = .752; p < .01), PM_2.5_ exposure (r_24 30_ = .572; p < .01), climate change (r_25 30_ = .611; p < .01)) and social sustainability (healthy life expectancy (r_27 30_ = .760; p < .01), life expectancy at birth, total (r_28 30_ = .739; p < .01), death rates from air pollution (r_29 30_ = -.695; p < .01) lead to better happiness index scores. The CSS Happiness Index Map (the main paper Fig. 3b) illustrates this finding, and shows that growing country success is accompanied by increases in the happiness index and vice versa.

The positive peace index was analyzed as an example. The CSS Positive Peace Index Map visually shows the association between the priority of country success (x-axis) and positive peace index (y-axis) for 102 countries (the main paper Fig. 3c). The CSS Positive Peace Index Map shows that increases in country success (its priority) lead to a better positive peace index ranking and vice versa (r_2 31_ = .902; p < .01). In our research, the positive peace index demonstrates a strong positive or negative correlation with the country’s development, governance, and business environment criteria, such as the ease of doing business ranking (r_5 31_ = .808; p < .01), the corruption perceptions index (r_6 31_ = -.926; p < .01), human development index (r_7 31_ = -.873; p < .01), the global gender gap (r_8 31_ = -.609; p < .01), government effectiveness (r_14 31_ = -.939; p < .01), and other country success indicators. The Global Peace Index^98^ and DiRienzo^99^ present similar findings. The Global Peace Index^98^ shows the correlations for the eight pillars of positive peace in high peace countries in 2019. The pillars are: acceptance of the rights of others (0.68), well-functioning government (0.66), low levels of corruption (0.74), good relations with neighbors (0.41), a sound business environment (0.69), free flow of information (0.62), equitable distribution of resources (0.58), and high levels of human capital (0.69). Correlation coefficients above r = .4 are considered to have strong significance, whereas those greater than r = .3 are considered to have moderate significance. Countries transition to high positive peace in a gradual process and the correlations become stronger along the way. This suggests that all pillars are important, and in terms of internal peace better performance is likely to result in lower external conflict, or at least correlate with it^98^. DiRienzo^99^ suggests there is a causal effect between the share of women in government bodies and peace. The focus on societal needs plays a role, as well as, indirectly, reduced corruption. The empirical evidence provided by DiRienzo^99^ supports the statistically stronger indirect effect of women in governmental authority positions on country peace, when the level of country corruption is accounted for.

Our study findings show that the climate change indicator and 2020 Inglehart–Welzel Cultural Map of the World^62^ dimensions (survival versus self-expression values, traditional versus secular–rational values correlate with the positive peace index (r_25 31_ = -.668; p < .01, r_32 31_ = -.810; p < .01, r_33 31_ = -.761; p < .01). This means that countries with high positive peace have lower impacts on climate change. The research outcomes obtained by Letendre et al.^100^ are related. Many researchers are interested in cross-national and geographic variation in the frequency of civil wars and internal armed conflicts. The theory proposed on this variation has mostly focused on the ways climate, competition for resources, cultural characteristics, and national wealth impact human behaviour^100^. These authors argue that their evidence shows that infectious diseases have a direct impact on internal armed conflicts, and an indirect effect on civil wars because they reduce national wealth. The authors also analyze the way conflicts further contribute to wealth reduction and the increased incidence of diseases^100^.

In our investigation, the positive peace index correlates strongly, and with statistical significance, with the country’s economic, business environment, and developmental indicators (GDP per capita r_31 3_ = -.836; p < .01), GDP per capita in PPP (r_31 4_ = -.839; p < .01), the gross national income per capita (r_31 26_ = -.847; p < .01), the corruption perceptions index (r_31 6_ = -.926; p < .01), the human development index (r_31 7_ = -.873; p < .01), the economic decline index (r_31 13_ = .828; p < .01), and the wealth per adult (r_31 19_ = -.457) of 173 countries. Bayar and Gavriletea^101^ presented comparable research results. These authors analyze panel data covering the period between 2008 and 2014 in order to look at the way economic growth, terrorism, and peace interact in 18 MENA countries. Their findings suggest the positive effect of a peaceful environment on economic growth, whereas the effect of terrorism is negative. The evidence presented by Bayar and Gavriletea^101^ also shows a bilateral causal relationship between terrorism and economic growth, and peace and economic growth.

The CSS Social Sustainability Models explain on average 83.4% of the dispersions among the social sustainability indicators. An increase of 1% in a country’s success is accompanied by an improvement of 0.39% on average in its social sustainability indicators (Table S8).

# **5.3. Economic sustainability**

Stiglitz^4^ notes that higher national social indicators are seen when per capita incomes are higher. The environmental dimension and the economic dimension of sustainability are linked in various ways, such as making the polluter pay by taxing an activity, rewarding stewardship by subsidizing activities with positive social or environmental impacts, or imposing legal limits on pollution by outlawing certain practices^102^. The literature often reflects the notion of compromises between different dimensions, for example between that of economic growth and environmental management, in an attempt to reconcile economic growth as a way to address ecological and social problems^103^. Per capita income was analyzed as an example in the group of economic sustainability indicators. The CSS Gross National Income per Capita Map visually demonstrates the association between the priority of country success (x-axis) and the gross national income per capita (y-axis) for 102 countries (the main paper Fig. 4). The CSS Map plot shows that as the country’s success increases, the gross national income per capita rises (r_1 26_ = .930; p < .01). The CSS Gross National Income per Capita Model explains 94.5% of the income indicator dispersions. When a country’s success increases by 1%, its income indicator increases by 3.309% (Table S8).

**6. Quantitative and qualitative recommendations for stakeholders**

The following supplementary study purposes were set: (1) to define the impact of a country's success indicators on sustainability factors and (2) to propose interested parties' recommendations concerning the strategies for improving sustainability metrics. In light of the study outcome, we discuss several strategies for improving sustainability values, particularly for low indicators (see Discussion and conclusions; Practical applications and implications).

The human development index (HDI) has the top values of beta coefficients (β) in eight CSS Models out of 12 (Table S7). The countries with the highest HDI scores were found to have fairly minor changes in their environmental performance, and all were ranked high in the overall HDI^104^. Lower HDI scores correspond to lower environmental performance^105^. This, then, means that higher HDI leads to better environmental health and the possible reason is education which helps people expand their planning horizon and they, therefore, become more aware of the need to assure future well-being. In second place in CSS Models, economic indicators have the most significant β. Gradually changing variables (socioeconomic development is an example) show considerable inertia. The size of stocks accumulated up to any point is always much bigger than one year’s gains or losses. The annual GDP of any country is only a fraction of its lifetime GDP. When variables change or accumulate gradually, we can only see substantial changes in the long run^106^. CSS Models show that success factors are significant predictors of which sustainability policies a country should prefer. For example, a few cases were observed where a country’s success indicators (economic freedom, wealth per adult) made no noticeable impact on the country’s sustainability.

For calculating the above 12 CSS Models, we used linear regression, which is the most commonly used form of regression analysis. Analogous CSS linear and non-linear models can be calculated for each country. By applying these CSS Models for each country and building on existing global best practices^107–111^, country-specific recommendations can be made.

The results are used to offer stakeholders recommendations regarding the best policy strategies for improving the sustainability values, especially for indicators with low values that are best suited to target countries. For example, a Strategy for sustainability management in the United Nations system, 2020–2030 (Phase I: Environmental sustainability in the area of management [“Sustainability Strategy I,” CEB/2019/1/Add.1]) and Phase II: Towards leadership in environmental and social sustainability - (“Sustainability Strategy II,” CEB/2021/2/Add.1) can be used for this purpose.

The next step is to establish the cumulative effect size (Q_j_, P_j_, N_j_) of the 17 country success indicators on attempts to improve 12 sustainability indicators at once. The ways to improve sustainability indicators are determined by analysing 17 dependent variables (the main paper Section “Practical applications and implications”, Table S9). Many researchers, for instance, Phillis et al.^112^, share the opinion that to improve an index with low sustainability usually takes less effort or costs less than to improve an index with high sustainability by the same magnitude.

**Table S9.** The decision matrix to determine the most rational country success indicators to improve 12 sustainability indicators at once.

**7. Weaknesses and limitations of the study**

Naturally, this study has some weaknesses and limitations, and thus needs certain improvements. The following list outlines the aspects that need further consideration in this field:

1. In total, 173 countries were analyzed in this research. By adding even more countries over a more extended time period of analysis, the accuracy of the CSS Maps and Models would improve, and the global situation would be better reflected. Yet, a large number of indicators in a system means that fewer countries will incorporate all of the relevant indicators.
2. All the indicators discussed in this study had the same weight equal to one. One of the future plans is to evaluate the significances of the indicators further in order to ensure more accurate CSS Maps and Models. For this purpose, an integrated approach will be taken with both objective and subjective methodologies applied, and the significances of the indicators will be recalculated.
3. This research applied a multiple linear regression model. To present more accurate descriptions of the existing situation, future CSS Maps and Models will be compiled by applying machine learning and [data mining](https://www.sciencedirect.com/topics/earth-and-planetary-sciences/data-mining) as well as robust, stepwise, nonlinear, and nonparametric regression methods.
4. This study looked at all indicators at a national level. Different regions in a single country can, however, be developed to different levels, and multicultural countries and cities also have diverse cultural ethnic communities. Therefore, indicators for different regions of a single country should be valued differently. For this reason, future CSS Maps and Models developed for specific multicultural countries are expected to be based on different systems of criteria with different values and significances.
5. The basis of the 2020 Inglehart–Welzel Cultural Map of the World is the European Values Survey and the World Values Survey, while the CSS Maps are based on statistical indicators. Yet, these maps correlate significantly with one another (Table S3). Therefore, in the future, the expectation is to examine the surveys performed at national levels and statistical indicators focusing on a broader scope, in order to establish the ways they are linked by dependencies.
6. Investigating other aspects of the three dimensions of sustainability is planned for the future, such as markets, global links, economy, people, inequality and poverty, and others.
7. In the future, other aspects of the INVAR method, not used in current study, will be applied with the aim to present a more comprehensive CSS big picture. An entire generation of more appropriate and more efficient recommendations applicable in CSS Maps and Models is expected as the outcome.

**References**

1. Minkov, M. & Hofstede, G.  The evolution of Hofstede’s doctrine. *Cross Cultural Management: An International Journal* **18**, 10–20 <https://doi.org/10.1108/13527601111104269> (2011).
2. Inglehart, R. F. Changing values among western publics from 1970 to 2006. *West European Politics* **31**, 130–146 <http://dx.doi.org/10.1080/01402380701834747> (2008).
3. Hofstede, G. H.  *Culture’s Consequences: Comparing Values, Behaviors, Institutions, And Organizations Across 31 Nations* (2nd ed.) (Thousand Oaks, California: Sage Publications, 2001).
4. Stiglitz, J. E. Participation and development: Perspectives from the comprehensive development paradigm. *Review of development economics* **6**(2), 163–182 <http://dx.doi.org/10.1111/1467-9361.00148> (2002).
5. Wlezien, C. Patterns of representation: Dynamics of public preferences and policy. *The Journal of Politics* **66**(1), 1–24 <http://dx.doi.org/10.1046/j.1468-2508.2004.00139.x> (2004).
6. Stimson, J. A., Mackuen, M. B. & Erikson, R. S. Dynamic Representation. *American Political Science Review* **89**(3), 543–65 <http://dx.doi.org/10.2307/2082973> (1995).
7. Wood, B. D. & Andersson A. H. The dynamics of senatorial representation, 1952–1991. *The Journal of Politics* **60**(3), 705–736 <http://dx.doi.org/10.2307/2647645> (1998).
8. Gans-Morse, J. & Nichter, S. Economic reforms and democracy: Evidence of a J-curve in Latin America. *Comparative Political Studies* **41**(10), 1398–1426 <http://dx.doi.org/10.1177/0010414007305811> (2008).
9. Herzer, D. & Strulik, H. Religiosity and income: A panel cointegration and causality analysis. *Applied Economics* **49**(30), 2922–2938 <http://dx.doi.org/10.1080/00036846.2016.1251562> (2017).
10. Solt, F., Habel, P. & Grant, J. T. Economic inequality, relative power, and religiosity. *Social Science Quarterly* **92**(2), 447–465 <http://dx.doi.org/10.1111/j.1540-6237.2011.00777.x> (2011).
11. Habel, P. & Grant, J. T. Demand for God and government: The dynamics of religion and public opinion. *Politics and Religion* **6**(2), 282–302 <http://dx.doi.org/10.1017/S1755048312000570> (2013).
12. Diamond, L. & Morlino, L. The quality of democracy: An overview. *Journal of democracy* **15**(4), 20–31 <https://doi.org/10.1353/jod.2004.0060> (2004).
13. Gokmenoglu, K. & Alptekin, V. RE-EXAMINATION OF WAGNER'S LAW FOR OECD COUNTRIES. *Annals of'Constantin Brancusi'University of Targu-Jiu. Economy Series* **1** (2013).
14. Chen, N. F. & Zhang, F. Correlations, trades and stock returns of the Pacific-Basin markets. *Pacific-Basin Finance Journal* **5**(5), 559–577 [http://dx.doi.org/10.1016/S0927-538X(9**7)**00022-X](http://dx.doi.org/10.1016/S0927-538X(97)00022-X) (1997).
15. Mallick, A. K. & Mishra, A. K. Interest rates forecasting and stress testing in India: a PCA-ARIMA approach. *Palgrave Communications* **5**(1), 1–17 <http://dx.doi.org/10.1057/s41599-019-0236-7> (2019).
16. Botero, J. C. & Ponce, A. Measuring the rule of law. Available at *SSRN 1966257* <http://dx.doi.org/10.2139/ssrn.1966257> (2011).
17. Falk, A. & Hermle, J. Relationship of gender differences in preferences to economic development and gender equality. *Science***362**(6412), eaas9899 <http://dx.doi.org/10.1126/science.aas9899> (2018).
18. Ridley, M., Rao, G., Schilbach, F. & Patel, V. Poverty, depression, and anxiety: Causal evidence and mechanisms. *Science* **370**(6522), eaay0214 <http://dx.doi.org/10.1126/science.aay0214> (2020).
19. McDuffie, E. E. *et al.* Source sector and fuel contributions to ambient PM2.5 and attributable mortality across multiple spatial scales. *Nat. Commun.* **12,**3594 <https://doi.org/10.1038/s41467-021-23853-y> (2021).
20. Pye, H. O., Ward-Caviness, C. K., Murphy, B. N., Appel, K. W. & Seltzer, K. M. Secondary organic aerosol association with cardiorespiratory disease mortality in the United States. *Nat. Commun.* **12**(1), 1–8 <http://dx.doi.org/10.1038/s41467-021-27484-1> (2021).
21. Xu, J. *et al*. Global urbanicity is associated with brain and behaviour in young people. *Nat. Hum. Behav.* **6,**279–293 <https://doi.org/10.1038/s41562-021-01204-7> (2022).
22. Inglehart, R. & Welzel, C. How development leads to democracy: What we know about modernization. *Foreign affairs* **88**(2), 33–48 (2009).
23. Piketty, T. Taxation with representation. *Science* **310**(5756), 1906–1907 <http://dx.doi.org/10.1126/science.1101319> (2005).
24. Apergis, N. Education and democracy: New evidence from 161 countries. *Economic Modelling* **71**, 59–67 <http://dx.doi.org/10.1016/j.econmod.2017.12.001> (2018).
25. Hanke, K. & Fischer, R. Socioeconomical and sociopolitical correlates of interpersonal forgiveness: A three-level meta-analysis of the Enright Forgiveness Inventory across 13 societies*. International Journal of Psychology* **48**(4), 514–526 <http://dx.doi.org/10.1080/00207594.2011.651086> (2013).
26. Karremans, J. C., Van Lange, P. A. M., Ouwerkerk, J. W. & Kluwer, E. S. When forgiving enhances psychological well-being: the role of interpersonal commitment. *Journal of personality and social psychology* **84**(5), 1011–1026 <http://dx.doi.org/10.1037/0022-3514.84.5.1011> (2003).
27. Watts, N. *et al*. The Lancet Countdown on health and climate change: from 25 years of inaction to a global transformation for public health. *The Lancet* **391**(10120), 581–630 [https://doi.org/10.1016/S0140-6736(1**7)**32464-9](https://doi.org/10.1016/S0140-6736(17)32464-9) (2018).
28. Russo, S. *et al*. Half a degree and rapid socioeconomic development matter for heatwave risk. *Nat. Commun.* **10**, 136 <https://doi.org/10.1038/s41467-018-08070-4> (2019).
29. Prada, E.-M. The relationship between Sustainable Development Goals and migration. An EU-28 perspective. *Journal of Social and Economic Statistics* **9**, 28–45 <http://dx.doi.org/10.2478/jses-2020-0004> (2020).
30. Choo, E. U., Schoner, B. & Wedley, W. C. Interpretation of criteria weights in multicriteria decision making. *Computers & Industrial Engineering* **37**, 527–541 <https://doi.org/10.1016/S0360-8352(00)00019-X> (1999).
31. Rohde, L. *et al.* Determining indoor environmental criteria weights through expert panels and surveys. *Building Research & Information* **48**, 415–428 https://doi.org/10.1080/09613218.2019.1655630 (2020).
32. Da Silva, F. F. *et al.* Elicitation of criteria weights for multicriteria models: Bibliometrics, typologies, characteristics and applications. *BJO&PM* **18**, 1–28 <https://doi.org/10.14488/BJOPM.2021.014> (2021).
33. Roseman, I. J. & Read, S. J. Psychologist at play: Robert P. Abelson’s life and contributions to psychological science. *Perspect Psychol Sci* **2**, 86–97 https://doi.org/10.1111/j.1745-6916.2007.00031.x (2007).
34. Kelley, K. & Preacher, K. J. On effect size. *Psychological Methods* **17**, 137–152 [https://doi.org/10.1037/a0028086](https://psycnet.apa.org/doi/10.1037/a0028086) (2012).
35. Wilkinson, L. & Task Force on Statistical Inference, American Psychological Association, Science Directorate. Statistical methods in psychology journals: Guidelines and explanations. *American Psychologist* **54**, 594–604 [https://doi.org/10.1037/0003-066X.54.8.594](https://psycnet.apa.org/doi/10.1037/0003-066X.54.8.594) (1999).
36. Funder, D. C. & Ozer, D. J. Evaluating effect size in psychological research: sense and nonsense. *Advances in Methods and Practices in Psychological Science* **2**, 156–168 [https://doi.org/10.1177/2515245919847202](https://psycnet.apa.org/doi/10.1177/2515245919847202) (2019).
37. Sullivan, G. M. & Feinn, R. Using effect size-or why the P value is not enough. *J Grad Med Educ* **4**, 279–282 https://doi.org/10.4300/jgme-d-12-00156.1 (2012).
38. Pedhazur, E. J. *Multiple regression in behavioral research: explanation and prediction* (Harcourt Brace College Publishers, 1997).
39. Courville, T. & Thompson, B. Use of structure coefficients in published multiple regression articles: β is not enough. *Educational and Psychological Measurement* **61**, 229–248 https://doi.org/10.1177/0013164401612006 (2001).
40. Thompson, B. & Borrello, G. M. The importance of structure coefficients in regression research. *Educational and Psychological Measurement* **45**, 203–209 https://doi.org/10.1177/001316448504500202 (1985).
41. Thompson, B. Five methodology errors in educational research: The pantheon of statistical significance and other faux pas. in *Advances in social science methodology* vol. 5, 23–86 (Stamford, CT: JAI, 1999).
42. Dunlap, W. P. & Landis, R. S. Interpretations of multiple regression borrowed from factor analysis and canonical correlation. *The Journal of General Psychology* **125**, 397–407 <https://doi.org/10.1080/00221309809595345> (1998).
43. Johnson, J. W. A heuristic method for estimating the relative weight of predictor variables in multiple regression. *Multivariate Behavioral Research* **35**, 1–19 https://doi.org/10.1207/s15327906mbr3501_1 (2000).
44. Durlak, J. A. How to select, calculate, and interpret effect sizes. *Journal of Pediatric Psychology* **34**, 917–928 https://doi.org/10.1093/jpepsy/jsp004 (2009).
45. Cohen, J. *Statistical power analysis for the behavioral sciences* (Routledge, 1988). <https://doi.org/10.4324/9780203771587>.
46. Volker, M. A. Reporting effect size estimates in school psychology research. *Psychol. Schs.* **43**, 653–672 <https://doi.org/10.1002/PITS.20176> (2006).
47. Adrangi, B. & Kerr, L. Sustainable development indicators and their relationship to GDP: Evidence from emerging economies. *Sustainability* **14**, 658 <http://dx.doi.org/10.3390/su14020658> (2022).
48. Menard, S. Standardized Regression Coefficients. in *The SAGE encyclopedia of social science research methods* 1069–1070 (Sage Publications, Inc., 2004). <http://dx.doi.org/10.4135/9781412950589.n959>.
49. Coenen, L., Benneworth, P. & Truffer, B. Toward a spatial perspective on sustainability transitions. *Research Policy* **41**, 968–979 <https://doi.org/10.1016/j.respol.2012.02.014> (2012).
50. Davies, W. K. D. Globalization: A spatial perspective. in *Unifying Geography* 189–214 (Routledge, 2004).
51. Han, L., Zhou, W., Li, W. & Li, L. Impact of urbanization level on urban air quality: A case of fine particles (PM 2.5) in Chinese cities. *Environmental Pollution* **194**, 163–170 https://doi.org/10.1016/j.envpol.2014.07.022 (2014).
52. Wang, S. & Liu, X. China’s city-level energy-related CO 2 emissions: Spatiotemporal patterns and driving forces. *Applied Energy* **200**, 204–214 <https://doi.org/10.1016/j.apenergy.2017.05.085> (2017).
53. Wang, S., Fang, C., Wang, Y., Huang, Y. & Ma, H. Quantifying the relationship between urban development intensity and carbon dioxide emissions using a panel data analysis. *Ecological Indicators* **49**, 121–131 http://dx.doi.org/10.1016/j.ecolind.2014.10.004 (2015).
54. Li, G., Fang, C., Wang, S. & Sun, S. The effect of economic growth, urbanization, and industrialization on fine particulate matter (PM 2.5) concentrations in China. *Environ. Sci. Technol.* **50**, 11452–11459 http://dx.doi.org/10.1021/acs.est.6b02562 (2016).
55. Deng, W., Cheshmehzangi, A., Ma, Y. & Peng, Z. Promoting sustainability through governance of eco-city indicators: A multi-spatial perspective. *International Journal of Low-Carbon Technologies* **16**, 61–72 <https://doi.org/10.1093/ijlct/ctaa038> (2021).
56. Fraske, T. & Bienzeisler, B. Toward smart and sustainable traffic solutions: A case study of the geography of transitions in urban logistics. *Sustainability: Science, Practice and Policy* **16**, 353–366 <https://doi.org/10.1080/15487733.2020.1840804> (2020).
57. Fastenrath, S. & Braun, B. Sustainability transition pathways in the building sector: Energy-efficient building in Freiburg (Germany). *Applied Geography* **90**, 339–349 http://dx.doi.org/10.1016/j.apgeog.2016.09.004 (2018).
58. Najjar, R. Four dimensional spatial sustainability (4DSS): A revolutionary approach toward utopian sustainability. *Discov Sustain* **3**, 21 <https://doi.org/10.1007/s43621-022-00090-x> (2022).
59. van den Bergh, J. C. J. M. & Verbruggen, H. Spatial sustainability, trade and indicators: An evaluation of the ‘ecological footprint’. *Ecological Economics* **29**, 61–72 <https://doi.org/10.1016/S0921-8009(99)00032-4> (1999).
60. Kocsis, T. Is the Netherlands sustainable as a global-scale inner-city? Intenscoping spatial sustainability. *Ecological Economics* **101**, 103–114 http://dx.doi.org/10.1016/j.ecolecon.2014.03.002 (2014).
61. Kaklauskas, A. Degree of project utility and investment value assessments. *International Journal of Computers Communications & Control* **11**(5), 666–683 <http://dx.doi.org/10.15837/ijccc.2016.5.2679> (2016).
62. Haerpfer, C. *et al*. (eds.). World Values Survey: Round Seven - Country-Pooled Datafile Version 4.0. Madrid, Spain & Vienna, Austria: JD Systems Institute & WVSA Secretariat [https://doi.org/doi:10.14281/18241.18](https://doi.org/10.14281/18241.18) (2022).
63. Inglehart, R. *et al*. (eds.). World Values Survey: Round Six - Country-Pooled Datafile Version: [www.worldvaluessurvey.org/WVSDocumentationWV6.jsp](http://www.worldvaluessurvey.org/WVSDocumentationWV6.jsp). Madrid: JD Systems Institute (2014).
64. United Nations Environment Programme. 2021 Annual Report to the Committee of Permanent Representatives on Private Sector Engagement <https://wedocs.unep.org/20.500.11822/37738> (2021).
65. World Health Organization. Air pollution. Retrieved from <https://www.who.int/health-topics/air-pollution#tab=tab_1> (2020).
66. Kongbuamai, N., Bui, Q., Yousaf, H. M. A. U. & Liu, Y. The impact of tourism and natural resources on the ecological footprint: a case study of ASEAN countries. *Environmental Science and Pollution Research* **27**(16), 19251–19264 <http://dx.doi.org/10.1007/s11356-020-08582-x> (2020).
67. Charfeddine, L. The impact of energy consumption and economic development on ecological footprint and CO2 emissions: evidence from a Markov switching equilibrium correction model. *Energy Economics* **65**, 355–374 <http://dx.doi.org/10.1016/j.eneco.2017.05.009> (2017).
68. Zafar, M. W. *et al*. The impact of natural resources, human capital, and foreign direct investment on the ecological footprint: the case of the United States. *Resources Policy* **63**, 101428 <http://dx.doi.org/10.1016/j.resourpol.2019.101428> (2019).
69. Destek, M. A., Ulucak, R. & Dogan, E. Analyzing the environmental Kuznets curve for the EU countries: the role of ecological footprint. *Environmental Science and Pollution Research* **25**(29), 29387–29396 <http://dx.doi.org/10.1007/s11356-018-2911-4> (2018).
70. Wang, J. & Dong, K. What drives environmental degradation? Evidence from 14 Sub-Saharan African countries. *Science of the Total Environment* **656**, 165–173 <http://dx.doi.org/10.1016/j.scitotenv.2018.11.354> (2019).
71. Charfeddine, L. & Mrabet, Z. The impact of economic development and social-political factors on ecological footprint: A panel data analysis for 15 MENA countries. *Renewable and sustainable energy reviews* **76**, 138–154 <http://dx.doi.org/10.1016/j.rser.2017.03.031> (2017).
72. Cepparulo, A., Eusepi, G. & Giuriato, L. Can constitutions bring about revolutions? How to enhance decarbonization success. *Environmental Science & Policy* **93**, 200–207 <https://doi.org/10.1016/j.envsci.2018.10.019> (2019).
73. Apostoaie, C. M. & Maxim, A. Political Determinants of National Environmental Performance in the European Union. *European Journal of Sustainable Development* **6**(1), 277–290 <https://doi.org/10.14207/ejsd.2017.v6n1p277> (2017).
74. Apergis, N. The impact of greenhouse gas emissions on personal well-being: Evidence from a panel of 58 countries and aggregate and regional country samples. *Journal of Happiness Studies* **19**(1), 69–80 <http://dx.doi.org/10.1007/s10902-016-9809-y> (2018).
75. Mikayilov, J. I., Hasanov, F. J. & Galeotti, M. Decoupling of CO2 emissions and GDP: A time-varying cointegration approach. *Ecological indicators* **95**, 615–628 <http://dx.doi.org/10.1016/j.ecolind.2018.07.051> (2018).
76. Diffenbaugh, N. S. & Burke, M. Global warming has increased global economic inequality. *Proceedings of the National Academy of Sciences* **116**(20), 9808–9813 <https://doi.org/10.1073/pnas.1816020116> (2019).
77. Squalli, J. Electricity consumption and economic growth: Bounds and causality analyses of OPEC members. *Energy Economics* **29**(6), 1192–1205 <http://dx.doi.org/10.1016/j.eneco.2006.10.001> (2007).
78. Payne, J. E. On the dynamics of energy consumption and output in the US. *Applied energy* **86**(4), 575–577 <http://dx.doi.org/10.1016/j.apenergy.2008.07.003> (2009).
79. Ozturk, I. A literature survey on energy–growth nexus. *Energy policy* **38**(1), 340–349 <http://dx.doi.org/10.1016/j.enpol.2009.09.024> (2010).
80. Omri, A. An international literature survey on energy-economic growth nexus: Evidence from country-specific studies. *Renewable and Sustainable Energy Reviews* **38**, 951–959 <http://dx.doi.org/10.1016/j.rser.2014.07.084> (2014).
81. Omri, A., Mabrouk, N. B. & Sassi-Tmar. A. Modeling the causal linkages between nuclear energy, renewable energy and economic growth in developed and developing countries. *Renewable and sustainable energy reviews* **42**, 1012–1022 <http://dx.doi.org/10.1016/j.rser.2014.10.046> (2015).
82. Carleton, T. A. & Hsiang, S. M. Social and economic impacts of climate. *Science* **353**(6304), aad9837 <http://dx.doi.org/10.1126/science.aad9837> (2016).
83. Bressler, R. D., Moore, F. C., Rennert, K. & Anthoff D*.* Estimates of country level temperature-related mortality damage functions. *Sci. Rep.* **11,**20282 <https://doi.org/10.1038/s41598-021-99156-5> (2021).
84. Patz, J. A., Campbell-Lendrum, D., Holloway, T. & Foley J. A*.* Impact of regional climate change on human health. *Nature* **438,** 310–317 <https://doi.org/10.1038/nature04188> (2005).
85. Rogers, D. S. *et al*. A vision for human well-being: transition to social sustainability. *Current Opinion in Environmental Sustainability* **4**(1), 61–73 <http://dx.doi.org/10.1016/j.cosust.2012.01.013> (2012).
86. Kaklauskas, A., Abraham, A. & Milevicius, V. Diurnal emotions, valence and the coronavirus lockdown analysis in public spaces. *Engineering Applications of Artificial Intelligence* **98**, 104122 http://dx.doi.org/10.1016/j.engappai.2020.104122 (2021).
87. Kaklauskas, A. *et al.* Emotions analysis in public spaces for urban planning. *Land Use Policy* **107**, 105458 http://doi.org/10.1016/j.landusepol.2021.105458 (2021).
88. Cheshmehzangi, A. Social and Public Life During Disruptive Times: A Public Realm Perspective*.* In *Urban Health, Sustainability, and Peace in the Day the World Stopped* **14**, 123–128. Springer, Singapore <http://dx.doi.org/10.1007/978-981-16-4888-5_14> (2021).
89. Scott, A. J. Achieving a three-dimensional longevity dividend. *Nat. Aging.* **1,**500–505 <https://doi.org/10.1038/s43587-021-00074-y> (2021).
90. Jamison, D. T. *et al*. Global health 2035: a world converging within a generation. *The Lancet* **382**(9908), 1898–1955 [http://dx.doi.org/10.1016/S0140-6736(1**3)**62105-4](http://dx.doi.org/10.1016/S0140-6736(13)62105-4) (2013).
91. Acemoglu, D. & Johnson, S. Disease and development: the effect of life expectancy on economic growth. *Journal of political Economy* **115**(6), 925–985 <http://dx.doi.org/10.1086/529000> (2007).
92. Lelieveld, J., Evans, J. S., Fnais, M., Giannadaki, D. & Pozzer, A. The contribution of outdoor air pollution sources to premature mortality on a global scale. *Nature* **525,**367–371 <https://doi.org/10.1038/nature15371> (2015).
93. Juginović, A., Vuković, M., Aranza, I. & Biloš, V. Health impacts of air pollution exposure from 1990 to 2019 in 43 European countries. *Sci. Rep.* **11,**22516 <https://doi.org/10.1038/s41598-021-01802-5> (2021).
94. Al-Kindi, S. G., Brook, R. D., Biswal, S. & Rajagopalan, S*.* Environmental determinants of cardiovascular disease: lessons learned from air pollution. *Nat. Rev. Cardiol.* **17,**656–672 <https://doi.org/10.1038/s41569-020-0371-2> (2020).
95. Helliwell, J. F., Layard, R., Sachs, J. D., De Neve, J.-E., Aknin, L. B. & Wang, S. (Eds.). World Happiness Report 2022. New York: Sustainable Development Solutions Network (2022).
96. Chapman, A., Fujii, H. & Managi, S. Multinational life satisfaction, perceived inequality and energy affordability. *Nat. Sustain.* **2,**508–514 <https://doi.org/10.1038/s41893-019-0303-5> (2019).
97. Li, Y. *et al*. A psychophysical measurement on subjective well-being and air pollution. *Nat. Commun.* **10,**5473 <https://doi.org/10.1038/s41467-019-13459-w> (2019).
98. Institute for Economics & Peace. Global Peace Index 2021: Measuring Peace in a Complex World. Sydney <http://visionofhumanity.org/reports> (accessed Date Month Year) (June 2021).
99. DiRienzo, C. E. The effect of women in government on country-level peace. *Global Change, Peace & Security* **31**(1), 1–18 <http://dx.doi.org/10.1080/14781158.2018.1481023> (2019).
100. Letendre, K., Fincher, C. L. & Thornhill, R. Does infectious disease cause global variation in the frequency of intrastate armed conflict and civil war? *Biological Reviews* **85**(3), 669–683 <http://dx.doi.org/10.1111/j.1469-185X.2010.00133.x> (2010).
101. Bayar, Y. & Gavriletea, M. D. Peace, terrorism and economic growth in Middle East and North African countries. *Quality & Quantity* **52**(5), 2373–2392 <http://dx.doi.org/10.1007/s11135-017-0671-8> (2018).
102. Jaeger, W. K. *Environmental Economics For Tree Huggers And Other Skeptics* (Washington, DC: Island Press, 2005). ISBN 978-1-4416-0111-7. OCLC 232157655.
103. Purvis, B., Mao, Y. & Robinson, D. Three pillars of sustainability: in search of conceptual origins. *Sustainability science* **14**(3), 681–695 <http://dx.doi.org/10.1007/s11625-018-0627-5> (2019).
104. Hsu, A., Esty, D. C., Levy, M. A. & Sherbinin, A. D. *2016 Environmental Performance Index (EPI)*. (New Haven, CT: Yale University, 2016). Available: <https://epi.yale.edu/>.
105. Lai, S. L. & Chen, D.-N. A research on the relationship between environmental sustainability management and human development. *Sustainability* **12**, 9001 <https://doi.org/10.3390/su12219001> (2020).
106. Inglehart, R. & Welzel, C. *Modernization, cultural change, and democracy: The human development sequence* (Cambridge University Press, 2005).
107. Circle Economy. *The Circularity Gap Report 2022*. (Amsterdam: Circle Economy, 2022). Available: <https://www.circularity-gap.world/2022#Download-the-report>
108. World Bank Group. *Sustainable Investment: Best Practice Disclosure Checklist for Pension Funds*. (World Bank, 2020). Available: <https://documents1.worldbank.org/curated/en/485741587445830636/pdf/Sustainable-Investment-Best-Practice-Disclosure-Checklist-for-Pension-Funds.pdf>
109. World Bank, W. *Toolkits for Policymakers to Green the Financial System*. (World Bank, 2021). Available: <https://documents1.worldbank.org/curated/en/374051622653965991/pdf/Toolkits-for-Policymakers-to-Green-the-Financial-System.pdf>
110. United Nations. *SDG Good Practices: A compilation of success stories and lessons learned in SDG Implementation-SECOND EDITION*. (UN DESA, 2022). Available: <https://sdgs.un.org/sites/default/files/2022-03/SDGs%20Good%20Practices%20-%20second%20edition%20-%20FINAL%20FEB092022.pdf>
111. United Nations Environment Programme. *International Good Practice Principles for Sustainable Infrastructure*. (Nairobi, 2022). Available: <https://www.unep.org/resources/publication/international-good-practice-principles-sustainable-infrastructure>
112. Phillis, Y. A., Grigoroudis, E. & Kouikoglou, V. S. Sustainability ranking and improvement of countries. *Ecological Economics* **70**, 542–553 <https://doi.org/10.1016/j.ecolecon.2010.09.037> (2011).

**FIGURES**

**
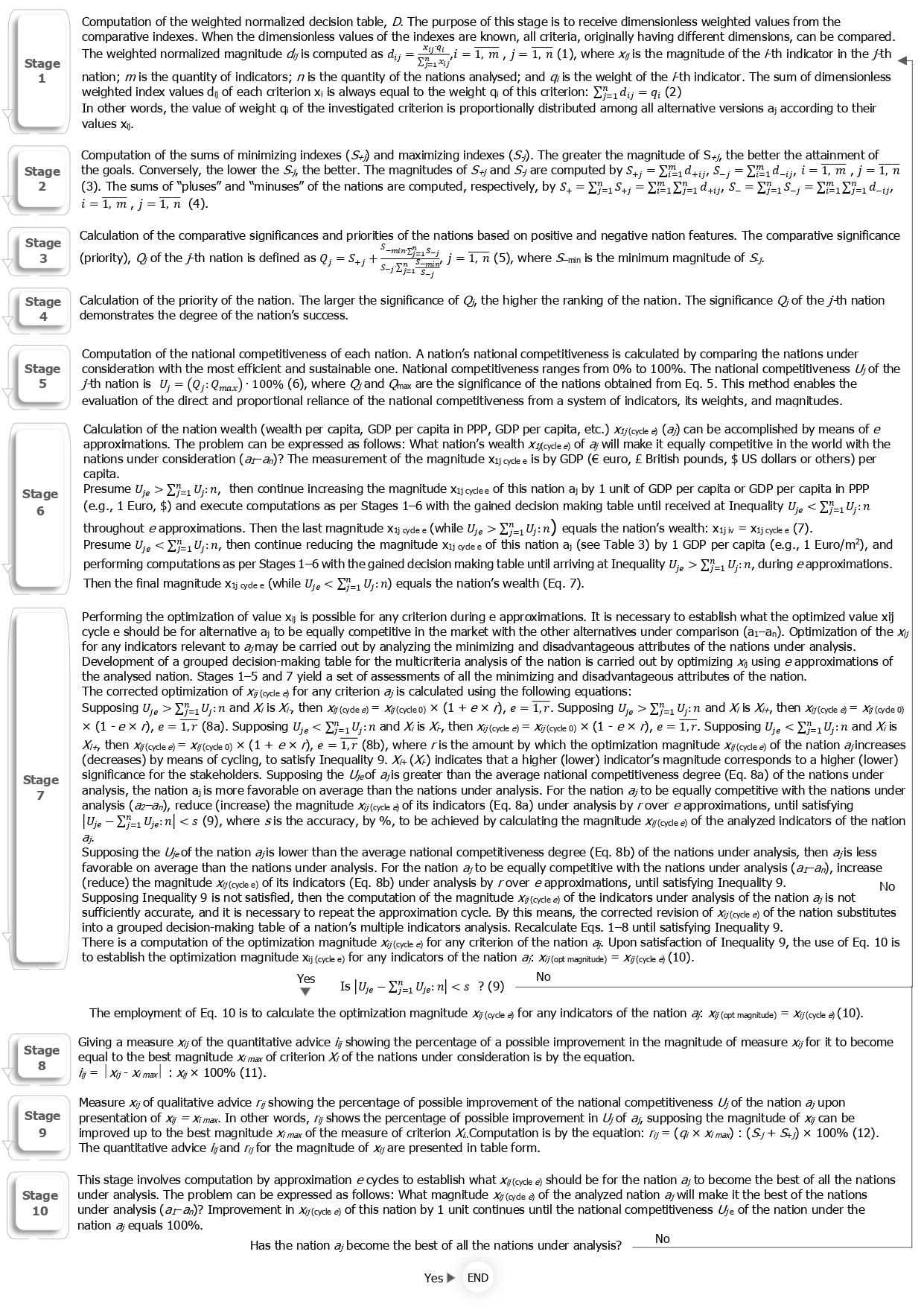
**

**Figure S1.** The INVAR method was used in the multiple criteria investigation of 173 nations.


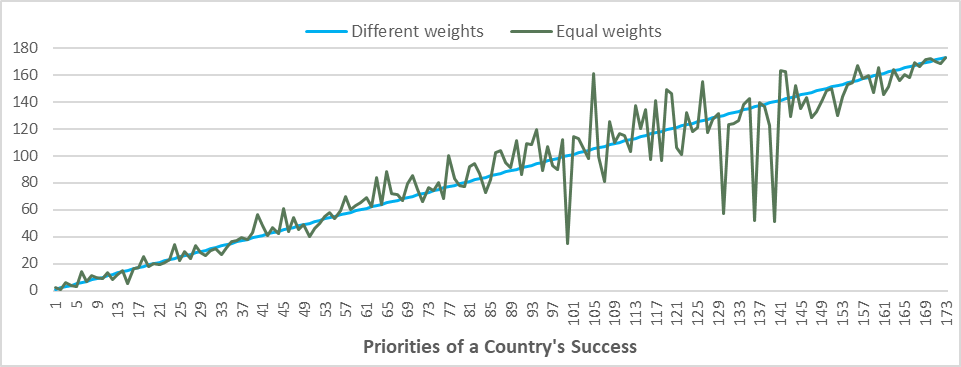


**Figure S2.** The comparison of the priorities of 173 countries calculated using equal and different weights of 17 indicators.

**TABLES**

**Table S1.** **Initial data and calculations results of 173 nations by 17 success indicators.**

| **Compared countries** | **Indicator, its weight and measuring unit**  Plus (+) or minus (-) indicates that a greater (lesser) criterion value corresponds to a greater significance by country | | | | | | | | | | | | | | | | | **Country Success** | **Country Priority** | **Country national**  **competitiveness degree, (%)** |
| --- | --- | --- | --- | --- | --- | --- | --- | --- | --- | --- | --- | --- | --- | --- | --- | --- | --- | --- | --- | --- |
|  | **GDP per capita,**  **1, USD, +** | **GDP per capita in PPP,**  **1, USD, +** | **Ease of doing business ranking,**  **1, Rank, -** | **Corruption perceptions index,**  **1, Index, +** | **Human development index,**  **1, Index, +** | **Global gender gap,**  **1, Index, +** | **Economic freedom, 1, Index, +** | **Democracy index,**  **1, Index, +** | **Unemployment rate,**  **1, %, -** | **Fragile state index,**  **1, Index, -** | **Economic decline Index,**  **1, Index, -** | **Government effectiveness, 1, Points, +** | **Civil liberties, 1, Points, -** | **The global sustainable competitiveness index, 1, Points, +** | **Population growth, 1, %, +** | **Human rights and rule of law index, 1, Points, -** | **Wealth per adult, 1, USD, +** |  |  |  |
|  | **V03** | **V04** | **V05** | **V06** | **V07** | **V08** | **V09** | **V10** | **V11** | **V12** | **V13** | **V14** | **V15** | **V16** | **V17** | **V18** | **V19** | **V01** | **V02** |  |
| Algeria | 3306.86 | 11324.24 | 157 | 36 | 0.748 | 0.634 | 46.9 | 3.77 | 12.55 | 74.6 | 6.2 | 0.38 | 5 | 41.1 | 0.64 | 6.3 | 8.87 | 0.067 | 134 | 27.12% |
| Afghanistan | 516.75 | 2078.65 | 173 | 19 | 0.511 | 0.564 | 54.7 | 2.85 | 11.71 | 102.9 | 8.3 | 0.17 | 6 | 35 | 0.72 | 7.6 | 1.74 | 0.0524 | 171 | 21.23% |
| Albania | 5246.29 | 13439.67 | 82 | 36 | 0.795 | 0.769 | 66.9 | 6.08 | 13.33 | 58.8 | 5.8 | 0.47 | 3 | 47.4 | 0.2 | 3.9 | 30.52 | 0.0796 | 92 | 32.21% |
| Angola | 1776.17 | 6445.43 | 177 | 27 | 0.581 | 0.66 | 52.2 | 3.66 | 8.33 | 87.3 | 6.9 | 0.24 | 5 | 40.3 | 0.89 | 6.4 | 3.53 | 0.0634 | 148 | 25.66% |
| Antigua and Barbuda | 13992.74 | 18241.87 | 113 | 55 | 0.778 | 0.7 | 53 | 6.32 | 8.1 | 52.1 | 4.8 | 0.46 | 2 | 44.5 | 0.46 | 4.2 | 22.7 | 0.0914 | 63 | 36.99% |
| Argentina | 8579.02 | 20770.73 | 126 | 42 | 0.845 | 0.746 | 53.1 | 6.95 | 11.46 | 46.1 | 5.4 | 0.45 | 2 | 48.1 | 0.48 | 3.6 | 7.22 | 0.0869 | 69 | 35.19% |
| Armenia | 4266.02 | 13312.11 | 47 | 49 | 0.776 | 0.684 | 70.6 | 5.35 | 21.21 | 64.2 | 6.2 | 0.47 | 4 | 47.2 | 0.34 | 6.3 | 22.57 | 0.0752 | 111 | 30.44% |
| Australia | 51692.84 | 53329.81 | 14 | 77 | 0.944 | 0.731 | 82.6 | 8.96 | 6.46 | 19.7 | 1.3 | 0.84 | 1 | 50.2 | 0.53 | 1.4 | 483.76 | 0.2129 | 7 | 86.20% |
| Austria | 48586.8 | 55683.84 | 27 | 76 | 0.922 | 0.744 | 73.3 | 8.16 | 5.36 | 24.1 | 1.3 | 0.85 | 1 | 56.7 | 0.38 | 0.6 | 290.35 | 0.2012 | 12 | 81.47% |
| Azerbaijan | 4221.41 | 14479.71 | 28 | 30 | 0.756 | 0.687 | 69.3 | 2.68 | 6.46 | 71.3 | 4.4 | 0.46 | 6 | 45.1 | 0.43 | 8 | 11.93 | 0.0743 | 114 | 30.09% |
| Bahrain | 20409.95 | 43755.86 | 43 | 42 | 0.852 | 0.629 | 66.3 | 2.49 | 1.78 | 63.9 | 3.7 | 0.59 | 6 | 41.3 | 0.96 | 8.7 | 87.56 | 0.1048 | 48 | 42.43% |
| Bangladesh | 1961.61 | 5138.65 | 168 | 26 | 0.632 | 0.726 | 56.4 | 5.99 | 5.41 | 85.7 | 5.8 | 0.33 | 5 | 43.3 | 0.49 | 7 | 7.84 | 0.0658 | 139 | 26.63% |
| Barbados | 15373.85 | 13349.57 | 128 | 64 | 0.814 | 0.749 | 61.4 | 6.32 | 10.38 | 46.4 | 5.4 | 0.6 | 1 | 44.8 | 0.33 | 3.4 | 63.26 | 0.0961 | 60 | 38.89% |
| [Belarus](http://iti3.vgtu.lt/SAVAS/Showobjektas.aspx?objid=142234) | 6424.15 | 20239.2 | 49 | 47 | 0.823 | 0.746 | 61.7 | 2.59 | 4.77 | 65.8 | 4.9 | 0.34 | 6 | 48.6 | 0.23 | 7.4 | 23.28 | 0.0773 | 100 | 31.30% |
| [Belgium](http://iti3.vgtu.lt/SAVAS/Showobjektas.aspx?objid=142169) | 45159.35 | 53034.96 | 46 | 76 | 0.931 | 0.75 | 68.9 | 7.51 | 5.55 | 27.1 | 3.9 | 0.74 | 1 | 52.1 | 0.41 | 0.7 | 351.33 | 0.1753 | 17 | 70.97% |
| Belize | 4115.18 | 6457.64 | 135 | 24 | 0.716 | 0.671 | 57.4 | 4.97 | 8.46 | 60.8 | 6 | 0.36 | 2 | 48.2 | 0.64 | 5.4 | 10.36 | 0.0743 | 115 | 30.08% |
| Benin | 1291.04 | 3505.42 | 149 | 41 | 0.545 | 0.658 | 55.2 | 4.58 | 1.58 | 72.5 | 6.5 | 0.44 | 2 | 40.8 | 0.79 | 4.6 | 2.56 | 0.0766 | 106 | 31% |
| Bhutan | 3000.78 | 11130.22 | 89 | 68 | 0.654 | 0.635 | 62.1 | 5.71 | 3.65 | 69.5 | 5 | 0.58 | 4 | 49 | 0.51 | 5.7 | 18.88 | 0.0856 | 73 | 34.65% |
| Bolivia | 3133.1 | 8275.49 | 150 | 31 | 0.718 | 0.734 | 42.8 | 5.08 | 7.9 | 75 | 5.1 | 0.38 | 3 | 50 | 0.56 | 6.2 | 12.29 | 0.0719 | 120 | 29.13% |
| Bosnia and Herzegovina | 6079.74 | 15623.1 | 90 | 35 | 0.78 | 0.712 | 62.6 | 4.84 | 15.27 | 70.2 | 5.9 | 0.29 | 4 | 50.5 | 0.19 | 5.1 | 30.6 | 0.0722 | 119 | 29.21% |
| Botswana | 6404.9 | 15458.48 | 87 | 60 | 0.735 | 0.709 | 69.6 | 7.62 | 24.93 | 57.1 | 5.5 | 0.55 | 2 | 42.2 | 0.68 | 4.9 | 15.6 | 0.0846 | 75 | 34.26% |
| Brazil | 6796.84 | 14835.41 | 124 | 38 | 0.765 | 0.691 | 53.7 | 6.92 | 13.69 | 73 | 5.2 | 0.4 | 2 | 49.1 | 0.44 | 7 | 18.27 | 0.0772 | 102 | 31.24% |
| Bulgaria | 10079.2 | 24613.84 | 61 | 44 | 0.816 | 0.727 | 70.2 | 6.71 | 5.12 | 49.2 | 4.9 | 0.48 | 2 | 51.6 | 0.2 | 3 | 36.44 | 0.1012 | 53 | 40.98% |
| Burkina Faso | 857.93 | 2274.02 | 151 | 40 | 0.452 | 0.635 | 56.7 | 3.73 | 4.89 | 85.9 | 6.7 | 0.35 | 4 | 42.4 | 0.82 | 5.7 | 1.68 | 0.0673 | 133 | 27.25% |
| Burundi | 238.99 | 771.16 | 166 | 19 | 0.433 | 0.745 | 49 | 2.14 | 1.71 | 97.9 | 8.5 | 0.23 | 6 | 38.4 | 0.86 | 9.1 | 728 | 0.1014 | 52 | 41.06% |
| Cabo Verde | 3064.27 | 6376.64 | 137 | 58 | 0.665 | 0.725 | 63.6 | 7.65 | 15.31 | 64.8 | 5.7 | 0.55 | 1 | 43 | 0.5 | 3.1 | 6.17 | 0.0808 | 89 | 32.71% |
| Cambodia | 1543.67 | 4421.48 | 144 | 21 | 0.594 | 0.694 | 57.3 | 3.1 | 0.33 | 80.3 | 5 | 0.41 | 5 | 45.9 | 0.56 | 7.3 | 5.9 | 0.0667 | 136 | 27% |
| Cameroon | 1537.13 | 3867.52 | 167 | 25 | 0.563 | 0.686 | 53.6 | 2.77 | 3.84 | 97.9 | 6.8 | 0.31 | 6 | 46 | 0.77 | 7.8 | 3.04 | 0.0618 | 154 | 25.04% |
| Canada | 43294.65 | 46611.31 | 23 | 77 | 0.929 | 0.772 | 78.2 | 9.24 | 9.46 | 18.7 | 1.2 | 0.85 | 1 | 51.3 | 0.5 | 1.4 | 332.32 | 0.1841 | 16 | 74.52% |
| Central African Republic | 492.8 | 987.64 | 184 | 26 | 0.397 | 0.686 | 50.7 | 1.32 | 6.36 | 107.5 | 8.4 | 0.13 | 7 | 36.9 | 0.63 | 9.2 | 840 | 0.1017 | 51 | 41.18% |
| Chad | 659.27 | 1602.36 | 182 | 21 | 0.398 | 0.596 | 50.2 | 1.55 | 1.74 | 106.4 | 8.5 | 0.18 | 6 | 37.1 | 0.84 | 8.5 | 1.12 | 0.0535 | 168 | 21.67% |
| Chile | 13231.7 | 25110.16 | 59 | 67 | 0.851 | 0.723 | 76.8 | 8.28 | 11.18 | 42.5 | 3.6 | 0.71 | 2 | 50.6 | 0.46 | 3.9 | 53.59 | 0.1101 | 42 | 44.56% |
| China | 10434.78 | 17210.76 | 32 | 42 | 0.761 | 0.676 | 59.5 | 2.27 | 5 | 69.9 | 3.9 | 0.64 | 6 | 50.8 | 0.35 | 9.3 | 67.77 | 0.0835 | 79 | 33.81% |
| Cyprus | 27527.85 | 39452.87 | 54 | 57 | 0.887 | 0.692 | 70.1 | 7.56 | 7.59 | 56.1 | 5.1 | 0.69 | 1 | 47.6 | 0.44 | 2.9 | 142.3 | 0.1256 | 36 | 50.86% |
| Colombia | 5334.56 | 14931.05 | 67 | 39 | 0.767 | 0.758 | 69.2 | 7.04 | 15.04 | 76.6 | 4.8 | 0.51 | 3 | 47.4 | 0.5 | 6.5 | 16.93 | 0.0818 | 83 | 33.11% |
| Comoros | 1420.66 | 3153.13 | 160 | 21 | 0.554 | 0.719 | 53.7 | 3.09 | 9.22 | 81.2 | 7.3 | 0.15 | 4 | 38.6 | 0.7 | 5.8 | 5.4 | 0.0597 | 160 | 24.18% |
| Congo Dem Rep | 543.95 | 1141.82 | 183 | 18 | 0.48 | 0.578 | 49.5 | 1.13 | 5.27 | 109.4 | 8 | 0.13 | 6 | 44.5 | 0.88 | 9.5 | 1.24 | 0.0525 | 170 | 21.25% |
| Congo Rep | 1846.13 | 3622.32 | 180 | 19 | 0.574 | 0.596 | 41.8 | 3.11 | 22.84 | 92.1 | 7.8 | 0.19 | 5 | 41.7 | 0.76 | 8.1 | 2.18 | 0.053 | 169 | 21.45% |
| Costa Rica | 12140.85 | 22132.47 | 74 | 57 | 0.81 | 0.782 | 65.8 | 8.16 | 17.41 | 40.2 | 4.6 | 0.57 | 1 | 52.6 | 0.47 | 1.2 | 44.34 | 0.1023 | 50 | 41.42% |
| Cote d'Ivoire | 2325.72 | 5465.51 | 110 | 36 | 0.538 | 0.606 | 59.7 | 4.11 | 3.49 | 89.7 | 6.4 | 0.39 | 4 | 45.3 | 0.77 | 7 | 6.62 | 0.071 | 124 | 28.76% |
| Croatia | 14134.16 | 28841.71 | 51 | 47 | 0.851 | 0.72 | 62.2 | 6.5 | 7.51 | 46.1 | 5 | 0.59 | 2 | 57.2 | 0.22 | 2.9 | 69.14 | 0.1068 | 45 | 43.25% |
| Czech Republic | 22931.27 | 41603.99 | 41 | 54 | 0.9 | 0.706 | 74.8 | 7.67 | 2.55 | 35.7 | 3.7 | 0.7 | 1 | 55.2 | 0.35 | 2.1 | 78.1 | 0.1434 | 26 | 58.05% |
| Denmark | 61063.32 | 60229.91 | 4 | 88 | 0.94 | 0.782 | 78.3 | 9.15 | 5.64 | 17.2 | 1.3 | 0.9 | 1 | 61 | 0.36 | 1.4 | 376.07 | 0.2262 | 4 | 91.57% |
| Djibouti | 3425.48 | 5781.76 | 112 | 27 | 0.524 | 0.705 | 52.9 | 2.71 | 28.39 | 82.7 | 6.7 | 0.35 | 5 | 41.4 | 0.57 | 7.4 | 3.11 | 0.0572 | 165 | 23.14% |
| Dominican Republic | 7268.2 | 17935.66 | 115 | 28 | 0.756 | 0.7 | 60.9 | 6.32 | 6.13 | 64.4 | 4.6 | 0.43 | 3 | 44.5 | 0.49 | 5 | 22.7 | 0.0835 | 80 | 33.80% |
| Ecuador | 5600.39 | 10895.77 | 129 | 39 | 0.759 | 0.729 | 51.3 | 6.13 | 6.11 | 69.4 | 5.4 | 0.4 | 3 | 46.6 | 0.59 | 3.9 | 17.15 | 0.0812 | 87 | 32.87% |
| Egypt | 3569.21 | 12607 | 114 | 33 | 0.707 | 0.629 | 54 | 2.93 | 9.17 | 86 | 7.6 | 0.38 | 6 | 38.9 | 0.65 | 9.7 | 19.47 | 0.0656 | 141 | 26.56% |
| [El Salvador](http://iti3.vgtu.lt/SAVAS/Showobjektas.aspx?objid=142280) | 3798.64 | 8420.49 | 91 | 36 | 0.673 | 0.706 | 61.6 | 5.9 | 6.25 | 68.9 | 5.1 | 0.42 | 4 | 44.7 | 0.4 | 5.4 | 34 | 0.0776 | 99 | 31.41% |
| Equatorial Guinea | 7143.24 | 17940.52 | 178 | 16 | 0.592 | 0.686 | 48.3 | 1.92 | 9.9 | 83 | 5.7 | 0.18 | 7 | 40.8 | 0.93 | 8.3 | 18.25 | 0.0644 | 146 | 26.06% |
| Estonia | 23027.03 | 37600.59 | 18 | 75 | 0.892 | 0.751 | 77.7 | 7.84 | 6.8 | 38.5 | 2.8 | 0.79 | 1 | 59.4 | 0.36 | 1.4 | 77.82 | 0.1478 | 24 | 59.84% |
| Eswatini | 3424.28 | 8865.74 | 121 | 33 | 0.611 | 0.703 | 55.3 | 3.08 | 25.51 | 83 | 9.5 | 0.33 | 5 | 43.1 | 0.49 | 8.5 | 13.83 | 0.059 | 163 | 23.90% |
| Ethiopia | 936.34 | 2422.81 | 159 | 38 | 0.485 | 0.705 | 53.6 | 3.38 | 3.24 | 94.6 | 6.1 | 0.38 | 6 | 47 | 0.77 | 7.9 | 3.54 | 0.0649 | 144 | 26.27% |
| Fiji | 5057.63 | 12078.84 | 102 | 43 | 0.743 | 0.678 | 63.4 | 5.72 | 4.72 | 69.9 | 5.6 | 0.56 | 3 | 46.7 | 0.44 | 6 | 15.71 | 0.0825 | 82 | 33.38% |
| Finland | 48744.99 | 50506.29 | 20 | 85 | 0.938 | 0.832 | 75.7 | 9.2 | 7.76 | 14.6 | 2.6 | 0.92 | 1 | 60.4 | 0.34 | 0.5 | 167.71 | 0.1844 | 15 | 74.63% |
| France | 39030.36 | 46983.04 | 33 | 69 | 0.901 | 0.781 | 66 | 7.99 | 8.01 | 30.5 | 3.4 | 0.77 | 2 | 55.5 | 0.34 | 1.3 | 299.36 | 0.1576 | 21 | 63.82% |
| Gabon | 6881.72 | 15106.37 | 169 | 30 | 0.703 | 0.596 | 56.7 | 3.54 | 21.97 | 69.1 | 5.7 | 0.3 | 5 | 43.9 | 0.74 | 7.2 | 13.7 | 0.0666 | 137 | 26.95% |
| Gambia | 773 | 2275.06 | 155 | 37 | 0.496 | 0.628 | 56.3 | 4.49 | 11.08 | 82.2 | 8.1 | 0.35 | 4 | 41.5 | 0.83 | 8.1 | 2.5 | 0.0636 | 147 | 25.76% |
| Georgia | 4266.69 | 14766.98 | 7 | 56 | 0.812 | 0.708 | 77.1 | 5.31 | 18.5 | 71.2 | 5.3 | 0.67 | 3 | 51.2 | 0.28 | 5.1 | 14.16 | 0.0843 | 76 | 34.12% |
| Germany | 46208.43 | 54792.06 | 22 | 80 | 0.947 | 0.787 | 73.5 | 8.67 | 3.81 | 23.2 | 1.3 | 0.79 | 1 | 54.6 | 0.34 | 0.8 | 268.68 | 0.2093 | 8 | 84.73% |
| Ghana | 2205.53 | 5744.43 | 118 | 43 | 0.611 | 0.673 | 59.4 | 6.5 | 4.65 | 64.2 | 5.4 | 0.46 | 2 | 48.5 | 0.69 | 4.7 | 6.13 | 0.0829 | 81 | 33.55% |
| Greece | 17622.54 | 27909.54 | 79 | 50 | 0.888 | 0.701 | 59.9 | 7.39 | 16.3 | 52.1 | 5.2 | 0.59 | 2 | 50 | 0.3 | 3 | 104.6 | 0.1006 | 54 | 40.74% |
| Grenada | 9261.55 | 15283.25 | 146 | 53 | 0.779 | 0.749 | 65 | 7.16 | 12.79 | 55.2 | 5.3 | 0.48 | 2 | 42.6 | 0.39 | 2.4 | 21.04 | 0.0861 | 71 | 34.87% |
| Guatemala | 4603.34 | 8853.67 | 96 | 25 | 0.663 | 0.666 | 64 | 4.97 | 3.55 | 79.2 | 4.8 | 0.35 | 4 | 41.6 | 0.58 | 7 | 30.59 | 0.0753 | 110 | 30.50% |
| Guyana | 6955.94 | 19704.58 | 134 | 41 | 0.682 | 0.707 | 56.2 | 6.01 | 16.43 | 66 | 5.5 | 0.4 | 3 | 48.7 | 0.39 | 3.2 | 12.28 | 0.077 | 104 | 31.18% |
| Guinea | 1194.04 | 2817.32 | 156 | 28 | 0.477 | 0.642 | 56.5 | 3.08 | 6.1 | 97.2 | 8.3 | 0.3 | 5 | 42 | 0.81 | 6.8 | 2.94 | 0.0612 | 156 | 24.78% |
| Guinea-Bissau | 727.52 | 1948.8 | 174 | 19 | 0.48 | 0.642 | 53.3 | 2.63 | 6.74 | 92.9 | 7.4 | 0.19 | 4 | 39.4 | 0.75 | 6.9 | 1.83 | 0.057 | 166 | 23.08% |
| Haiti | 1272.37 | 3095.19 | 179 | 18 | 0.51 | 0.746 | 52.3 | 4.22 | 15.45 | 97.7 | 8.5 | 0.06 | 5 | 35.5 | 0.53 | 6.9 | 767 | 0.0997 | 57 | 40.38% |
| Honduras | 2389.01 | 5420.23 | 133 | 24 | 0.634 | 0.722 | 61.1 | 5.36 | 8.37 | 76.8 | 5.8 | 0.37 | 4 | 42.2 | 0.6 | 6.9 | 15.38 | 0.0691 | 127 | 27.97% |
| Hong Kong SAR China | 46323.86 | 59234.11 | 3 | 77 | 0.949 | 0.676 | 89.1 | 5.57 | 5.83 | 63.3 | 7.3 | 0.85 | 3 | 50.8 | 0.24 | 9.3 | 503.34 | 0.153 | 23 | 61.94% |
| Hungary | 15980.74 | 33077.23 | 52 | 44 | 0.854 | 0.677 | 66.4 | 6.56 | 4.25 | 47.6 | 4.5 | 0.62 | 3 | 52.9 | 0.27 | 5 | 53.66 | 0.1056 | 47 | 42.76% |
| Iceland | 59270.18 | 53622.25 | 26 | 75 | 0.949 | 0.877 | 77.1 | 9.37 | 5.48 | 17.8 | 2.8 | 0.82 | 1 | 60.7 | 0.6 | 0.7 | 337.79 | 0.2052 | 11 | 83.07% |
| Yemen | 824.1 | 3688.5 | 187 | 15 | 0.47 | 0.494 | 65 | 1.95 | 13.39 | 112.4 | 9.4 | 0.001 | 6 | 34.9 | 0.71 | 10 | 5.58 | 0.0475 | 173 | 19.23% |
| India | 1927.71 | 6503.95 | 62 | 40 | 0.645 | 0.668 | 56.5 | 6.61 | 8 | 75.3 | 5.6 | 0.58 | 3 | 42.4 | 0.49 | 7.6 | 14.25 | 0.0772 | 103 | 31.23% |
| Indonesia | 3869.59 | 12072.74 | 73 | 37 | 0.718 | 0.7 | 67.2 | 6.3 | 4.28 | 67.8 | 4.2 | 0.58 | 4 | 47.4 | 0.5 | 6.7 | 17.69 | 0.0843 | 77 | 34.11% |
| Iran, Islamic Rep. | 2422.48 | 13338.01 | 127 | 25 | 0.783 | 0.584 | 49.2 | 2.2 | 12.17 | 83.4 | 7.2 | 0.28 | 6 | 45.3 | 0.54 | 9.2 | 22.25 | 0.0613 | 155 | 24.80% |
| Iraq | 4145.86 | 9506.73 | 172 | 21 | 0.674 | 0.53 | 47 | 3.62 | 14.09 | 95.9 | 5.6 | 0.21 | 6 | 33.9 | 0.72 | 7.8 | 14.51 | 0.0591 | 162 | 23.93% |
| Ireland | 85267.76 | 93180.95 | 24 | 72 | 0.955 | 0.798 | 80.9 | 9.05 | 5.62 | 19.9 | 2.5 | 0.82 | 1 | 56.8 | 0.53 | 1.8 | 266.15 | 0.2141 | 6 | 86.68% |
| Israel | 44168.94 | 39481.57 | 35 | 60 | 0.919 | 0.718 | 74 | 7.84 | 4.33 | 75.1 | 3.9 | 0.73 | 3 | 48.9 | 0.63 | 6.6 | 228.27 | 0.1362 | 29 | 55.14% |
| Italy | 31714.22 | 41828.55 | 58 | 53 | 0.892 | 0.707 | 63.8 | 7.74 | 9.16 | 42.4 | 4.9 | 0.58 | 1 | 51.6 | 0.25 | 1.1 | 239.24 | 0.1343 | 30 | 54.36% |
| Jamaica | 4664.53 | 9241.58 | 71 | 44 | 0.734 | 0.735 | 68.5 | 7.13 | 9.48 | 60 | 6.2 | 0.59 | 2 | 42.5 | 0.38 | 5.5 | 19.89 | 0.0842 | 78 | 34.08% |
| Japan | 40193.25 | 42390.38 | 30 | 74 | 0.919 | 0.652 | 73.3 | 8.13 | 2.8 | 32.3 | 3.3 | 0.84 | 1 | 52.5 | 0.24 | 3.2 | 256.6 | 0.1652 | 19 | 66.89% |
| Jordan | 4282.77 | 10355.01 | 75 | 49 | 0.729 | 0.623 | 66 | 3.62 | 19.03 | 75.4 | 6.5 | 0.52 | 5 | 39.5 | 0.49 | 7.1 | 28.32 | 0.071 | 125 | 28.73% |
| Kazakhstan | 9122.23 | 26754.38 | 25 | 38 | 0.825 | 0.71 | 69.6 | 3.14 | 4.89 | 59.8 | 5.3 | 0.53 | 5 | 47.1 | 0.54 | 6.4 | 33.46 | 0.0902 | 65 | 36.50% |
| Kenya | 1878.58 | 4577.91 | 56 | 31 | 0.601 | 0.671 | 55.3 | 5.05 | 5.73 | 90.3 | 6.5 | 0.42 | 4 | 46.5 | 0.71 | 6.5 | 12.31 | 0.0735 | 117 | 29.76% |
| Kyrgyz Republic | 1173.61 | 4964.73 | 80 | 31 | 0.697 | 0.689 | 62.9 | 4.21 | 8.71 | 73.9 | 6.1 | 0.38 | 4 | 46.6 | 0.68 | 6.6 | 5.82 | 0.0716 | 121 | 28.99% |
| Korea, Rep. | 31631.47 | 45274.46 | 5 | 61 | 0.916 | 0.672 | 74 | 8.01 | 3.93 | 32 | 1.7 | 0.8 | 2 | 51.3 | 0.33 | 3.1 | 211.37 | 0.1631 | 20 | 66.03% |
| Kosovo | 4346.64 | 11387.65 | 57 | 36 | 0.829 | 0.71 | 67.4 | 6.22 | 15.86 | 58.5 | 6 | 0.43 | 3 | 50.7 | 0.17 | 3.8 | 46.09 | 0.0791 | 95 | 32.02% |
| Kuwait | 24811.77 | 47306.99 | 83 | 42 | 0.806 | 0.65 | 63.2 | 3.8 | 3.54 | 50.9 | 2.4 | 0.46 | 5 | 41.2 | 0.58 | 7 | 129.89 | 0.108 | 43 | 43.71% |
| Lao PDR | 2629.71 | 8239.17 | 154 | 29 | 0.613 | 0.731 | 55.5 | 1.77 | 1.03 | 76.9 | 5.1 | 0.33 | 6 | 46.5 | 0.57 | 7 | 7.38 | 0.0662 | 138 | 26.80% |
| Latvia | 17726.25 | 31464.48 | 19 | 57 | 0.866 | 0.785 | 71.9 | 7.24 | 8.1 | 42.3 | 3.2 | 0.69 | 2 | 58.2 | 0.19 | 2.7 | 70.45 | 0.1219 | 37 | 49.33% |
| Lebanon | 4649.55 | 12118.57 | 143 | 25 | 0.744 | 0.599 | 51.7 | 4.16 | 13.3 | 84.7 | 7 | 0.25 | 4 | 37.9 | 0.23 | 6.9 | 55.01 | 0.0629 | 149 | 25.45% |
| Lesotho | 875.35 | 2444.08 | 122 | 41 | 0.527 | 0.695 | 54.5 | 6.3 | 24.56 | 78.3 | 8.4 | 0.3 | 3 | 41.3 | 0.45 | 5.1 | 1.23 | 0.0611 | 157 | 24.75% |
| Liberia | 632.94 | 1468.36 | 175 | 28 | 0.48 | 0.685 | 49 | 5.32 | 3.98 | 90 | 8.3 | 0.19 | 4 | 41.4 | 0.74 | 6.2 | 4.45 | 0.0622 | 151 | 25.19% |
| Libya | 3699.29 | 10846.29 | 186 | 17 | 0.724 | 0.629 | 56 | 1.95 | 20.07 | 95.2 | 8 | 0.06 | 6 | 41.8 | 0.56 | 9.4 | 17.2 | 0.0537 | 167 | 21.76% |
| Lithuania | 20233.64 | 38883.12 | 11 | 60 | 0.882 | 0.745 | 76.7 | 7.13 | 8.49 | 36.5 | 3.6 | 0.72 | 1 | 55.9 | 0.31 | 2.4 | 63.5 | 0.1322 | 32 | 53.52% |
| Luxembourg | 116014.6 | 117500.21 | 72 | 80 | 0.916 | 0.725 | 75.8 | 8.68 | 6.77 | 18.8 | 1.5 | 0.89 | 1 | 58 | 0.66 | 1.2 | 477.31 | 0.2387 | 2 | 96.64% |
| Macao SAR China | 39403.14 | 60895.52 | 32 | 77 | 0.761 | 0.719 | 70.3 | 5.57 | 2.57 | 64.7 | 7.3 | 0.74 | 6 | 50.8 | 0.56 | 9.3 | 271.68 | 0.1318 | 33 | 53.37% |
| Madagascar | 471.49 | 1544.16 | 161 | 25 | 0.528 | 0.719 | 60.5 | 5.7 | 2.47 | 79.5 | 7 | 0.28 | 3 | 39.2 | 0.79 | 5.8 | 1.96 | 0.0689 | 128 | 27.90% |
| Malaysia | 10412.35 | 27923.69 | 12 | 51 | 0.81 | 0.677 | 74.7 | 7.19 | 4.5 | 57.6 | 2.8 | 0.72 | 4 | 47 | 0.54 | 7.1 | 29.29 | 0.1046 | 49 | 42.36% |
| Malawi | 636.82 | 1591.61 | 109 | 30 | 0.483 | 0.664 | 52.8 | 5.74 | 6.7 | 84 | 7.7 | 0.33 | 3 | 39.9 | 0.79 | 5.6 | 2.05 | 0.0684 | 130 | 27.68% |
| Maldives | 6924.11 | 13443.48 | 147 | 43 | 0.74 | 0.646 | 56.5 | 6.14 | 6.33 | 66.2 | 5 | 0.47 | 5 | 48 | 0.63 | 7.4 | 25.51 | 0.0793 | 94 | 32.09% |
| Mali | 862.45 | 2347.99 | 148 | 30 | 0.434 | 0.621 | 55.9 | 3.93 | 7.7 | 96 | 7.3 | 0.25 | 5 | 39.6 | 0.85 | 7.7 | 2.42 | 0.0609 | 158 | 24.65% |
| Malta | 27884.64 | 41740.77 | 88 | 53 | 0.895 | 0.693 | 69.5 | 7.68 | 4.26 | 33.6 | 3.4 | 0.72 | 1 | 50.9 | 1.06 | 3.1 | 148.93 | 0.1422 | 28 | 57.57% |
| Mauritania | 1701.99 | 5390.09 | 152 | 29 | 0.546 | 0.614 | 55.3 | 3.92 | 11.27 | 88.7 | 6.8 | 0.33 | 5 | 35.1 | 0.8 | 7.2 | 2.79 | 0.0623 | 150 | 25.21% |
| Mauritius | 8627.84 | 20530.51 | 13 | 53 | 0.804 | 0.665 | 74.9 | 8.14 | 7.41 | 37.2 | 4.2 | 0.68 | 2 | 49.5 | 0.31 | 3.8 | 63.37 | 0.1119 | 40 | 45.29% |
| Mexico | 8329.27 | 18444.07 | 60 | 31 | 0.779 | 0.754 | 66 | 6.07 | 4.45 | 67.2 | 4.2 | 0.46 | 3 | 46.4 | 0.5 | 6 | 42.69 | 0.0912 | 64 | 36.91% |
| Myanmar | 1467.6 | 5123.83 | 165 | 28 | 0.583 | 0.665 | 54 | 3.04 | 1.06 | 94 | 5 | 0.28 | 6 | 45.9 | 0.43 | 9.4 | 5.03 | 0.06 | 159 | 24.30% |
| Moldova | 4547.06 | 13000.4 | 48 | 34 | 0.75 | 0.757 | 62 | 5.78 | 3.82 | 66 | 5.5 | 0.4 | 4 | 49.8 | 0 | 4.8 | 15.49 | 0.0798 | 91 | 32.31% |
| Mongolia | 4060.95 | 12366.95 | 81 | 35 | 0.737 | 0.706 | 55.9 | 6.48 | 7.01 | 51.9 | 4.5 | 0.42 | 2 | 43.8 | 0.6 | 4 | 6.32 | 0.0875 | 68 | 35.41% |
| Montenegro | 7677.15 | 20029.91 | 50 | 45 | 0.829 | 0.71 | 61.5 | 5.77 | 17.9 | 55.5 | 5 | 0.49 | 3 | 48.1 | 0.28 | 3.7 | 60.31 | 0.0857 | 72 | 34.70% |
| Morocco | 3058.69 | 7369.49 | 53 | 40 | 0.686 | 0.605 | 63.3 | 5.04 | 11.45 | 71.2 | 5.2 | 0.49 | 5 | 41.8 | 0.52 | 5.9 | 13.46 | 0.074 | 116 | 29.97% |
| Mozambique | 448.54 | 1297.4 | 138 | 25 | 0.456 | 0.723 | 50.5 | 3.51 | 3.81 | 91.7 | 8.3 | 0.34 | 4 | 43.7 | 0.83 | 5.3 | 1 | 0.0647 | 145 | 26.21% |
| Namibia | 4179.28 | 9298.09 | 104 | 51 | 0.646 | 0.784 | 60.9 | 6.52 | 21.45 | 65.1 | 6.9 | 0.51 | 2 | 40.6 | 0.64 | 2.9 | 15.29 | 0.0781 | 98 | 31.63% |
| Nepal | 1155.14 | 4008.51 | 94 | 33 | 0.602 | 0.68 | 54.2 | 5.22 | 4.72 | 82.6 | 5.3 | 0.29 | 4 | 49.4 | 0.64 | 6.8 | 4.06 | 0.0709 | 126 | 28.70% |
| Netherlands | 52397.12 | 59268.14 | 42 | 82 | 0.944 | 0.736 | 77 | 8.96 | 3.82 | 22.9 | 1.8 | 0.9 | 1 | 52.9 | 0.41 | 0.9 | 377.09 | 0.2067 | 10 | 83.68% |
| New Zealand | 41441.47 | 44491.32 | 1 | 88 | 0.931 | 0.799 | 84.1 | 9.25 | 4.59 | 17.9 | 2.9 | 0.84 | 1 | 57.2 | 0.68 | 0.5 | 348.2 | 0.215 | 5 | 87.03% |
| Nicaragua | 1905.26 | 5569.72 | 142 | 22 | 0.66 | 0.804 | 57.2 | 3.6 | 6.08 | 77.1 | 5.6 | 0.34 | 5 | 44.1 | 0.52 | 7.6 | 12.24 | 0.0655 | 142 | 26.53% |
| Nigeria | 2097.09 | 5186.41 | 131 | 25 | 0.539 | 0.635 | 57.2 | 4.1 | 9.71 | 97.3 | 7.9 | 0.28 | 5 | 40.9 | 0.77 | 8.4 | 6.45 | 0.062 | 153 | 25.10% |
| North Macedonia | 5917.26 | 16959.67 | 17 | 35 | 0.774 | 0.711 | 69.5 | 5.89 | 17.2 | 62.1 | 6.1 | 0.53 | 3 | 50.4 | 0.27 | 2.9 | 51.79 | 0.085 | 74 | 34.43% |
| Norway | 67329.68 | 62644.85 | 9 | 84 | 0.957 | 0.842 | 73.4 | 9.81 | 4.42 | 16.2 | 1.7 | 0.91 | 1 | 57.7 | 0.41 | 0.6 | 275.88 | 0.234 | 3 | 94.71% |
| Oman | 14485.39 | 31120.3 | 68 | 54 | 0.813 | 0.602 | 63.6 | 3 | 2.94 | 48 | 4.2 | 0.53 | 5 | 41.6 | 0.78 | 7.2 | 39.43 | 0.0965 | 59 | 39.05% |
| Pakistan | 1188.86 | 4812.89 | 108 | 31 | 0.557 | 0.564 | 54.8 | 4.31 | 4.3 | 92.1 | 7.1 | 0.38 | 5 | 36.1 | 0.67 | 7.1 | 5.26 | 0.065 | 143 | 26.33% |
| Panama | 12509.84 | 26782.48 | 86 | 35 | 0.815 | 0.73 | 67.2 | 7.18 | 12.85 | 46 | 2.7 | 0.51 | 2 | 46.3 | 0.6 | 3.5 | 43.98 | 0.0994 | 58 | 40.24% |
| Papua New Guinea | 2757.22 | 4286.63 | 120 | 27 | 0.555 | 0.635 | 58.4 | 6.1 | 2.6 | 82.3 | 6.7 | 0.31 | 3 | 42.9 | 0.66 | 6.9 | 6.71 | 0.0711 | 123 | 28.79% |
| Paraguay | 5001.07 | 13069.18 | 125 | 28 | 0.728 | 0.683 | 63 | 6.18 | 7.55 | 65.2 | 4.6 | 0.4 | 3 | 49.7 | 0.53 | 5.4 | 11.96 | 0.0793 | 93 | 32.12% |
| Peru | 6126.87 | 11878.52 | 76 | 38 | 0.777 | 0.714 | 67.9 | 6.53 | 7.18 | 67.6 | 3.3 | 0.45 | 3 | 49.9 | 0.56 | 3.4 | 17.02 | 0.0901 | 66 | 36.47% |
| Philippines | 3298.83 | 8389.85 | 95 | 34 | 0.718 | 0.781 | 64.5 | 6.56 | 2.52 | 81 | 4.6 | 0.51 | 4 | 44.7 | 0.55 | 7.5 | 15.29 | 0.0805 | 90 | 32.60% |
| Poland | 15720.99 | 34240.25 | 40 | 56 | 0.88 | 0.736 | 69.1 | 6.85 | 3.16 | 41 | 3.5 | 0.58 | 2 | 52.8 | 0.3 | 3.9 | 67.48 | 0.1212 | 38 | 49.08% |
| Portugal | 22176.3 | 34148.95 | 39 | 61 | 0.864 | 0.744 | 67 | 7.9 | 6.79 | 23.5 | 4 | 0.72 | 1 | 55 | 0.34 | 0.9 | 142.54 | 0.1426 | 27 | 57.73% |
| Puerto Rico | 32290.92 | 35277.23 | 65 | 28 | 0.742 | 0.7 | 53 | 6.32 | 8.89 | 64.7 | 5.1 | 0.44 | 3 | 44.5 | 0.31 | 5 | 78.7 | 0.0999 | 56 | 40.43% |
| Qatar | 50124.39 | 89968.77 | 77 | 63 | 0.848 | 0.629 | 72.3 | 3.24 | 0.21 | 43.7 | 1.2 | 0.69 | 5 | 40.9 | 0.62 | 6.1 | 146.73 | 0.1445 | 25 | 58.48% |
| Romania | 12896.09 | 32116.48 | 55 | 44 | 0.828 | 0.724 | 69.7 | 6.4 | 5.03 | 46.7 | 4.1 | 0.45 | 2 | 54.5 | 0.23 | 3.3 | 50.01 | 0.1075 | 44 | 43.51% |
| Russia | 10126.72 | 29812.21 | 29 | 30 | 0.824 | 0.706 | 61 | 3.31 | 5.59 | 72.6 | 4.9 | 0.5 | 6 | 49.9 | 0.27 | 8.8 | 27.16 | 0.0816 | 84 | 33.02% |
| Rwanda | 797.86 | 2213.83 | 38 | 54 | 0.543 | 0.791 | 70.9 | 3.1 | 1.49 | 86 | 5.9 | 0.57 | 6 | 41.6 | 0.77 | 6.3 | 4.19 | 0.0783 | 96 | 31.69% |
| Samoa | 4067.84 | 6768.75 | 98 | 43 | 0.715 | 0.678 | 62.1 | 5.72 | 9.15 | 63.3 | 6 | 0.61 | 2 | 45.1 | 0.43 | 3.6 | 6.71 | 0.0813 | 86 | 32.89% |
| Sao Tome and Principe | 2157.84 | 4273.84 | 170 | 47 | 0.625 | 0.642 | 56.2 | 4.1 | 15.75 | 70.3 | 7.9 | 0.36 | 2 | 45 | 0.65 | 3 | 4.03 | 0.0684 | 131 | 27.67% |
| Saudi Arabia | 20110.32 | 46759.66 | 63 | 53 | 0.854 | 0.599 | 62.4 | 2.08 | 7.45 | 68.8 | 3.9 | 0.53 | 7 | 44.1 | 0.59 | 8.9 | 68.7 | 0.0947 | 61 | 38.32% |
| Seychelles | 10764.42 | 25767.61 | 100 | 66 | 0.796 | 0.665 | 64.3 | 8.14 | 2.47 | 54.7 | 3.6 | 0.61 | 3 | 41.7 | 0.46 | 3.9 | 63.43 | 0.106 | 46 | 42.92% |
| Senegal | 1471.83 | 3502.82 | 123 | 45 | 0.512 | 0.684 | 58 | 5.67 | 3.62 | 74.6 | 6.9 | 0.5 | 3 | 43 | 0.8 | 5.1 | 4.7 | 0.0772 | 101 | 31.26% |
| Serbia | 7720.51 | 19107.2 | 44 | 38 | 0.806 | 0.736 | 66 | 6.22 | 9.01 | 66.1 | 5.9 | 0.5 | 3 | 50.7 | 0.21 | 3.8 | 31.71 | 0.0876 | 67 | 35.46% |
| Sierra Leone | 509.38 | 1727.11 | 163 | 33 | 0.452 | 0.668 | 48 | 4.86 | 5.2 | 84.4 | 8.3 | 0.28 | 3 | 43.4 | 0.68 | 5.2 | 995 | 0.1259 | 35 | 50.96% |
| Singapore | 59797.75 | 98520.03 | 2 | 85 | 0.938 | 0.724 | 89.4 | 6.03 | 4.1 | 26.3 | 1.1 | 1 | 4 | 50.3 | 0.25 | 4.3 | 333 | 0.1884 | 14 | 76.29% |
| Slovak Republic | 19266.51 | 31356.46 | 45 | 49 | 0.86 | 0.718 | 66.8 | 6.97 | 6.69 | 38.2 | 3.7 | 0.61 | 2 | 54.9 | 0.32 | 2.4 | 68.06 | 0.1187 | 39 | 48.04% |
| Slovenia | 25517.33 | 39768.63 | 37 | 60 | 0.917 | 0.743 | 67.8 | 7.54 | 4.97 | 25.8 | 2.9 | 0.75 | 1 | 55.9 | 0.41 | 1.2 | 120.17 | 0.1547 | 22 | 62.62% |
| Solomon Islands | 2250.6 | 2619.06 | 136 | 42 | 0.567 | 0.678 | 52.9 | 6.1 | 0.92 | 79.7 | 6.8 | 0.3 | 2 | 44.6 | 0.76 | 4.3 | 15.71 | 0.0782 | 97 | 31.64% |
| South Africa | 5655.87 | 13360.57 | 84 | 44 | 0.709 | 0.78 | 58.8 | 7.05 | 29.22 | 70.1 | 7.5 | 0.56 | 2 | 39.9 | 0.54 | 3.9 | 20.31 | 0.0764 | 109 | 30.94% |
| Spain | 27063.19 | 37765.8 | 31 | 62 | 0.904 | 0.795 | 66.9 | 8.12 | 15.53 | 40.4 | 4.3 | 0.69 | 1 | 51.8 | 0.39 | 0.9 | 227.12 | 0.133 | 31 | 53.85% |
| Sri Lanka | 3680.67 | 13224.61 | 99 | 38 | 0.782 | 0.68 | 57.4 | 6.14 | 5.88 | 81.8 | 5.2 | 0.48 | 4 | 46.3 | 0.4 | 8.1 | 23.83 | 0.0765 | 107 | 30.97% |
| St Kitts and Nevis | 18437.75 | 25653.32 | 139 | 55 | 0.779 | 0.724 | 62 | 6.32 | 8.9 | 64.7 | 5.1 | 0.65 | 6 | 42.2 | 0.43 | 9 | 21.04 | 0.0869 | 70 | 35.18% |
| St Lucia | 8804.56 | 12709.83 | 93 | 56 | 0.759 | 0.724 | 68.2 | 6.32 | 16.89 | 64.7 | 5.1 | 0.53 | 1 | 44.8 | 0.39 | 9 | 21.04 | 0.0815 | 85 | 32.98% |
| Sudan | 486.42 | 4142.45 | 171 | 16 | 0.51 | 0.629 | 45 | 2.54 | 19.65 | 104.8 | 8.1 | 0.18 | 6 | 39 | 0.74 | 8.9 | 1.01 | 0.0507 | 172 | 20.52% |
| Suriname | 4916.61 | 16734.75 | 162 | 38 | 0.738 | 0.707 | 49.5 | 6.82 | 9.78 | 60.1 | 6.7 | 0.38 | 3 | 46.5 | 0.47 | 4 | 5.64 | 0.0765 | 108 | 30.96% |
| Sweden | 52274.41 | 55037.72 | 10 | 85 | 0.945 | 0.82 | 74.9 | 9.26 | 8.29 | 18.2 | 1.5 | 0.87 | 1 | 62.1 | 0.44 | 0.6 | 336.17 | 0.2067 | 9 | 83.69% |
| Switzerland | 87097.04 | 71742.52 | 36 | 85 | 0.955 | 0.779 | 82 | 8.83 | 4.82 | 17.1 | 1.6 | 0.93 | 1 | 59.4 | 0.44 | 1.1 | 673.96 | 0.247 | **1** | 100% |
| Tajikistan | 859.14 | 3858.2 | 106 | 25 | 0.668 | 0.626 | 52.2 | 1.94 | 7.58 | 75.5 | 6.4 | 0.34 | 6 | 44.8 | 0.72 | 8.3 | 4.39 | 0.0622 | 152 | 25.19% |
| Tanzania | 1076.47 | 2779.91 | 141 | 38 | 0.529 | 0.713 | 61.7 | 5.1 | 2.53 | 78.1 | 5.9 | 0.33 | 5 | 43.4 | 0.84 | 6.1 | 3.65 | 0.0715 | 122 | 28.96% |
| Thailand | 7186.87 | 18232.8 | 21 | 36 | 0.777 | 0.708 | 69.4 | 6.04 | 1.1 | 70.8 | 3.3 | 0.56 | 4 | 47.6 | 0.35 | 7.8 | 25.29 | 0.0921 | 62 | 37.27% |
| Timor-Leste | 1442.73 | 4141.29 | 181 | 40 | 0.606 | 0.662 | 45.9 | 7.06 | 4.91 | 82.7 | 4.3 | 0.32 | 4 | 48.9 | 0.66 | 4.4 | 5.19 | 0.0723 | 118 | 29.29% |
| Togo | 914.95 | 2223.5 | 97 | 29 | 0.515 | 0.615 | 54.1 | 2.8 | 3.94 | 85.8 | 6.7 | 0.35 | 4 | 41.5 | 0.74 | 6.9 | 1.48 | 0.0656 | 140 | 26.57% |
| Trinidad and Tobago | 15425.64 | 25023.7 | 105 | 40 | 0.796 | 0.756 | 58.3 | 7.16 | 4.57 | 51.9 | 4 | 0.54 | 2 | 40.8 | 0.36 | 3.5 | 44.18 | 0.1006 | 55 | 40.73% |
| Tunisia | 3521.59 | 10822.85 | 78 | 44 | 0.74 | 0.644 | 55.8 | 6.59 | 16.59 | 68.1 | 7.2 | 0.45 | 3 | 41.9 | 0.5 | 5.6 | 17.55 | 0.0746 | 113 | 30.20% |
| Turkey | 8536.43 | 27235.43 | 34 | 40 | 0.82 | 0.635 | 64.4 | 4.48 | 13.11 | 79.1 | 4.6 | 0.49 | 6 | 46.3 | 0.5 | 8.3 | 27.47 | 0.0809 | 88 | 32.77% |
| Uganda | 822.03 | 2294.34 | 116 | 27 | 0.544 | 0.717 | 59.5 | 4.94 | 2.77 | 92.8 | 6 | 0.37 | 5 | 37.6 | 0.9 | 7.7 | 1.99 | 0.0689 | 129 | 27.88% |
| Ukraine | 3724.94 | 13054.76 | 64 | 33 | 0.779 | 0.721 | 54.9 | 5.81 | 9.13 | 69 | 6.2 | 0.42 | 3 | 46.7 | 0.2 | 6.3 | 13.1 | 0.0749 | 112 | 30.32% |
| United Arab Emirates | 36284.56 | 66771.5 | 16 | 71 | 0.89 | 0.655 | 76.2 | 2.7 | 3.19 | 38.1 | 1.9 | 0.78 | 6 | 44.5 | 0.53 | 7.3 | 115.48 | 0.1301 | 34 | 52.67% |
| United Kingdom | 41059.17 | 46482.86 | 8 | 77 | 0.932 | 0.767 | 79.3 | 8.54 | 4.47 | 38.3 | 4.4 | 0.79 | 1 | 56.1 | 0.41 | 1.8 | 290.75 | 0.1733 | 18 | 70.17% |
| United States | 63593.44 | 63593.44 | 6 | 67 | 0.926 | 0.724 | 76.6 | 7.92 | 8.05 | 38.3 | 1.8 | 0.78 | 1 | 51.7 | 0.37 | 3.9 | 505.42 | 0.1897 | 13 | 76.81% |
| Uruguay | 15438.41 | 22793.69 | 101 | 71 | 0.817 | 0.737 | 69.1 | 8.61 | 10.35 | 33.4 | 3.7 | 0.66 | 1 | 52 | 0.37 | 2.7 | 60.91 | 0.1114 | 41 | 45.09% |
| Uzbekistan | 1750.7 | 7734.02 | 69 | 26 | 0.72 | 0.626 | 57.2 | 2.12 | 7.04 | 73.1 | 5.5 | 0.39 | 6 | 48.4 | 0.65 | 7.6 | 7.82 | 0.0677 | 132 | 27.41% |
| Venezuela | 16055.6 | 17527.7 | 188 | 15 | 0.711 | 0.713 | 25.2 | 2.76 | 6.63 | 91.2 | 8.7 | 0.11 | 6 | 44.8 | 0.25 | 9 | 21.04 | 0.0597 | 161 | 24.17% |
| Vietnam | 2785.72 | 8650.13 | 70 | 36 | 0.704 | 0.7 | 58.8 | 2.94 | 2.39 | 63.9 | 4 | 0.54 | 5 | 45.8 | 0.47 | 7.7 | 14.08 | 0.0769 | 105 | 31.13% |
| Zambia | 985.13 | 3457.61 | 85 | 33 | 0.584 | 0.731 | 53.5 | 4.86 | 12.85 | 84.5 | 7.8 | 0.33 | 4 | 41.1 | 0.83 | 7.1 | 3.07 | 0.0669 | 135 | 27.10% |
| Zimbabwe | 1214.51 | 3537.35 | 140 | 24 | 0.571 | 0.73 | 43.1 | 3.16 | 5.35 | 99.2 | 8.6 | 0.23 | 5 | 42.6 | 0.57 | 8.3 | 7.13 | 0.058 | 164 | 23.48% |

**Table S2.** **The 12 indicators of the three dimensions of sustainability analyzed in this research.**

| **Compared countries** | **Indicator, its weight and measuring unit**  Plus (+) or minus (-) indicates that a greater (lesser) criterion value corresponds to a greater significance by country | | | | | | | | | | | |
| --- | --- | --- | --- | --- | --- | --- | --- | --- | --- | --- | --- | --- |
|  | **Environmental performance index**  **(1, score, +)** | **Ecological footprint per capita**  **(1, in global hectares, +)** | **Environmental health**  **(1, score, +)** | **Air quality**  **(1, score, +)** | **PM_2.5_ exposure**  **(1, score, +)** | **Climate change**  **(1, score, +)** | **The gross national income per capita**  **(1, current us$, +)** | **Healthy life expectancy**  **(1, years, +)** | **Life expectancy at birth**  **(1, years, +)** | **Death rates from air pollution**  **(1, %, -)** | **Happiness index**  **(1, score, +)** | **Positive peace index**  **(1, score, -)** |
|  | **V20** | **V21** | **V22** | **V23** | **V24** | **V25** | **V26** | **V27** | **V28** | **V29** | **V30** | **V31** |
| Afghanistan | 25.5 | 0.5 | 20.0 | 17.7 | 25.4 | 22.2 | 500 | 53.9 | 64.83 | 16.61 | 2.52 | 4.10 |
| Albania | 49.0 | 1.4 | 44.5 | 41.2 | 44.2 | 56.8 | 5210 | 69.1 | 78.57 | 9.54 | 5.12 | 2.93 |
| Algeria | 44.8 | 1.7 | 50.4 | 45.3 | 21.9 | 52.5 | 3570 | 66.4 | 76.88 | 10.73 | 4.89 | 3.39 |
| Angola | 29.7 | 0.7 | 20.4 | 26.8 | 32.6 | 49.0 | 2140 | 54.8 | 61.15 | 9.52 | - | 3.84 |
| Antigua and Barbuda | 48.5 | 1.9 | 55.5 | 57.5 | 44.6 | 58.5 | 13750 | 67 | 77.02 | 4.89 | - | - |
| Argentina | 52.2 | 5.1 | 60.2 | 56.9 | 54.7 | 60.2 | 9070 | 67.1 | 76.67 | 3.91 | 5.93 | 2.63 |
| Armenia | 52.3 | 1.3 | 43.5 | 36.3 | 22.1 | 46.7 | 4220 | 67.1 | 75.09 | 11.37 | 5.28 | 2.97 |
| Australia | 74.9 | 10.8 | 91.6 | 98.2 | 100.0 | 70.4 | 53680 | 70.9 | 82.90 | 1.11 | 7.18 | 1.39 |
| Austria | 79.6 | 4.6 | 88.0 | 81.3 | 73.9 | 71.3 | 48360 | 70.9 | 81.79 | 3.22 | 7.27 | 1.57 |
| Azerbaijan | 46.5 | 1.8 | 32.7 | 24.9 | 12.7 | 48.6 | 4480 | 63.6 | 73.01 | 10.87 | 5.17 | 3.59 |
| Bahrain | 51.0 | 7.3 | 49.2 | 41.2 | 16.5 | 67.1 | 19900 | 65.9 | 77.29 | 14.67 | 6.65 | 3.11 |
| Bangladesh | 29.0 | 0.6 | 22.4 | 20.2 | 24.2 | 35.6 | 2030 | 64.3 | 72.59 | 20.33 | 5.03 | 3.81 |
| Barbados | 45.6 | 1.9 | 60.7 | 66.0 | 40.7 | 59.1 | 14350 | 67 | 79.19 | 5.46 | - | - |
| Belarus | 53.0 | 5.1 | 55.9 | 49.9 | 34.1 | 53.1 | 6360 | 66 | 74.23 | 6.78 | 5.53 | 3.08 |
| Belgium | 73.3 | 3.7 | 86.0 | 80.7 | 69.8 | 70.2 | 45800 | 70.6 | 81.75 | 3.31 | 6.83 | 1.57 |
| Belize | 41.9 | 6.5 | 40.3 | 39.4 | 40.2 | 37.7 | 4110 | 65.3 | 74.62 | 6.79 | - | - |
| Benin | 30.0 | 1.2 | 20.3 | 25.6 | 37.5 | 30.7 | 1280 | 55.5 | 61.77 | 15.17 | 5.05 | 3.26 |
| Bhutan | 39.3 | 4.3 | 29.8 | 24.4 | 30.6 | 24.7 | 2840 | 63.4 | 71.78 | 16.15 | - | 2.99 |
| Bolivia | 44.3 | 3.2 | 35.9 | 32.4 | 34.5 | 40.0 | 3180 | 63.3 | 71.51 | 8.56 | 5.72 | 3.28 |
| Bosnia and Herzegovina | 45.4 | 3.9 | 43.9 | 31.6 | 29.3 | 57.5 | 6080 | 67.2 | 77.40 | 12.36 | 5.81 | 3.19 |
| Botswana | 40.4 | 1.9 | 20.2 | 19.7 | 17.8 | 37.9 | 6640 | 53.9 | 69.59 | 8.91 | 3.47 | 2.69 |
| Brazil | 51.2 | 3.6 | 49.7 | 50.0 | 56.6 | 51.7 | 7850 | 65.4 | 75.88 | 4.28 | 6.33 | 2.98 |
| Bulgaria | 57.0 | 4.6 | 50.3 | 33.0 | 28.2 | 69.5 | 9630 | 66.3 | 74.91 | 8.50 | 5.27 | 3.44 |
| Burkina Faso | 38.3 | 1.2 | 19.6 | 28.9 | 44.7 | 31.9 | 770 | 54.9 | 61.58 | 15.19 | 4.83 | 4.09 |
| Burundi | 27.0 | 0.8 | 21.9 | 36.0 | 57.9 | 16.8 | 230 | 55.6 | 61.58 | 16.01 | 3.78 | - |
| Cabo Verde | 32.8 | 1.6 | 30.4 | 26.5 | 21.4 | 39.5 | 3060 | 64.8 | 72.98 | 12.44 | - | - |
| Cambodia | 33.6 | 1.3 | 30.5 | 30.1 | 43.2 | 30.9 | 1500 | 61.5 | 69.82 | 16.55 | 4.83 | 3.63 |
| Cameroon | 33.6 | 1.1 | 13.6 | 16.4 | 16.4 | 66.4 | 1520 | 54.5 | 59.29 | 12.74 | 5.14 | 4.01 |
| Canada | 71.0 | 11.7 | 91.7 | 94.8 | 94.8 | 65.7 | 43540 | 71.3 | 82.05 | 1.50 | 7.1 | 1.37 |
| Central African Republic | 36.9 | 1.2 | 12.2 | 22.0 | 36.8 | 45.4 | 500 | 46.4 | 53.28 | 13.73 | 3.48 | 4.43 |
| Chad | 26.7 | 1.6 | 14.9 | 26.8 | 42.4 | 17.4 | 630 | 52 | 54.24 | 16.06 | 4.36 | 4.37 |
| Chile | 55.3 | 4.8 | 63.4 | 56.1 | 53.0 | 54.3 | 13470 | 70 | 80.18 | 5.41 | 6.17 | 2.15 |
| China | 37.3 | 3.5 | 41.8 | 27.1 | 23.4 | 46.3 | 10550 | 68.5 | 76.91 | 16.70 | 5.34 | 3.00 |
| Cyprus | 64.8 | 2.1 | 81.5 | 73.1 | 58.8 | 63.1 | 26440 | 72.4 | 80.98 | 5.02 | 5.88 | 2.26 |
| Colombia | 52.9 | 1.6 | 55.0 | 49.1 | 50.1 | 55.4 | 5790 | 69 | 77.29 | 6.32 | 6.01 | 3.18 |
| Comoros | 32.1 | 0.9 | 27.3 | 37.7 | 54.7 | 41.7 | 1400 | 58.9 | 64.32 | 13.22 | 4.29 | - |
| Congo Dem Rep | 36.4 | 0.7 | 22.4 | 29.9 | 45.4 | 40.1 | 550 | 54.1 | 60.68 | 15.47 | 5.34 | 4.31 |
| Congo Rep | 30.8 | 0.9 | 18.1 | 20.0 | 20.0 | 37.1 | 1770 | 56.2 | 64.57 | 11.33 | 5.34 | 4.00 |
| Costa Rica | 52.5 | 1.8 | 60.5 | 56.2 | 57.4 | 54.8 | 11530 | 70 | 80.28 | 4.38 | 7.07 | 2.31 |
| Cote d'Ivoire | 25.8 | 1.6 | 19.4 | 20.3 | 25.9 | 20.2 | 2280 | 54.8 | 57.78 | 14.03 | 5.31 | 3.64 |
| Croatia | 63.1 | 3.1 | 61.2 | 50.8 | 46.4 | 70.0 | 14530 | 68.6 | 78.42 | 6.25 | 5.88 | 2.31 |
| Czech Republic | 71.0 | 5.9 | 68.3 | 58.8 | 49.1 | 76.3 | 22070 | 68.8 | 79.13 | 5.62 | 6.97 | 1.99 |
| Denmark | 82.5 | 4.4 | 91.7 | 85.5 | 78.8 | 95.0 | 63010 | 71 | 81.20 | 2.60 | 7.62 | 1.24 |
| Djibouti | 28.1 | 0.9 | 20.1 | 21.1 | 16.7 | 40.1 | 3310 | 58 | 67.11 | 9.86 | 4.18 | 3.75 |
| Dominican Republic | 46.3 | 1.3 | 36.1 | 37.4 | 34.8 | 56.1 | 7260 | 64 | 74.08 | 6.80 | 5.55 | 3.19 |
| Ecuador | 51.0 | 2.3 | 50.2 | 48.7 | 46.2 | 60.2 | 5530 | 68.5 | 77.01 | 5.24 | 5.76 | 3.22 |
| Egypt | 43.3 | 1.3 | 33.8 | 35.3 | 0.0 | 55.0 | 3000 | 63 | 71.99 | 15.60 | 4.28 | 3.60 |
| El Salvador | 43.1 | 1.2 | 42.5 | 42.6 | 44.8 | 62.8 | 3630 | 64.9 | 73.32 | 6.48 | 6.06 | 3.28 |
| Equatorial Guinea | 38.1 | 2.5 | 27.6 | 24.6 | 17.6 | 46.1 | 5810 | 53.9 | 58.74 | 8.68 | 4.98 | 4.08 |
| Estonia | 65.3 | 10.6 | 73.0 | 80.3 | 95.7 | 59.0 | 23270 | 69.2 | 78.50 | 1.25 | 6.19 | 1.90 |
| Eswatini | 33.8 | 2.3 | 17.6 | 21.1 | 24.1 | 56.9 | 3410 | 50.1 | 60.19 | 8.80 | 4.31 | 3.66 |
| Ethiopia | 34.4 | 1.0 | 25.2 | 38.0 | 58.4 | 26.1 | 890 | 59.9 | 66.60 | 15.06 | 4.28 | 3.74 |
| Fiji | 34.4 | 1.9 | 34.7 | 30.0 | 31.5 | 36.9 | 4890 | 59.6 | 67.44 | 8.08 | - | - |
| Finland | 78.9 | 11.9 | 99.3 | 98.8 | 100.0 | 77.0 | 49800 | 71 | 81.79 | 0.73 | 7.84 | 1.26 |
| France | 80.0 | 3.6 | 91.5 | 88.1 | 82.2 | 81.9 | 39500 | 72.1 | 82.58 | 2.41 | 6.69 | 1.57 |
| Gabon | 45.8 | 1.6 | 27.9 | 28.2 | 16.1 | 63.8 | 7030 | 57.6 | 66.47 | 8.37 | 4.85 | 3.64 |
| Gambia | 27.9 | 0.5 | 21.4 | 23.9 | 32.7 | 35.8 | 750 | 57 | 62.05 | 16.48 | 5.05 | 3.55 |
| Georgia | 41.3 | 1.6 | 38.7 | 31.1 | 31.9 | 50.1 | 4260 | 64.7 | 73.77 | 9.60 | 4.89 | 2.77 |
| Germany | 77.2 | 4.3 | 89.6 | 81.1 | 70.4 | 71.5 | 47520 | 70.9 | 80.94 | 3.03 | 7.16 | 1.40 |
| Ghana | 27.6 | 1.9 | 20.1 | 18.0 | 17.1 | 29.6 | 2340 | 58 | 64.07 | 13.25 | 5.09 | 2.99 |
| Greece | 69.1 | 3.0 | 80.6 | 67.5 | 57.6 | 66.5 | 17950 | 70.9 | 81.94 | 4.89 | 5.72 | 2.23 |
| Grenada | 43.1 | 1.7 | 46.3 | 42.9 | 31.3 | 59.0 | 9410 | 63.9 | 72.40 | 6.48 | - | - |
| Guatemala | 31.8 | 1.5 | 30.8 | 34.3 | 41.2 | 36.7 | 4490 | 62.3 | 74.30 | 10.38 | 6.44 | 3.65 |
| Guyana | 35.9 | 3.0 | 33.5 | 31.9 | 20.6 | 38.2 | 7130 | 57.2 | 69.91 | 7.05 | - | 3.29 |
| Guinea | 26.4 | 1.6 | 18.6 | 24.7 | 37.8 | 26.8 | 1020 | 53.3 | 61.60 | 16.02 | 4.98 | 3.91 |
| Guinea-Bissau | 29.1 | 1.5 | 15.1 | 22.1 | 32.8 | 33.6 | 760 | 52.6 | 58.32 | 16.43 | 4.98 | 3.94 |
| Haiti | 27.0 | 0.4 | 21.8 | 31.1 | 47.5 | 37.0 | 1320 | 55.8 | 64.00 | 15.83 | 3.62 | 4.04 |
| Honduras | 37.8 | 1.4 | 33.4 | 35.4 | 46.9 | 43.1 | 2180 | 63 | 75.27 | 11.51 | 5.92 | 3.57 |
| Hong Kong SAR China | - | - | - | - | - | - | 48630 | - | 85.08 | - | 5.48 | - |
| Hungary | 63.7 | 3.9 | 54.1 | 42.8 | 43.0 | 71.3 | 15890 | 67.2 | 76.02 | 6.55 | 5.99 | 2.36 |
| Iceland | 72.3 | - | 98.1 | 98.1 | 100.0 | 60.8 | 62410 | 72 | 82.56 | 0.90 | 7.55 | 1.54 |
| Yemen | - | 0.3 | - | - | - | - | - | 57.5 | 66.13 | 14.39 | 3.66 | 4.54 |
| India | 27.6 | 1.2 | 16.3 | 13.4 | 10.9 | 45.0 | 1920 | 60.3 | 69.66 | 18.10 | 3.82 | 3.22 |
| Indonesia | 37.8 | 1.9 | 29.0 | 26.8 | 28.6 | 54.4 | 3870 | 62.8 | 71.72 | 10.37 | 5.35 | 3.22 |
| Iran, Islamic Rep. | 48.0 | 3.3 | 48.3 | 49.2 | 26.5 | 55.0 | 2960 | 66.3 | 76.68 | 11.02 | 4.72 | 3.57 |
| Iraq | 39.5 | 1.3 | 39.5 | 36.3 | 7.3 | 31.6 | 4680 | 62.7 | 70.60 | 15.00 | 4.85 | 4.10 |
| Ireland | 72.8 | 4.7 | 94.2 | 94.0 | 91.0 | 66.6 | 65750 | 71.1 | 82.30 | 1.68 | 7.09 | 1.43 |
| Israel | 65.8 | 2.7 | 83.6 | 76.5 | 62.6 | 56.9 | 42610 | 72.4 | 82.80 | 5.12 | 7.16 | 2.23 |
| Italy | 71.0 | 2.4 | 85.5 | 75.9 | 68.5 | 68.1 | 32360 | 71.9 | 83.20 | 4.27 | 6.48 | 1.98 |
| Jamaica | 48.2 | 1.3 | 45.5 | 43.6 | 43.7 | 65.7 | 4670 | 66.6 | 74.48 | 6.02 | 6.31 | 2.83 |
| Japan | 75.1 | 3.6 | 90.3 | 85.9 | 78.0 | 69.5 | 42330 | 74.1 | 84.36 | 3.06 | 5.94 | 1.47 |
| Jordan | 53.4 | 1.0 | 58.6 | 58.0 | 29.1 | 58.4 | 4310 | 67.6 | 74.53 | 9.81 | 4.4 | 3.19 |
| Kazakhstan | 44.7 | 5.9 | 40.8 | 32.0 | 26.6 | 44.6 | 8710 | 65 | 73.18 | 8.67 | 6.15 | 3.12 |
| Kenya | 34.7 | 0.8 | 25.7 | 34.9 | 49.9 | 49.0 | 1840 | 57.7 | 66.70 | 10.93 | 4.61 | 3.54 |
| Kyrgyz Republic | 39.8 | 1.3 | 33.7 | 27.0 | 27.9 | 42.5 | 1170 | 65.8 | 71.60 | 12.57 | 5.74 | 3.43 |
| Korea, Rep. | 66.5 | 4.9 | 81.4 | 71.7 | 53.8 | 62.6 | 32930 | 73.1 | 83.23 | 7.03 | 5.85 | 1.69 |
| Kosovo | - | - | - | - | - | - | 4440 | - | 72.50 | - | 6.37 | 3.14 |
| Kuwait | 53.6 | 7.9 | 57.3 | 55.0 | 24.2 | 49.7 | 36290 | 70.1 | 75.49 | 14.89 | 6.11 | 2.66 |
| Lao PDR | 34.8 | 2.3 | 27.2 | 27.8 | 41.3 | 30.7 | 2520 | 60.5 | 67.92 | 18.42 | 5.03 | 3.66 |
| Latvia | 61.6 | 7.6 | 58.0 | 54.8 | 53.2 | 67.7 | 17890 | 66.2 | 75.24 | 4.24 | 6.03 | 2.14 |
| Lebanon | 45.4 | 1.6 | 53.1 | 47.8 | 25.0 | 56.8 | 5370 | 66 | 78.93 | 9.84 | 4.58 | 3.55 |
| Lesotho | 28.0 | 1.3 | 11.8 | 15.3 | 18.8 | 52.4 | 1100 | 44.2 | 54.33 | 9.10 | 3.51 | 3.48 |
| Liberia | 22.6 | 1.1 | 21.3 | 30.9 | 43.7 | 24.9 | 570 | 54.9 | 64.10 | 13.62 | 4.63 | 3.72 |
| Libya | - | 2.9 | - | - | - | - | 4960 | 65.2 | 72.91 | 11.03 | 5.41 | 4.10 |
| Lithuania | 62.9 | 5.4 | 63.2 | 62.7 | 60.5 | 65.9 | 19620 | 66.7 | 76.13 | 3.28 | 6.26 | 2.01 |
| Luxembourg | 82.3 | 6.3 | 92.6 | 87.2 | 81.4 | 77.5 | 81110 | 71.6 | 82.45 | 2.46 | 7.32 | - |
| Macao SAR China | - | - | - | - | - | - | 75690 | - | 84.24 | - | 5.34 | - |
| Madagascar | 26.5 | 0.9 | 22.1 | 36.1 | 56.4 | 39.9 | 470 | 57.3 | 67.04 | 16.66 | 4.21 | 3.66 |
| Malaysia | 47.9 | 4.2 | 55.4 | 50.3 | 36.5 | 52.8 | 10570 | 65.7 | 76.16 | 6.12 | 5.38 | 2.59 |
| Malawi | 38.3 | 0.8 | 26.5 | 39.6 | 61.2 | 34.2 | 580 | 57.1 | 64.26 | 13.11 | 3.6 | 3.63 |
| Maldives | 35.6 | - | 48.0 | 51.2 | 61.3 | 27.7 | 6490 | 70 | 78.92 | 5.56 | 5.2 | - |
| Mali | 29.4 | 1.6 | 19.5 | 29.2 | 45.6 | 26.3 | 830 | 54.6 | 59.31 | 14.18 | 4.72 | 3.84 |
| Malta | 70.7 | 1.4 | 86.1 | 77.6 | 66.6 | 62.6 | 25860 | 71.5 | 82.60 | 4.17 | 6.6 | - |
| Mauritania | 27.7 | 2.5 | 20.0 | 22.4 | 22.0 | 32.6 | 1670 | 59.8 | 64.93 | 13.31 | 4.23 | 3.88 |
| Mauritius | 45.1 | 1.8 | 60.0 | 51.1 | 37.8 | 51.0 | 10230 | 63.9 | 74.24 | 5.58 | 6.05 | 2.57 |
| Mexico | 52.6 | 2.1 | 47.5 | 40.8 | 42.2 | 65.2 | 8480 | 65.8 | 75.05 | 6.65 | 6.32 | 3.12 |
| Myanmar | 25.1 | 1.7 | 24.6 | 20.7 | 27.0 | 22.2 | 1350 | 60.9 | 67.13 | 18.16 | 4.43 | 3.72 |
| Moldova | 44.4 | 2.0 | 45.6 | 44.3 | 41.7 | 52.2 | 4560 | 64.5 | 71.90 | 5.72 | 5.77 | 3.11 |
| Mongolia | 32.2 | 8.1 | 27.6 | 17.4 | 11.2 | 16.6 | 3740 | 60.3 | 69.87 | 13.07 | 5.68 | 2.92 |
| Montenegro | 46.3 | 2.4 | 46.7 | 33.9 | 31.2 | 65.6 | 7900 | 67 | 76.88 | 10.33 | 5.58 | 2.79 |
| Morocco | 42.3 | 1.4 | 33.3 | 27.0 | 14.0 | 48.7 | 3020 | 63.7 | 76.68 | 12.38 | 4.92 | 3.18 |
| Mozambique | 33.9 | 0.7 | 28.1 | 40.7 | 64.5 | 31.1 | 460 | 50.4 | 60.85 | 12.58 | 4.79 | 3.70 |
| Namibia | 40.2 | 4.4 | 22.5 | 24.4 | 27.2 | 55.4 | 4500 | 56.1 | 63.71 | 9.47 | 4.57 | 3.08 |
| Nepal | 32.7 | 0.9 | 21.0 | 14.6 | 16.3 | 23.7 | 1190 | 61.3 | 70.78 | 23.13 | 5.269 | 3.70 |
| Netherlands | 75.3 | 3.6 | 91.0 | 82.4 | 72.8 | 65.8 | 51070 | 71.4 | 82.01 | 3.13 | 7.46 | 1.36 |
| New Zealand | 71.3 | 12.1 | 88.0 | 97.4 | 100.0 | 61.5 | 42870 | 70.2 | 81.71 | 0.97 | 7.28 | 1.45 |
| Nicaragua | 39.2 | 1.7 | 40.2 | 41.7 | 53.5 | 38.0 | 1850 | 65.5 | 74.49 | 9.97 | 5.97 | 3.57 |
| Nigeria | 31.0 | 1.0 | 13.9 | 18.0 | 21.4 | 50.8 | 2000 | 54.4 | 54.69 | 12.56 | 4.76 | 3.84 |
| North Macedonia | 55.4 | 2.0 | 43.6 | 26.5 | 18.3 | 75.2 | 5750 | 66.1 | 75.80 | 12.74 | 5.1 | 2.87 |
| Norway | 77.7 | 10.3 | 98.5 | 97.9 | 100.0 | 78.9 | 78290 | 71.4 | 82.91 | 1.10 | 7.39 | 1.27 |
| Oman | 38.5 | 6.5 | 43.4 | 36.4 | 11.7 | 32.0 | 15030 | 64.7 | 77.86 | 14.16 | - | 2.87 |
| Pakistan | 33.1 | 0.7 | 14.6 | 9.9 | 8.5 | 50.6 | 1270 | 56.9 | 67.27 | 16.88 | 4.93 | 3.74 |
| Panama | 47.3 | 2.2 | 50.4 | 56.0 | 61.7 | 50.3 | 12420 | 68.7 | 78.51 | 4.18 | 6.18 | 2.72 |
| Papua New Guinea | 32.4 | 2.3 | 28.4 | 33.9 | 49.4 | 47.6 | 2720 | 57.1 | 64.50 | 20.03 | - | 3.71 |
| Paraguay | 46.4 | 5.6 | 46.8 | 47.0 | 59.0 | 40.9 | 5180 | 65.8 | 74.25 | 6.91 | 5.65 | 3.24 |
| Peru | 44.0 | 2.1 | 45.1 | 46.0 | 47.6 | 41.3 | 6030 | 69.5 | 76.74 | 7.54 | 5.84 | 3.00 |
| Philippines | 38.4 | 1.1 | 34.1 | 30.4 | 34.2 | 42.8 | 3430 | 62 | 71.23 | 12.10 | 5.88 | 3.41 |
| Poland | 60.9 | 4.7 | 58.9 | 44.7 | 40.7 | 65.4 | 15260 | 68.7 | 77.86 | 7.43 | 6.17 | 2.14 |
| Portugal | 67.0 | 3.2 | 83.4 | 84.4 | 86.4 | 63.3 | 21810 | 71 | 80.68 | 2.00 | 5.93 | 1.70 |
| Puerto Rico | - | - | - | - | - | - | 21160 | - | 79.93 | 1.30 | - | - |
| Qatar | 37.1 | 11.9 | 56.9 | 48.6 | 13.0 | 12.1 | 55990 | 67.1 | 80.23 | 14.97 | 6.37 | 2.69 |
| Romania | 64.7 | 3.7 | 50.0 | 43.6 | 41.1 | 84.6 | 12600 | 66.8 | 75.46 | 6.32 | 6.14 | 2.53 |
| Russia | 50.5 | 6.4 | 53.0 | 54.1 | 46.9 | 59.9 | 10690 | 64.2 | 73.08 | 4.11 | 5.48 | 3.09 |
| Rwanda | 33.8 | 0.6 | 24.4 | 30.9 | 45.2 | 35.9 | 780 | 60.2 | 69.02 | 15.07 | 3.42 | 3.61 |
| Samoa | 37.3 | 1.5 | 42.4 | 33.4 | 46.3 | 35.0 | 4050 | 62.1 | 73.32 | 13.33 | - | - |
| Sao Tome and Principe | 37.6 | 1.0 | 28.7 | 24.6 | 30.8 | 35.0 | 2060 | 61.6 | 70.39 | 14.90 | - | - |
| Saudi Arabia | 44.0 | 5.8 | 47.2 | 37.4 | 10.0 | 37.5 | 21930 | 64 | 75.13 | 14.75 | 6.49 | 3.16 |
| Seychelles | 58.2 | - | 50.8 | 53.6 | 39.2 | 70.7 | 12200 | 64 | 73.94 | 4.41 | - | - |
| Senegal | 30.7 | 1.2 | 20.4 | 25.4 | 33.8 | 30.3 | 1430 | 59.4 | 67.94 | 14.77 | 5.13 | 3.16 |
| Serbia | 55.2 | 3.4 | 47.8 | 33.6 | 28.1 | 68.1 | 7430 | 66.9 | 75.69 | 10.14 | 6.08 | 2.96 |
| Sierra Leone | 25.7 | 1.0 | 19.1 | 25.1 | 37.3 | 24.9 | 510 | 52.9 | 54.70 | 15.47 | 3.85 | 3.58 |
| Singapore | 58.1 | 3.2 | 85.0 | 76.9 | 61.8 | 40.2 | 54920 | 73.6 | 83.50 | 5.80 | 6.38 | 1.56 |
| Slovak Republic | 68.3 | 4.5 | 64.3 | 56.2 | 43.1 | 71.9 | 18920 | 68.5 | 77.47 | 6.32 | 6.33 | 2.18 |
| Slovenia | 72.0 | 4.3 | 68.9 | 60.9 | 62.5 | 75.2 | 25340 | 70.7 | 81.28 | 4.44 | 6.46 | 1.86 |
| Solomon Islands | 26.7 | 6.7 | 20.4 | 27.0 | 40.1 | 45.0 | 2300 | 57.8 | 73.00 | 22.54 | - | - |
| South Africa | 43.1 | 3.4 | 31.1 | 28.9 | 22.7 | 62.8 | 6010 | 56.2 | 64.13 | 6.41 | 4.96 | 2.93 |
| Spain | 74.3 | 3.7 | 86.8 | 80.2 | 83.3 | 71.2 | 27360 | 72.1 | 83.49 | 2.69 | 6.49 | 1.83 |
| Sri Lanka | 39.0 | 1.0 | 42.1 | 37.8 | 44.8 | 31.0 | 3720 | 67 | 76.98 | 10.10 | 4.33 | 3.32 |
| St Kitts and Nevis | - | - | - | - | - | - | 19080 | - | - | 2.32 | - | - |
| St Lucia | 43.1 | 0.9 | 47.8 | 46.3 | 36.1 | 54.8 | 8560 | 64.7 | 76.20 | 5.91 | - | - |
| Sudan | 34.8 | 1.4 | 20.8 | 17.6 | 14.8 | 52.8 | 530 | 59.9 | 65.31 | 15.95 | 2.817 | 4.21 |
| Suriname | 45.2 | 3.5 | 36.6 | 37.4 | 30.3 | 52.8 | 4620 | 62.4 | 71.68 | 6.89 | - | - |
| Sweden | 78.7 | 7.0 | 98.4 | 98.2 | 100.0 | 77.2 | 54050 | 71.9 | 82.96 | 0.81 | 7.36 | 1.23 |
| Switzerland | 81.5 | 2.2 | 95.0 | 90.6 | 87.8 | 81.6 | 82620 | 72.5 | 83.70 | 2.31 | 7.57 | 1.28 |
| Tajikistan | 38.2 | 0.9 | 20.7 | 15.2 | 10.7 | 36.9 | 1060 | 62 | 71.10 | 15.94 | 5.47 | 3.96 |
| Tanzania | 31.1 | 1.2 | 27.4 | 35.8 | 53.0 | 18.7 | 1080 | 58.5 | 65.46 | 13.91 | 3.62 | 3.50 |
| Thailand | 45.4 | 2.5 | 48.4 | 40.6 | 41.1 | 52.5 | 7040 | 68.3 | 77.15 | 7.89 | 5.99 | 2.94 |
| Timor-Leste | 35.3 | 0.4 | 28.9 | 33.6 | 49.0 | 23.4 | 1990 | 60.9 | 69.50 | 16.66 | - | 3.59 |
| Togo | 29.5 | 0.8 | 16.4 | 24.1 | 33.7 | 34.4 | 920 | 56.2 | 61.04 | 14.80 | 4.11 | 3.64 |
| Trinidad and Tobago | 47.5 | 7.8 | 54.6 | 57.2 | 30.1 | 42.1 | 15420 | 66.2 | 73.51 | 7.24 | 6.19 | 2.68 |
| Tunisia | 46.7 | 1.5 | 49.2 | 48.7 | 26.2 | 60.2 | 3300 | 66.9 | 76.70 | 10.72 | 4.6 | 2.87 |
| Turkey | 42.6 | 2.8 | 51.3 | 49.5 | 32.3 | 49.3 | 9050 | 68.4 | 77.69 | 9.45 | 4.95 | 3.23 |
| Uganda | 35.6 | 1.0 | 25.7 | 32.6 | 48.0 | 27.4 | 800 | 58.2 | 63.37 | 13.58 | 4.64 | 3.85 |
| Ukraine | 49.5 | 3.8 | 49.0 | 39.8 | 30.6 | 69.2 | 3570 | 64.3 | 71.83 | 6.17 | 4.88 | 3.11 |
| United Arab Emirates | 55.6 | 6.6 | 55.2 | 48.6 | 13.9 | 38.9 | 39410 | 66 | 77.97 | 12.42 | 6.56 | 2.30 |
| United Kingdom | 81.3 | 2.7 | 91.7 | 84.7 | 75.4 | 90.0 | 39830 | 70.1 | 81.20 | 2.40 | 7.06 | 1.69 |
| United States | 69.3 | 7.8 | 82.8 | 84.2 | 78.8 | 71.4 | 64140 | 66.1 | 78.79 | 2.00 | 6.95 | 1.95 |
| Uruguay | 49.1 | 9.3 | 67.7 | 67.7 | 71.0 | 55.5 | 15790 | 67.5 | 77.91 | 2.41 | 6.43 | 2.08 |
| Uzbekistan | 44.3 | 1.8 | 29.7 | 15.0 | 0.3 | 65.9 | 1740 | 64.7 | 71.73 | 14.52 | 6.18 | 3.47 |
| Venezuela | 50.3 | 2.3 | 46.5 | 51.3 | 33.7 | 63.3 | - | 64.4 | 72.06 | 6.68 | 4.89 | 4.01 |
| Vietnam | 33.4 | 2.0 | 40.6 | 32.0 | 36.6 | 30.7 | 2650 | 65.3 | 75.40 | 11.26 | 5.41 | 3.08 |
| Zambia | 34.7 | 1.2 | 21.0 | 27.0 | 36.9 | 26.5 | 1160 | 54.4 | 63.89 | 11.25 | 4.07 | 3.59 |
| Zimbabwe | 37.0 | 1.0 | 22.5 | 27.2 | 38.2 | 37.0 | 1140 | 53.1 | 61.49 | 11.00 | 3.15 | 3.98 |

**Table S5. Descriptive statistics of 12 CSS Models and Country Success Model.**

| **Descriptive statistics** | **Descriptive statistics of 12 CSS Models** | | | | | | | | | | | | **Descriptive statistics of Country Success Model** |
| --- | --- | --- | --- | --- | --- | --- | --- | --- | --- | --- | --- | --- | --- |
|  | **Environmental performance index** | **Ecological footprint per capita** | **Environmental health** | **Air quality** | **PM_2.5_ exposure** | **Climate change** | **The gross national income per capita** | **Healthy life expectancy** | **Life expectancy at birth** | **Death rates from air pollution** | **Happiness index** | **Positive peace index** |  |
|  | **Model 1** | **Model 2** | **Model 3** | **Model 4** | **Model 5** | **Model 6** | **Model 7** | **Model 8** | **Model 9** | **Model 10** | **Model 11** | **Model 12** |  |
| Mean | 46.882 | 3.047 | 46.008 | 44.513 | 42.834 | 50.197 | 14088.070 | 63.633 | 72.835 | 9.521 | 5.497 | 2.996 | 0.098 |
| Median | 44.800 | 2.100 | 43.900 | 38.000 | 41.100 | 52.400 | 5210.000 | 65.500 | 74.490 | 9.810 | 5.530 | 3.120 | 0.081 |
| Maximum | 82.500 | 12.100 | 99.300 | 98.800 | 100.000 | 95.000 | 82620.000 | 74.100 | 85.080 | 23.130 | 7.840 | 4.540 | 0.247 |
| Minimum | 22.600 | 0.300 | 11.800 | 9.900 | 0.000 | 12.100 | 230.000 | 44.200 | 53.280 | 0.730 | 2.520 | 1.230 | 0.048 |
| Standard Deviation | 15.797 | 2.607 | 24.215 | 22.469 | 22.749 | 17.104 | 19031.507 | 6.175 | 7.517 | 5.169 | 1.096 | 0.841 | 0.045 |
| Skewness | 0.672 | 1.683 | 0.677 | 0.897 | 0.736 | 0.016 | 1.859 | -0.558 | -0.581 | 0.164 | -0.151 | -0.567 | 1.581 |
| Kurtosis | -0.577 | 2.616 | -0.582 | -0.107 | 0.172 | -0.672 | 2.710 | -0.321 | -0.389 | -0.836 | -0.329 | -0.637 | 1.744 |
| Observations | 166 | 165 | 166 | 166 | 166 | 166 | 171 | 168 | 172 | 170 | 153 | 152 | 173 |

**Table S6. Results of goodness-of-fit testing of CSS Models.**

| **Independent variables** | **Dependent variables** | | | | | | | | | | | |
| --- | --- | --- | --- | --- | --- | --- | --- | --- | --- | --- | --- | --- |
|  | **Environmental performance index** | **Ecological footprint per capita** | **Environmental health** | **Air quality** | **PM_2.5_ exposure** | **Climate change** | **The gross national income per capita** | **Healthy life expectancy** | **Life expectancy at birth** | **Death rates from air pollution** | **Happiness index** | **Positive peace index** |
|  | **1** | **2** | **3** | **4** | **5** | **6** | **7** | **8** | **9** | **10** | **11** | **12** |
| GDP per capita | 0.000313**  (3.401334) | -4.17E-05  (-1.356340) | 0.000535***  (4.421244) | 0.000633***  (4.022523) | 0.000662**  (3.266088) | 0.000512**  (3.018089) | 0.769491***  (12.164391) | 0.000086*  (2.034748) | 0.000091*  (1.843853) | -0.000065  (-1.425523) | 0.000019*  (2.212400) | -0.000003  (-1.446391) |
| GDP per capita in PPP | -0.000113  (-1.232984) | 5.92E-05*  (1.927313) | -0.000256*  (-2.119802) | -0.000302*  (-1.924982) | -0.000413*  (-2.044130) | -0.000315*  (-1.866043) | 0.056724  (0.913789) | -0.000124**  (-2.917199) | -0.000091*  (-1.837773) | 0.000021  (0.450517) | -0.000012  (-1.397361) | -5.0964E-7  (-0.218468) |
| Ease of doing business ranking | -0.033506  (-1.635500) | -0.010206  (-1.491343) | 0.018259  (0.676523) | 0.025747  (0.734341) | 0.050376  (0.266928) | -0.064331*  (-1.701287) | -14.592205  (-1.082856) | 0.019795*  (2.099990) | 0.012885  (1.168875) | -0.011152  (-1.151176) | 0.001973  (0.939057) | 0.000487  (0.882898) |
| Corruption perceptions index | -0.015624  (-0.212643) | 0.063151*  (2.554871) | 0.061246  (0.632719) | 0.209861*  (1.668918) | 0.012383  (0.076378) | -0.228411*  (-1.684246) | 191.4219***  (3.786962) | -0.046012  (-1.346438) | 0.004337  (0.108326) | 0.051486  (1.406269) | -0.008770  (-1.075428) | -0.007879***  (-3.814297) |
| Human development index | 72.86973***  (7.772084) | 1.353070  (0.442421) | 116.477833***  (9.429985) | 88.186809***  (5.495928) | 4.620816  (0.223349) | 80.490102***  (4.651197) | 6023.747051  (0.950978) | 42.27937***  (9.901039) | 47.88108***  (9.586709) | -17.50831***  (-3.814029) | 4.160927***  (4.701771) | -0.820717**  (-3.472641) |
| Global gender gap | -7.622021  (-0.716778) | 4.262253  (1.216885) | -1.691390  (-0.120736) | 30.230931*  (1.661166) | 86.03999***  (3.666820) | -6.242755  (-0.318070) | 7018.695623  (0.937217) | -6.765446  (-1.399910) | -6.161720  (-1.068592) | -14.347576**  (-2.750849) | 0.700383  (0.689389) | 0.218495  (0.829070) |
| Economic freedom | -0.068137  (-0.638495) | -0.034783  (-1.075958) | -0.068610  (-0.488016) | -0.078745  (-0.431165) | 0.296051  (1.257225) | 0.026282  (0.133433) | -77.948958  (-1.069325) | 0.057907  (1.280877) | 0.018549  (0.358625) | -0.002635  (-0.054686) | 0.013426  (1.460652) | 0.004804*  (1.993745) |
| Democracy index | 0.177808  (0.301060) | -0.128224  (-0.625575) | 0.694181  (0.373745) | 1.466157  (1.450539) | 2.263184*  (1.736587) | 0.973922  (0.893425) | 540.412919  (1.384244) | 0.094496  (0.343970) | 0.528355*  (1.670452) | -0.740428*  (-2.569328) | -0.075634  (-1.221014) | -0.019113  (-1.124115) |
| Unemployment rate | -0.072075  (-0.776565) | -0.026638  (-0.858031) | -0.318043*  (-2.601105) | -0.444612**  (-2.799130) | -0.641105**  (-3.130396) | 0.195622  (1.141942) | -111.106365*  (-1.717559) | -0.098268*  (-2.277354) | -0.100698*  (-1.985556) | -0.106757*  (-2.288541) | -0.019934*  (-2.200983) | 0.001175  (0.502139) |
| Fragile state index | 0.036333  (0.453116) | -0.062427*  (-2.309923) | 0.000408  (0.003858) | -0.057528  (-0.419212) | 0.063907  (0.361183) | -0.071809  (-0.485190) | -67.817785  (-1.221005) | 0.026503  (0.709622) | -0.002109  (-0.047506) | 0.064087  (1.603943) | -0.006592  (-0.823696) | 0.009465***  (4.711634) |
| Economic decline index | 1.513783*  (2.421470) | -0.307477  (-1.476436) | 1.300715  (1.579338) | 1.551390  (1.450052) | 1.212224  (0.878766) | 3.860704**  (3.345908) | 682.189230*  (1.778239) | -0.439529  (-1.522312) | 0.024320  (0.080474) | -0.599323*  (-1.951754) | -0.111347*  (-2.051300) | -0.015211  (-0.967641) |
| Government effectiveness | -3.572077  (-0.395841) | -8.415583**  (-2.895070) | 6.684906  (0.562307) | -7.631924  (-0.494176) | -1.895811  (-0.095207) | -5.472835  (-0.328583) | -5999.882216  (-0.977695) | 4.266230  (1.050562) | 3.042558  (0.628456) | -2.069336  (-0.480273) | -0.667023  (-0.745265) | -0.881574***  (-3.772195) |
| Civil liberties | 0.853266  (1.117724) | -0.116911  (-0.442826) | 0.951224  (0.945823) | 0.938268  (0.718164) | -0.167885  (-0.099663) | 1.124256  (0.797898) | 918.574232*  (1.823656) | 0.381581  (1.072550) | 0.499854  (1.209640) | -0.164668  (-0.443850) | -0.101047  (-1.201573) | 0.059851*  (2.606901) |
| The global sustainable competitiveness index | 0.448362**  (3.088986) | -0.007928  (-0.161237) | 0.087544  (0.457815) | -0.062574  (-0.251898) | 1.129099**  (3.525282) | 0.335036  (1.250578) | -8.331234  (-0.083455) | 0.020068  (0.297522) | -0.069050  (-0.870957) | -0.056593  (-0.783660) | 0.036244*  (2.472418) | -0.005632  (-1.464698) |
| Population growth | 3.593435  (1.046083) | 1.849928  (1.604780) | 0.212698  (0.047000) | 7.998834  (1.360599) | 15.192330*  (2.004267) | -8.005830  (-1.262684) | 2008.060614  (0.838260) | -2.068387  (-1.296975) | -2.247923  (-1.192405) | 0.857443  (0.505047) | 0.799758*  (2.370309) | 0.057850  (0.624680) |
| Human rights and rule of law index | -1.616841**  (-3.290957) | 0.149491  (0.911082) | -1.168762*  (-1.805753) | -0.611323  (-0.727065) | -0.886042  (-0.817305) | -1.163669  (-1.283265) | 490.085313  (1.498892) | -0.233618  (-1.022274) | 0.112250  (0.431687) | -0.226083  (-0.925285) | -0.003371  (-0.064288) | 0.001668  (0.120554) |
| Wealth per adult | 0.007826*  (2.216680) | -4.17E-05  (-0.330761) | 0.011449*  (2.461512) | 0.012544*  (2.075946) | 0.017499*  (2.246104) | 0.002420  (0.371374) | 3.516426  (1.421697) | -0.000174  (0.915723) | -0.002060  (-1.057438) | -0.000115  (-0.065136) | -0.000345  (-1.036342) | -0.000155  (-1.637966) |
| R^2^ | 0.885 | 0.534 | 0.915 | 0.834 | 0.730 | 0.666 | 0.960 | 0.836 | 0.841 | 0.726 | 0.792 | 0.977 |
| Adjusted R^2^ | 0.872 | 0.480 | 0.905 | 0.814 | 0.699 | 0.627 | 0.956 | 0.818 | 0.823 | 0.696 | 0.765 | 0.974 |
| F |  |  | 93.802*** | 43.593*** | 23.54515*** | 17.349*** | 218.514*** | 45.068*** | 47.807*** | 23.718*** | 30.184*** | 331.625*** |

Standardized beta coefficients: *significant at - p<0.1, **significant at p<0.01 and ***significant at α = p<0.001.

**Table S7. Standardized coefficients' beta values of the dependent variables.**

| **Independent variables** | | **Standardized coefficients' beta values of the dependent variables** | | | | | | | | | | | | **Average β_av_ values of independent variables in absolute values (criteria weights)** |
| --- | --- | --- | --- | --- | --- | --- | --- | --- | --- | --- | --- | --- | --- | --- |
|  |  | **Environmental performance index** | **Ecological footprint per capita** | **Environmental health** | **Air quality** | **PM_2.5_ exposure** | **Climate change** | **The gross national income per capita** | **Healthy life expectancy** | **Life expectancy at birth** | **Death rates from air pollution** | **Happiness index** | **Positive peace index** |  |
|  |  | **Model 1** | **Model 2** | **Model 3** | **Model 4** | **Model 5** | **Model 6** | **Model 7** | **Model 8** | **Model 9** | **Model 10** | **Model 11** | **Model 12** |  |
| 1 | GDP per capita | 0.385** | -0.308 | 0.430*** | 0.548*** | 0.566** | 0.582** | 0.789*** | 0.271* | 0.236* | -0.242 | 0.359* | -0.075 | 0.399 |
| 2 | GDP per capita in PPP | -0.152 | 0.481* | -0.224* | -0.285* | -0.385* | -0.391* | 0.064 | -0.426** | -0.258* | 0.085 | -0.248 | -0.012 | 0.251 |
| 3 | Ease of doing business ranking | -0.115 | -0.214 | 0.041 | 0.062 | 0.12 | -0.203* | -0.041 | 0.175* | 0.094 | -0.118 | 0.100 | 0.032 | 0.110 |
| 4 | Corruption perceptions index | -0.018 | 0.445* | 0.046 | 0.171* | 0.01 | -0.245* | 0.185*** | -0.138 | 0.011 | 0.183 | -0.153 | -0.175*** | 0.148 |
| 5 | Human development index | 0.689*** | 0.078 | 0.719*** | 0.586*** | 0.03 | 0.703*** | 0.047 | 1.026*** | 0.951*** | -0.505*** | 0.590*** | -0.149** | 0.506 |
| 6 | Global gender gap | -0.029 | 0.098 | -0.004 | 0.080* | 0.224*** | -0.022 | 0.022 | -0.067 | -0.050 | -0.169** | 0.04 | 0.016 | 0.068 |
| 7 | Economic freedom | -0.043 | -0.132 | -0.028 | -0.035 | 0.129 | 0.015 | -0.039 | 0.092 | 0.025 | -0.005 | 0.126 | 0.057* | 0.061 |
| 8 | Democracy index | 0.024 | -0.105 | 0.061 | 0.139 | 0.211* | 0.121 | 0.060 | 0.033 | 0.149* | -0.306* | -0.152 | -0.050 | 0.118 |
| 9 | Unemployment rate | -0.027 | -0.060 | -0.077* | -0.115** | -0.164** | 0.067 | -0.034* | -0.094* | -0.079* | -0.121* | -0.109* | 0.008 | 0.080 |
| 10 | Fragile state index | 0.054 | -0.568* | 0.000 | -0.060 | 0.066 | -0.099 | -0.083 | 0.102 | -0.007 | 0.293 | -0.148 | 0.274*** | 0.146 |
| 11 | Economic decline index | 0.186* | -0.233 | 0.104 | 0.134 | 0.104 | 0.438** | 0.069* | -0.14 | 0.006 | -0.227* | -0.207* | -0.036 | 0.157 |
| 12 | Government effectiveness | -0.046 | -0.672** | 0.056 | -0.069 | -0.017 | -0.065 | -0.064 | 0.144 | 0.084 | -0.083 | -0.132 | -0.221*** | 0.138 |
| 13 | Civil liberties | 0.095 | -0.080 | 0.069 | 0.074 | -0.013 | 0.116 | 0.085* | 0.110 | 0.118 | -0.057 | -0.167 | 0.126* | 0.093 |
| 14 | The global sustainable competitiveness index | 0.169** | -0.018 | 0.022 | -0.017 | 0.296** | 0.117 | -0.003 | 0.020 | -0.055 | -0.065 | 0.206* | -0.041 | 0.086 |
| 15 | Population growth | 0.045 | 0.141 | 0.002 | 0.070 | 0.132* | -0.093 | 0.021 | -0.066 | -0.059 | 0.033 | 0.148* | 0.014 | 0.069 |
| 16 | Human rights and rule of law index | -0.257** | 0.145 | -0.121* | -0.068 | -0.098 | -0.171 | 0.065 | -0.096 | 0.038 | -0.111 | -0.008 | 0.005 | 0.099 |
| 17 | Wealth per adult | 0.083 | -0.025 | 0.079* | 0.093* | 0.129* | 0.024 | 0.031 | -0.005 | -0.046 | -0.004 | -0.055 | -0.030 | 0.050 |

**Table S8. How countries’ success and its factors influence sustainability indicators.**

| **Environmental performance index** | **Ecological footprint per capita** | **Environmental health** | **Air quality** | **PM_2.5_ exposure** | **Climate change** | **The gross national income per capita** | **Healthy life expectancy** | **Life expectancy at birth** | **Death rates from air pollution** | **Happiness index** | **Positive peace index** |
| --- | --- | --- | --- | --- | --- | --- | --- | --- | --- | --- | --- |
| **Model 1** | **Model 2** | **Model 3** | **Model 4** | **Model 5** | **Model 6** | **Model 7** | **Model 8** | **Model 9** | **Model 10** | **Model 11** | **Model 12** |
| **When a nation’s success increases by 1%, the indicator improves by** | | | | | | | | | | | |
| 0.618% | 1.139% | 0.996% | 0.957% | 0.866% | 0.438% | 3.309% | 0.136% | 0.150% | -0.784% | 0.309% | -0.547% |
| **The 17 independent variables explain the dependent variable under analysis in** | | | | | | | | | | | |
| 89.5% | 53.5% | 91.5% | 83.4% | 73.0% | 66.6% | 94.5% | 83.6% | 84.1% | 72.6% | 79.2% | 97.7% |

**Table S9. The decision matrix to determine the most rational country success indicators to improve 12 sustainability indicators at once.**

| **Measures of effect size** | ***** | **Weight** | **Measuring units** | **Dependent variables in 12 CSS Models** | | | | | |
| --- | --- | --- | --- | --- | --- | --- | --- | --- | --- |
|  |  |  |  | **GDP per capita, a_1_** | **GDP per capita in PPP, a_2_** | **…** | **Democracy index, a_8_** | **…** | **Human rights and rule of law index, a_17_** |
|  |  |  |  | Q_1_ | Q_2_ | … | Q_8_ | … | Q_17_ |
| **1. CSS Environmental Performance Index Model** | | | | | | | | | |
| 1. Pearson’s correlation coefficient ( \| r \| ) | + | q^1^_1_ | Numbers | x^1^_11_ | x^1^_12_ | … | x^1^_18_ | … | x^1^_1 17_ |
| 2. Coefficient of determination (R^2^) | + | q^1^_2_ | Numbers | x^1^_21_ | x^1^_22_ | … | x^1^_28_ | … | x^1^_2 17_ |
| 3. Standardized beta coefficient (β) | + | q^1^_3_ | Estimates | x^1^_31_ | x^1^_32_ | … | x^1^_38_ | … | x^1^_3 17_ |
| 4. Standard deviation | - | q^1^_4_ | Values | x^1^_41_ | x^1^_42_ | … | x^1^_48_ | … | x^1^_4 17_ |
| 5. p values (probability level) | - | q^1^_5_ | Values | x^1^_51_ | x^1^_52_ | … | x^1^_58_ | … | x^1^_5 17_ |
| 6. Research context | + | q^1^_6_ | Expert estimates | x^1^_61_ | x^1^_62_ | … | x^1^_68_ | … | x^1^_6 17_ |
| 7. Practical significance | + | q^1^_7_ | Expert estimates | x^1^_71_ | x^1^_72_ | … | x^1^_78_ | … | x^1^_7 17_ |
| 8. Indicators with low sustainability values | + | q^1^_8_ | Expert estimates | x^1^_81_ | x^1^_82_ | … | x^1^_88_ | … | x^1^_8 17_ |
| … | … | … | … | … | … | … | … | … | … |
| **12. CSS Positive Peace Index Model** | | | | | | | | | |
| 1. Pearson’s correlation coefficient ( \| r \| ) | + | q^12^_1_ | Numbers | x^12^_11_ | x^12^_12_ | … | x^12^_18_ | … | x^12^_1 17_ |
| 2. Coefficient of determination (R^2^) | + | q^12^_2_ | Numbers | x^12^_21_ | x^12^_22_ | … | x^12^_28_ | … | x^12^_2 17_ |
| 3. Standardized beta coefficient (β) | + | q^12^_3_ | Estimates | x^12^_31_ | x^12^_32_ | … | x^12^_38_ | … | x^12^_3 17_ |
| 4. Standard deviation | - | q^12^_4_ | Values | x^12^_41_ | x^12^_42_ | … | x^12^_48_ | … | x^12^_4 17_ |
| 5. p values (probability level) | - | q^12^_5_ | Values | x^12^_51_ | x^12^_52_ | … | x^12^_58_ | … | x^12^_5 17_ |
| 6. Research context | + | q^12^_6_ | Expert estimates | x^12^_61_ | x^12^_62_ | … | x^12^_68_ | … | x^12^_6 17_ |
| 7. Practical significance | + | q^12^_7_ | Expert estimates | x^12^_71_ | x^12^_72_ | … | x^12^_78_ | … | x^12^_7 17_ |
| 8. Indicators with low sustainability values | + | q^12^_8_ | Expert estimates | x^12^_81_ | x^12^_82_ | … | x^12^_88_ | … | x^12^_8 17_ |
| **Cumulative effect size of 17 country success indicators to improve 12 sustainability indicators at once** | | | | | | | | | |
| Weight of the cumulative effect size | | | | Q_1_ | Q_2_ | … | Q_8_ | … | Q_17_ |
| Priority of the cumulative effect size | | | | P_1_ | P_2_ | … | P_8_ | … | P_17_ |
| Utility of the cumulative effect size (%) | | | | N_1_ | N_2_ | … | N_8_ | … | N_17_ |

* The sign shows that either bigger (+) or lower (−) value of the specific criterion increases/decreases the cumulative effect size to improve 12 sustainability indicators at once.
